# Supplementary material for: Synthesis and Cytotoxicity of 7,9-O-Linked Macrocyclic C-Seco Taxoids
Source: Molecules. 2019 Jun 8;24(11):2161. doi: 10.3390/molecules24112161 (PMC6600541; doi:10.3390/molecules24112161)
Supplement: Supplementary file 1 [file molecules-24-02161-s001.pdf]

Supporting information

Synthesis and Cytotoxicity of 7,9-*O*-linked Macrocylic C-seco Taxoids

Yu Zhao,[1] Tian-En Wang,[1] Alberto Mills,[2] Federico Gago,[2] and Wei-Shuo Fang[1]*

[1] State Key Laboratory of Bioactive Substances and Functions of Natural Medicines, Institute of Materia Medica, Chinese Academy of Medical Sciences & Peking Union Medical College, 2A Nan Wei Road, Beijing 100050, China.

[2] Department of Biomedical Sciences and "Unidad Asociada IQM-CSIC", School of Medicine and Health Sciences, University of Alcalá, E-28805 Alcalá de Henares, Madrid, Spain.

Table of Contents

Cell lines and culture S2

Establishment of model multidrug-resistant breast cancer cell line MCF-7/ADR..........S2

Reference S2

ESI-MS, HPLC, ^{1}H NMR and ^{13}C NMR spectra for representative compounds S3

Cell lines and culture [1]

The human breast cancer cell line MCF-7 and its doxorubicin-resistant counterpart MCF-7/ADR were provided by Xiangya Hospital, Central South University. Hela and Hela/βIII cells were a generous gift from Dr. Richard Ludeña at the University of Texas. MCF-7 and MCF-7/ADR were cultured in RPMI 1640 supplemented with 10% fetal bovine serum, 100 units/mL penicillin and 100 μg/mL streptomycin. To maintain the cell drug resistance phenotype, 1 μg/mL doxorubicin was added to the culture of MCF-7/ADR. Hela and Hela/βIII cells were cultured in DMEM supplemented with 10% fetal bovine serum, 100 units/mL penicillin and 100 μg/mL streptomycin, and Hela/βIII cell line was additionally supplemented with 0.5 mg/mL G418 sulfate. All cells were cultured at 37°C in a humidified atmosphere containing 5% CO2.

Establishment of model multidrug-resistant breast cancer cell line MCF-7/ADR

The multidrug-resistant breast cancer cell line MCF-7/ADR was established by exposure to doxorubicin (adriamycin) using continuous induction with step-wise increasing drug concentrations. MCF-7 cells from the logarithmic growth period were inoculated in culture medium (10% serum) in the presence of 0.005 μg/mL doxorubicin (1/10 of the IC_{50} value for cytotoxicity towards MCF-7 cells). After exposure to drug for 48 h, the culture medium was replaced with fresh medium without doxorubicin). When cells returned to normal growth, the procedure was repeated. Depending on the status of cell growth, the inducing concentration of drug was gradually increased step-wise. After culturing for 72 weeks, the MCF-7/ADR cell line model was established, which can grow normally in culture medium containing 1 μg/mL doxorubicin.

Reference

S1. Cai, P.; Lu, P.; Sharom, F.J.; Fang, W.S.. A semisynthetic taxane Yg-3-46a effectively evades P-glycoprotein and beta-III tubulin mediated tumor drug resistance in vitro. Cancer Lett., 2013, 341, 214-223. [https://doi.org/10.1016/j.canlet.2013.08.010]

ESI-MS, ^{1}H-NMR and ^{13}C-NMR spectra for representative compounds

^{1}H NMR spectra of compound **14a**

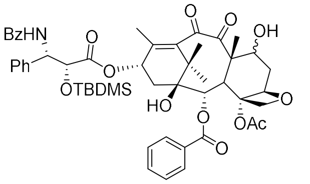

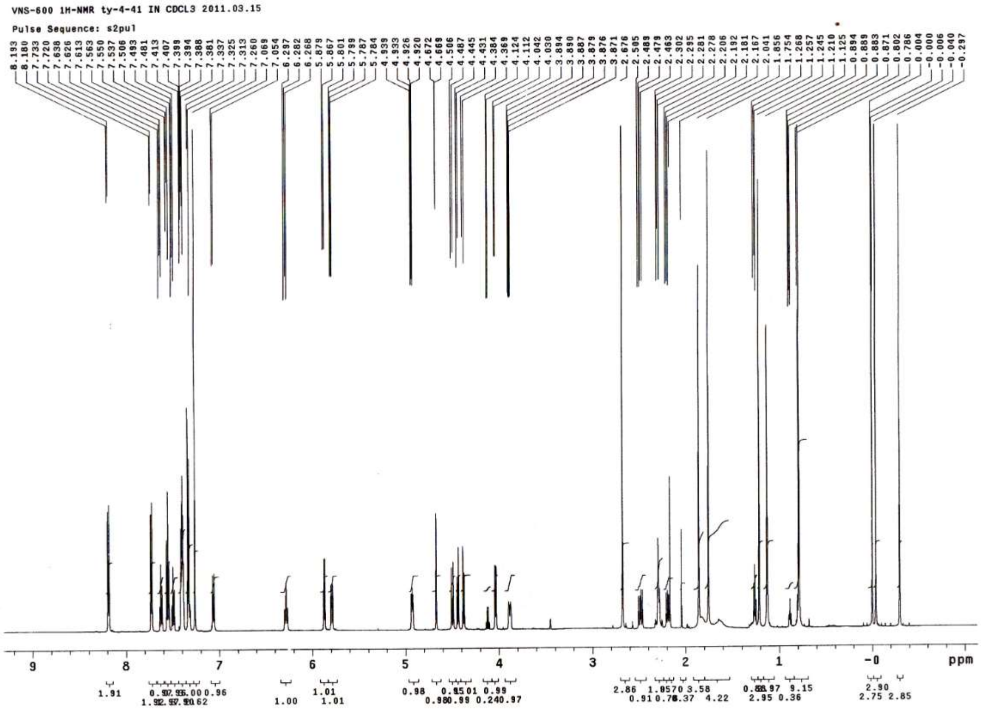

ESI-MS, ^{1}H-NMR and ^{13}C-NMR spectra of compound **14b**

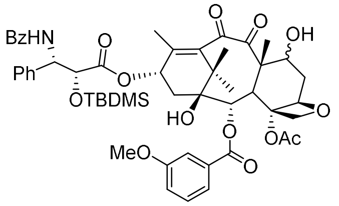

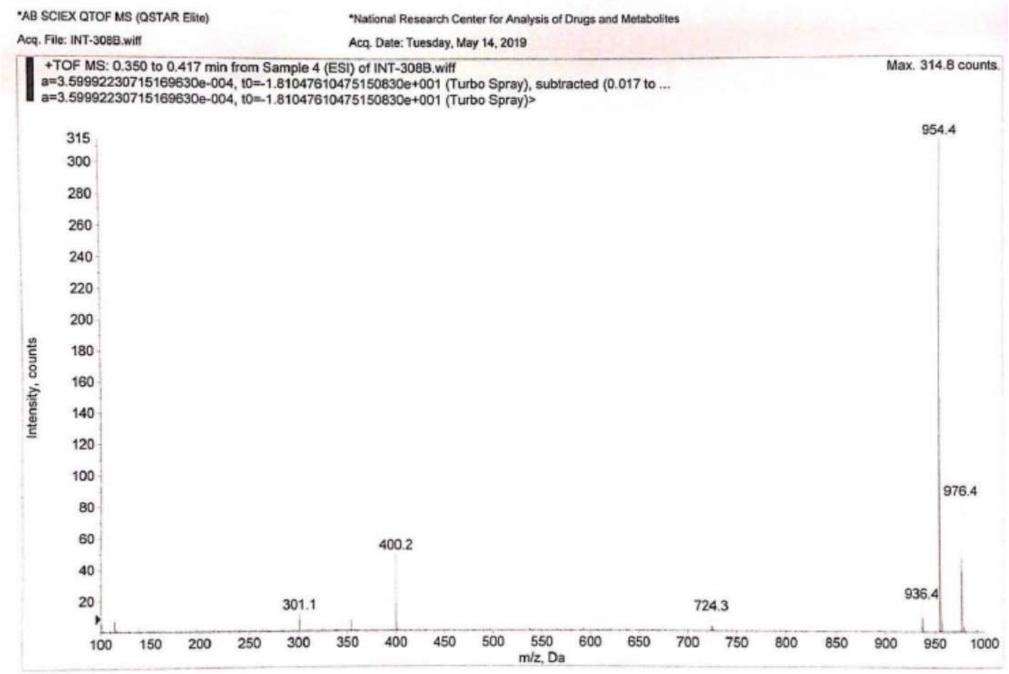

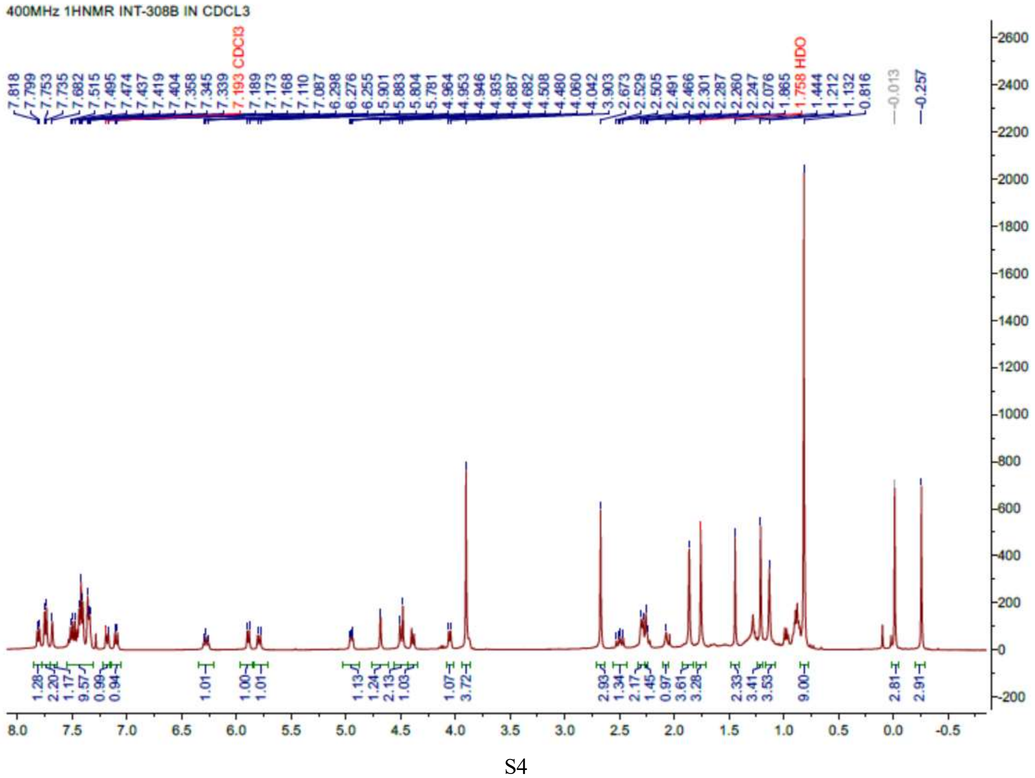

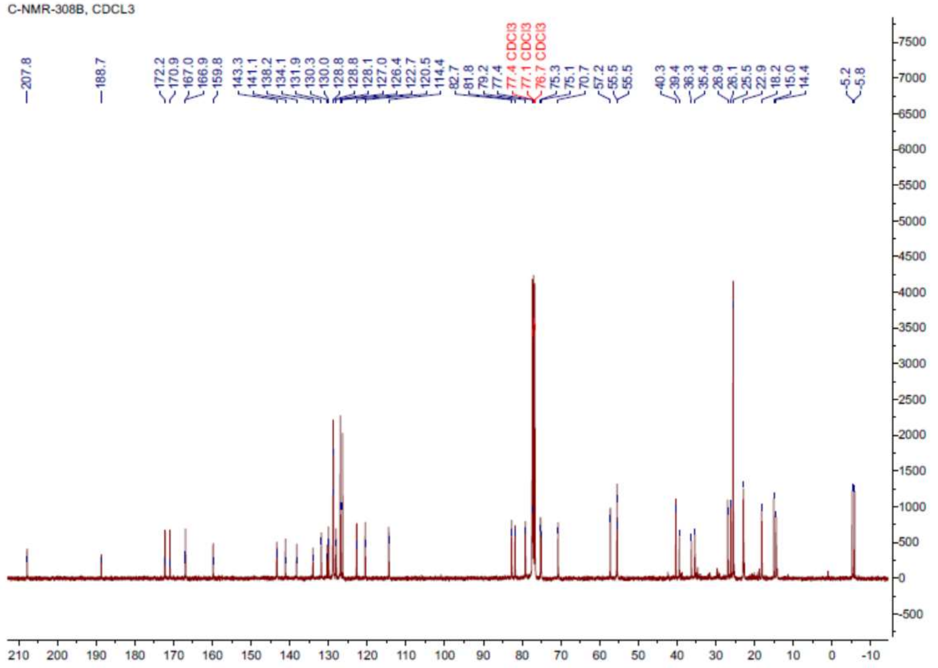
C-NMR-308B, CDCL3

ESI-MS, ^{1}H-NMR and ^{13}C-NMR spectra of compound **14c**

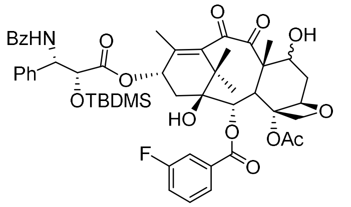

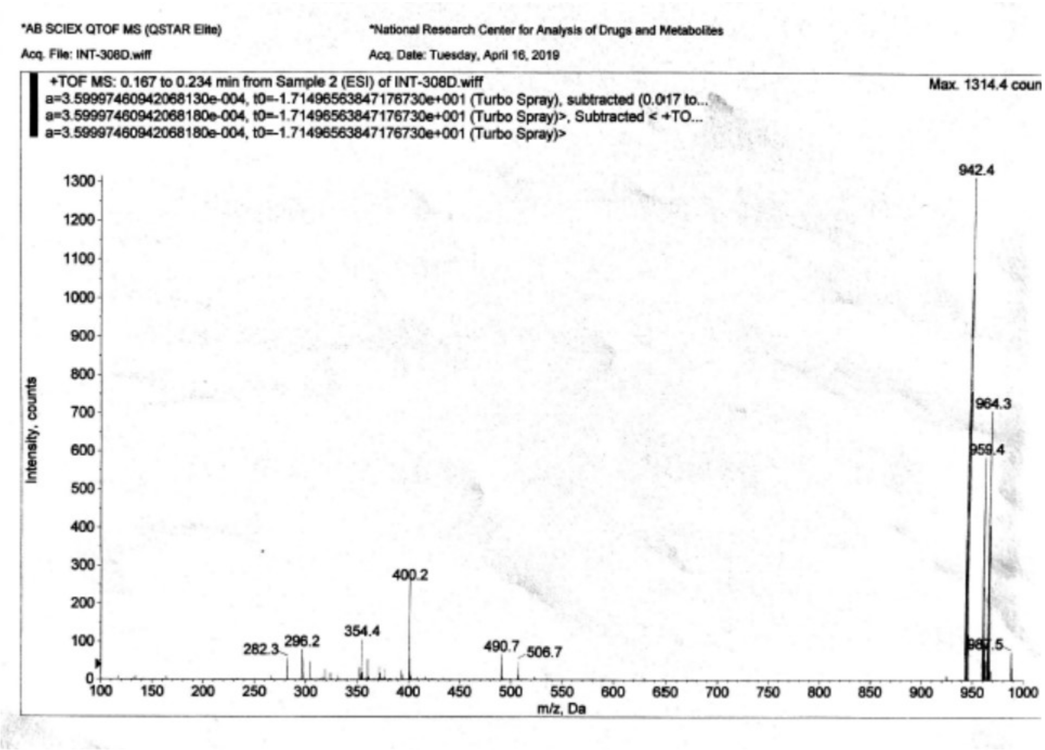

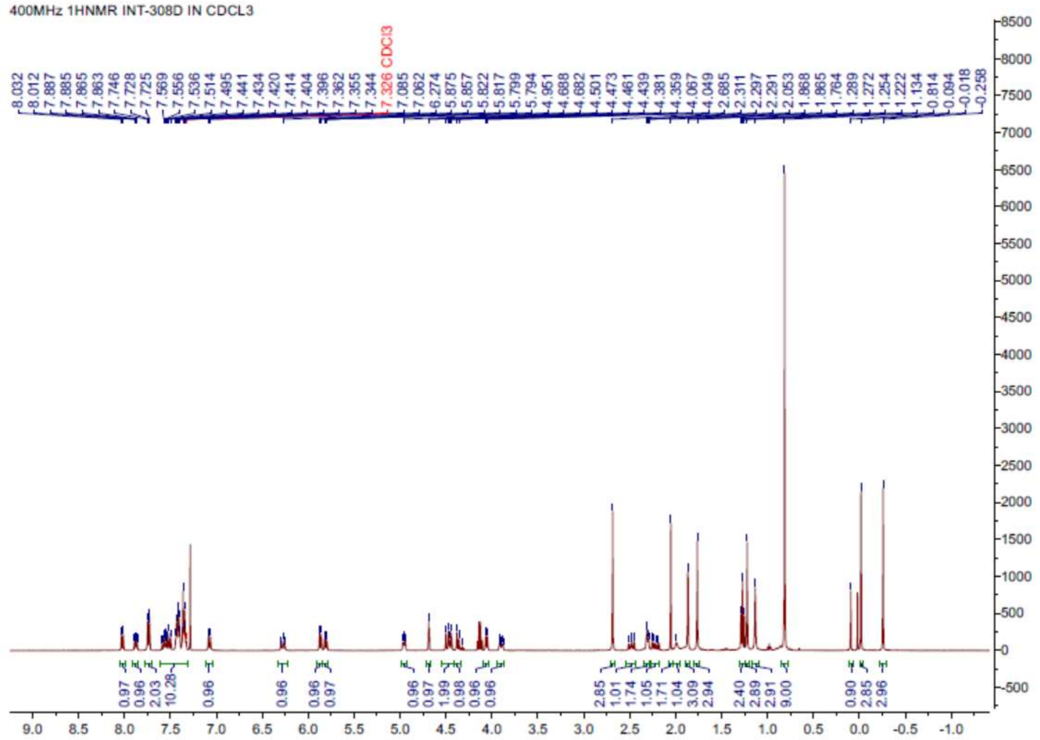

400MHz 1HNMR INT-308D IN CDCL3
CDCl3

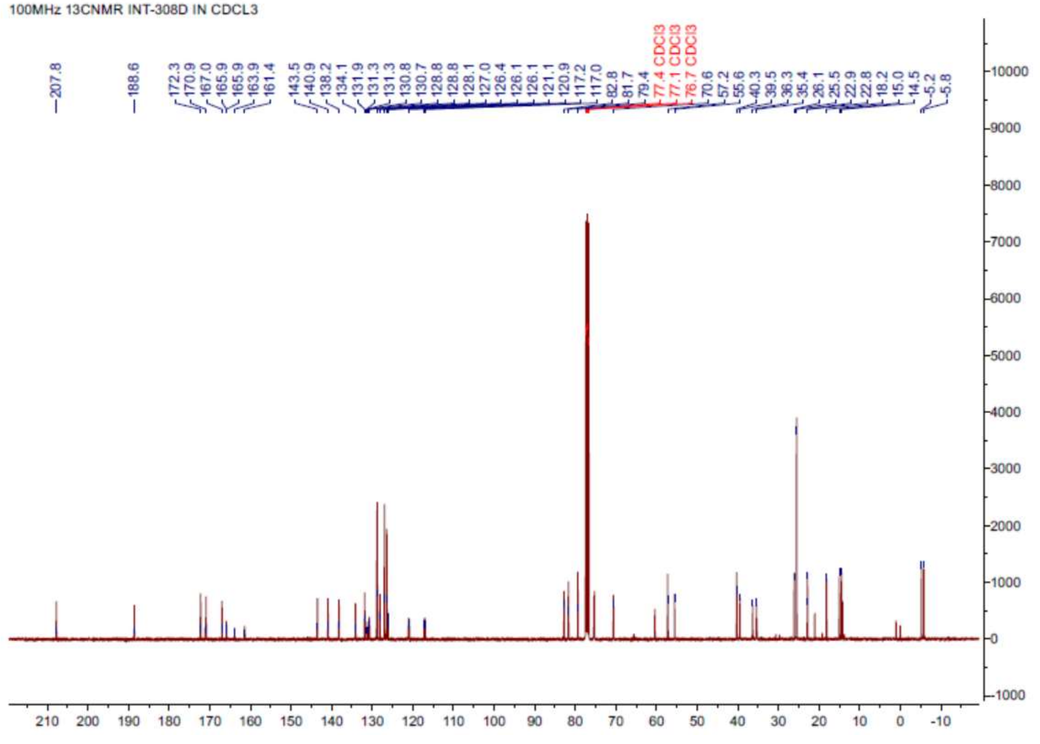

100MHz 13CNMR INT-308D IN CDCL3
CDCl3

ESI-MS, ^{1}H-NMR and ^{13}C-NMR spectra of compound **14d**

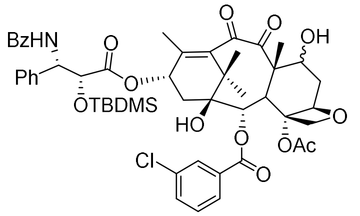

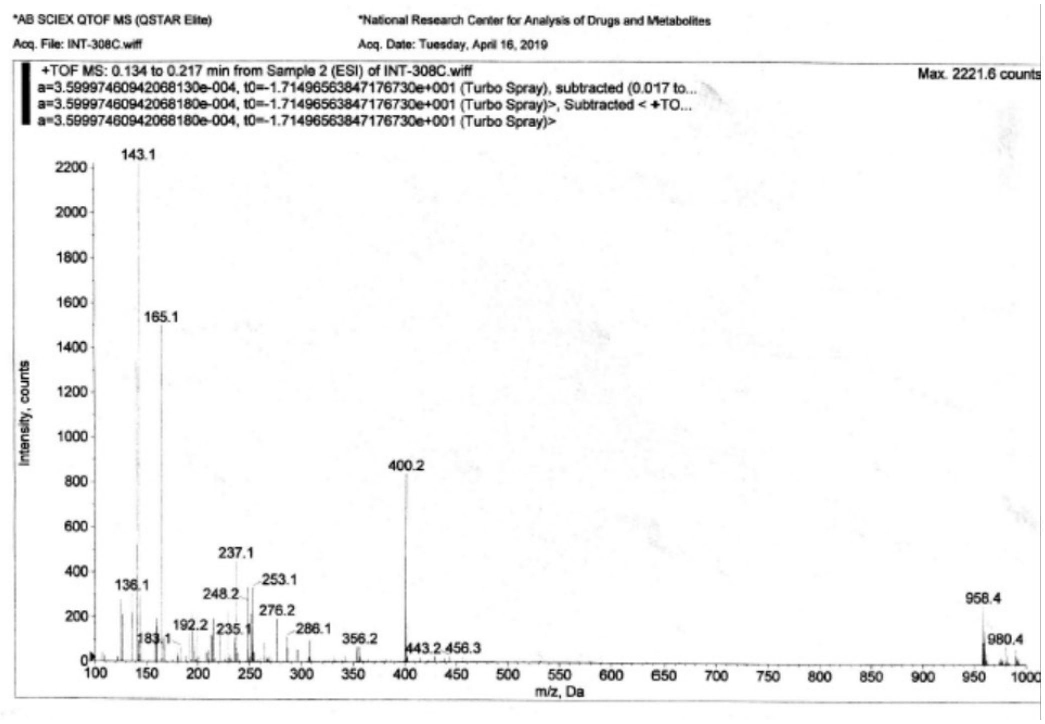

400MHz 1HNMR INT-308C IN CDCL3

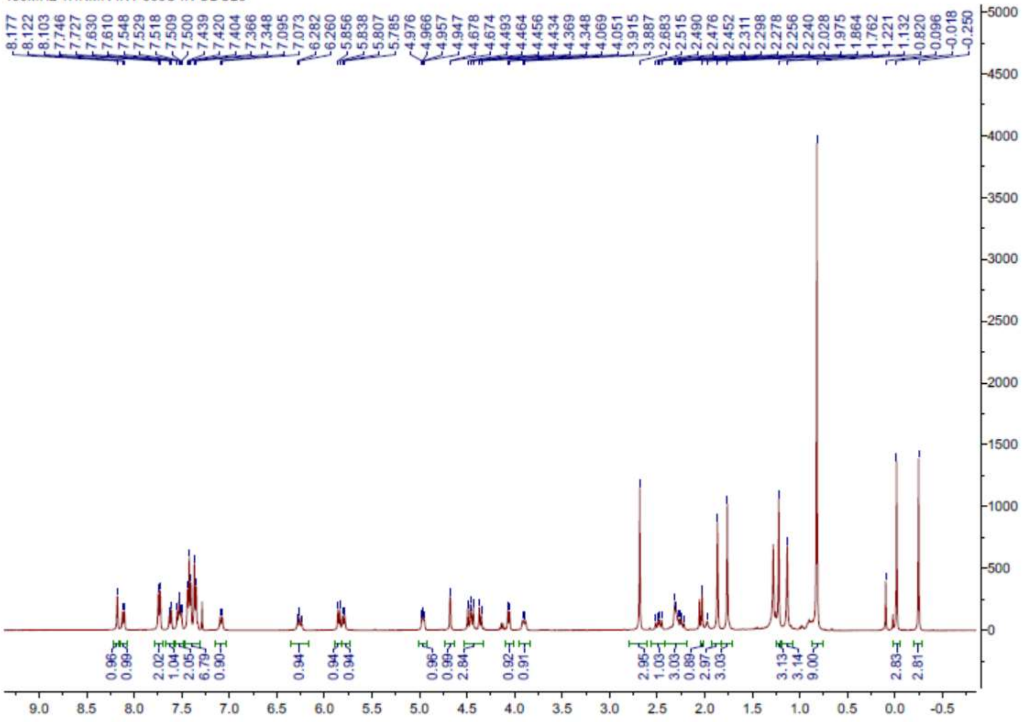

100MHz 13CNMR INT-308C IN CDCL3

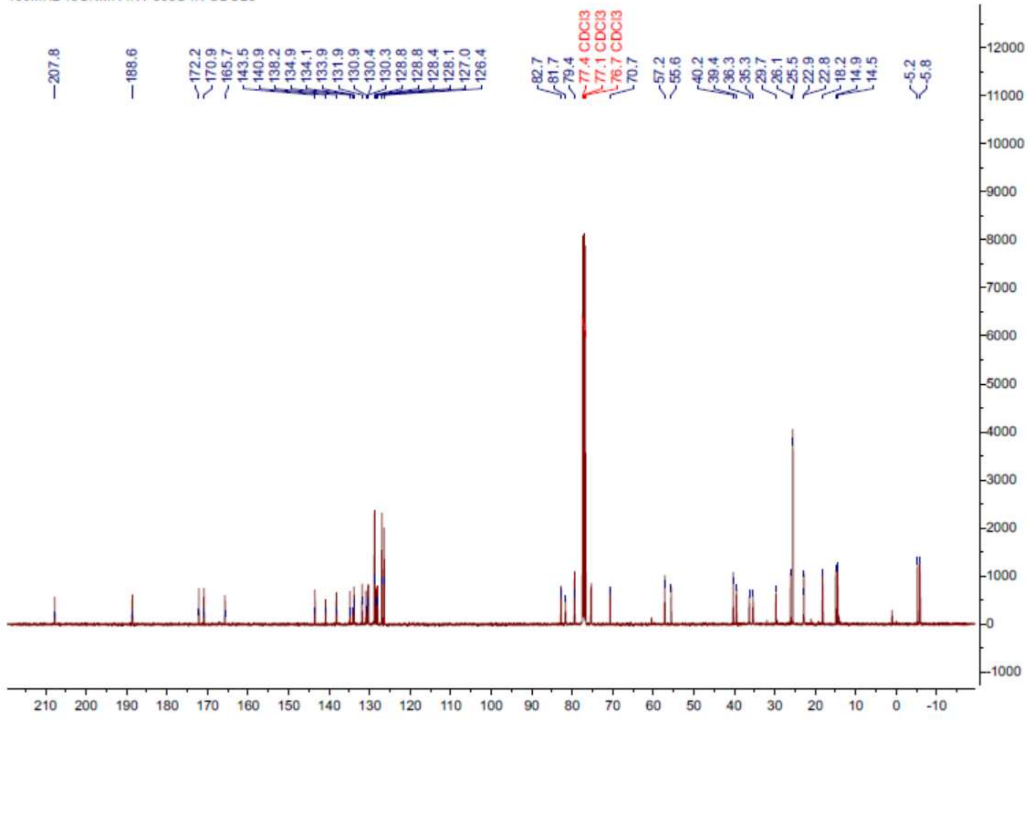

ESI-MS, ^{1}H-NMR and ^{13}C-NMR spectra of compound **14e**

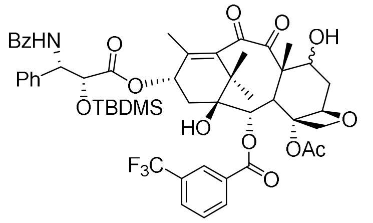

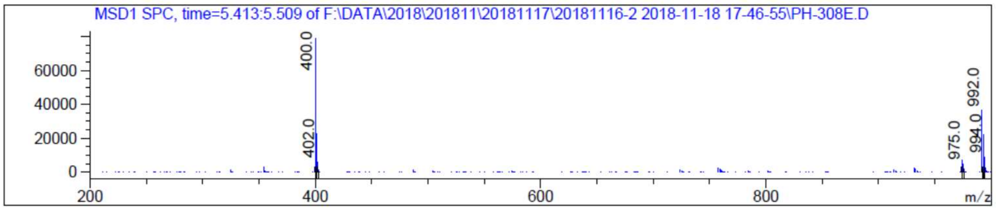

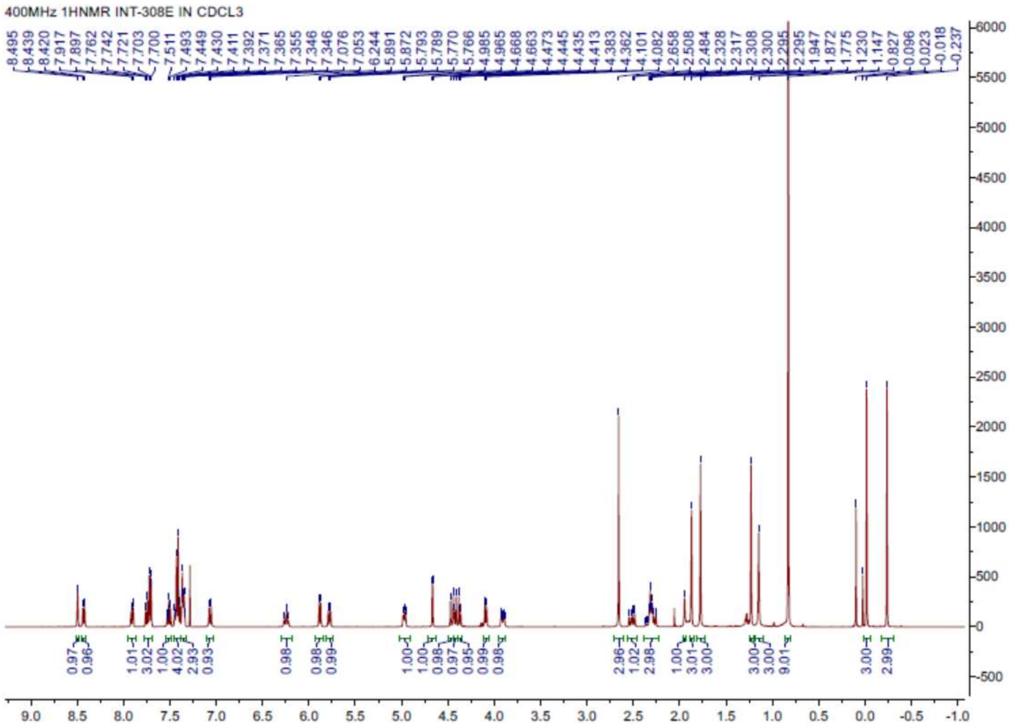

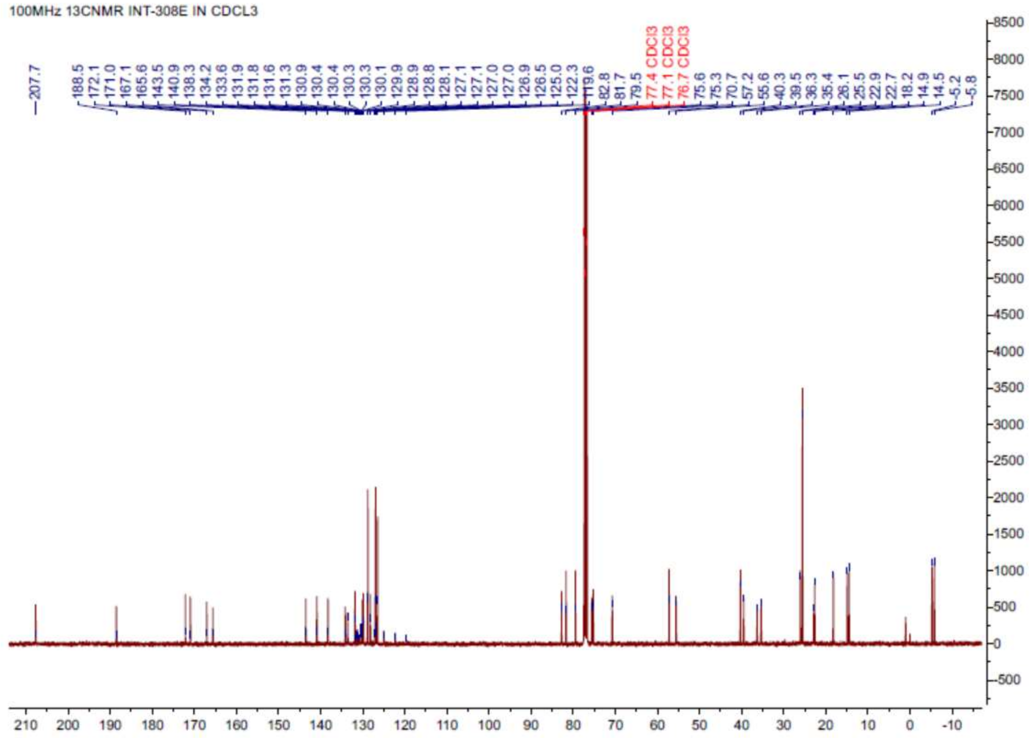

100MHz 13CNMR INT-308E IN CDCL3

ESI-MS, ^{1}H-NMR and ^{13}C-NMR spectra of compound **15b**

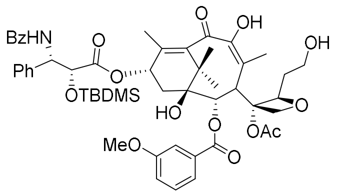

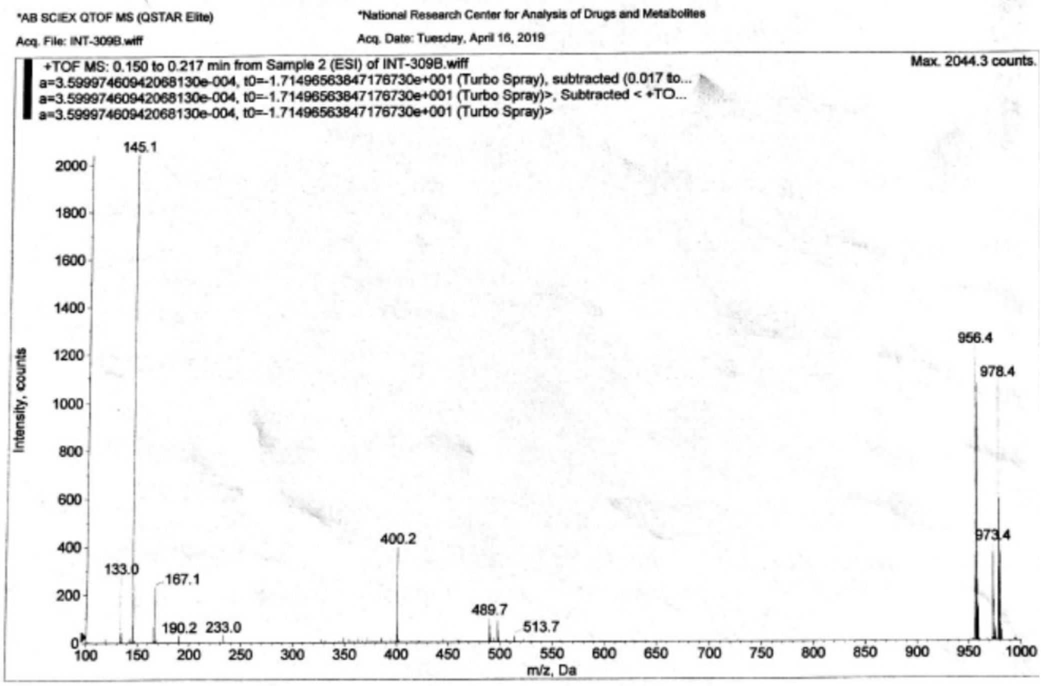

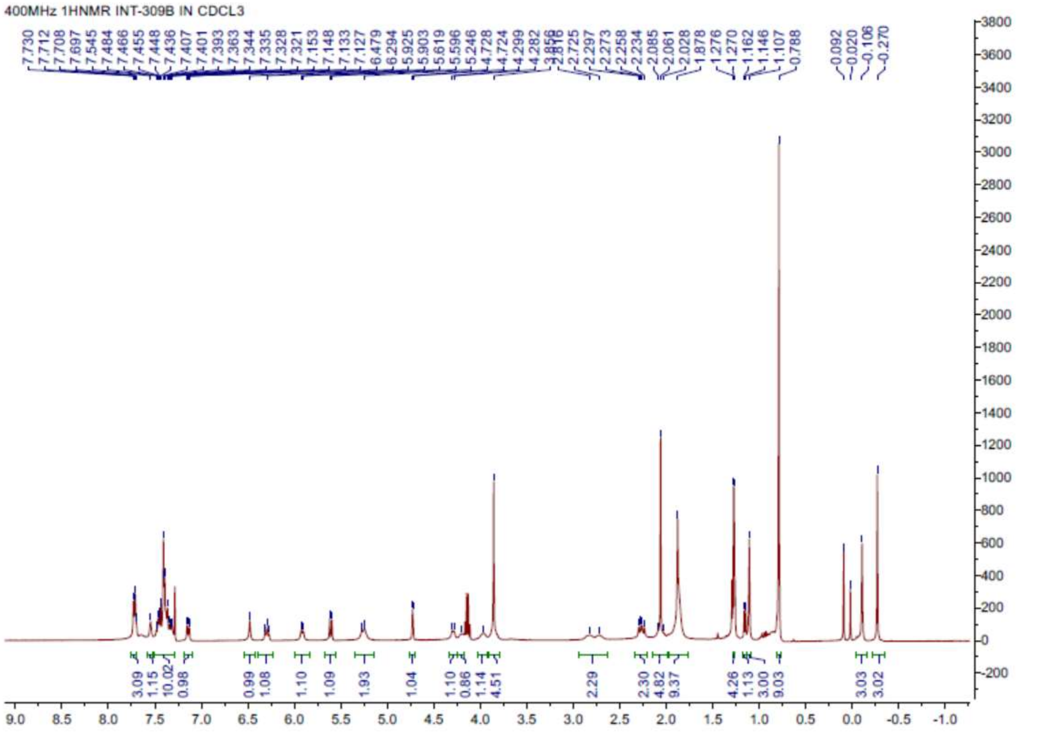

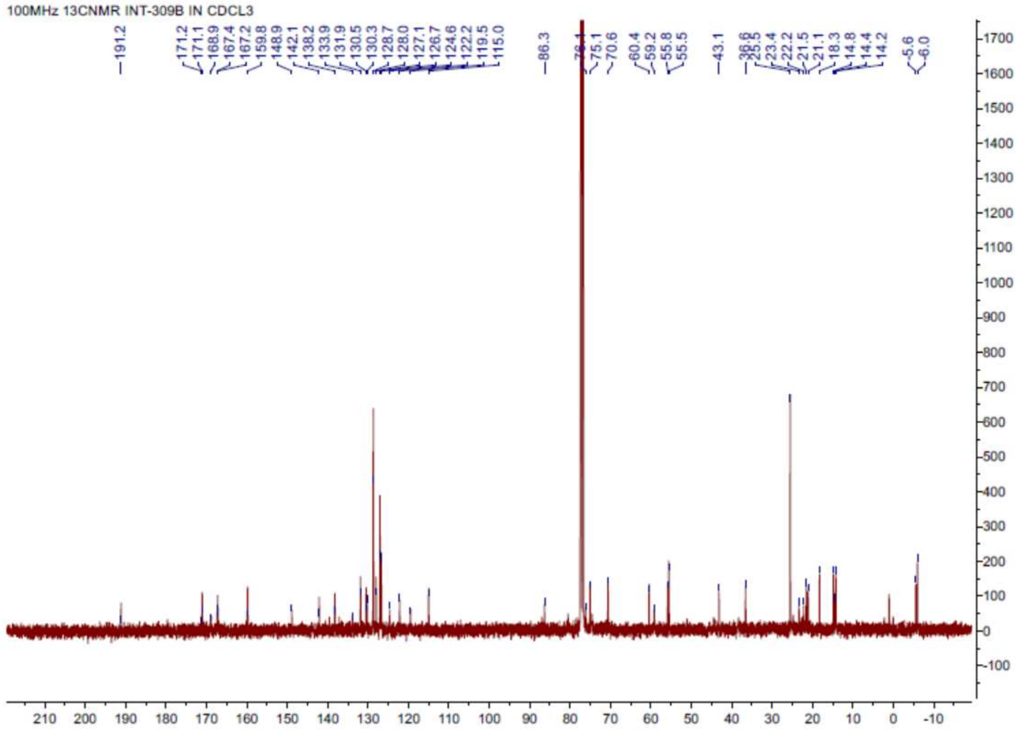
100MHz 13CNMR INT-309B IN CDCL3

ESI-MS, ^{1}H-NMR and ^{13}C-NMR spectra of compound **15c**

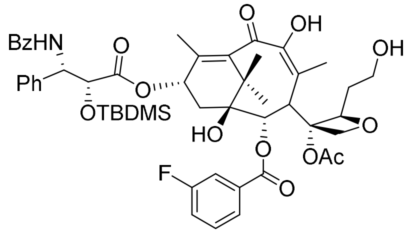

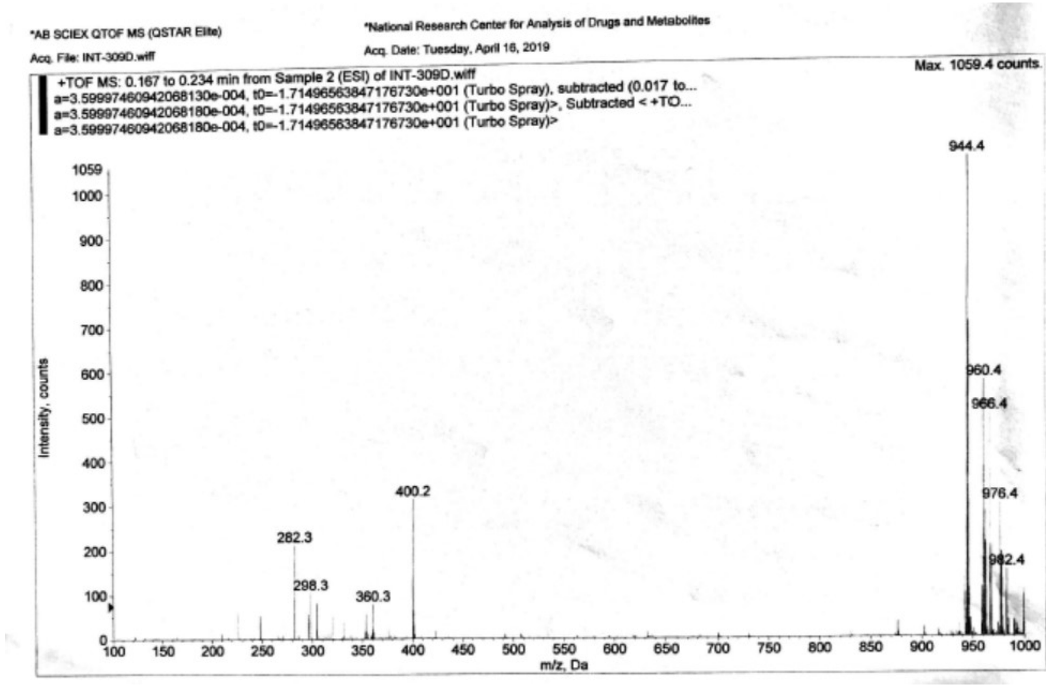

400MHz 1HNMR INT-309D IN CDCL3

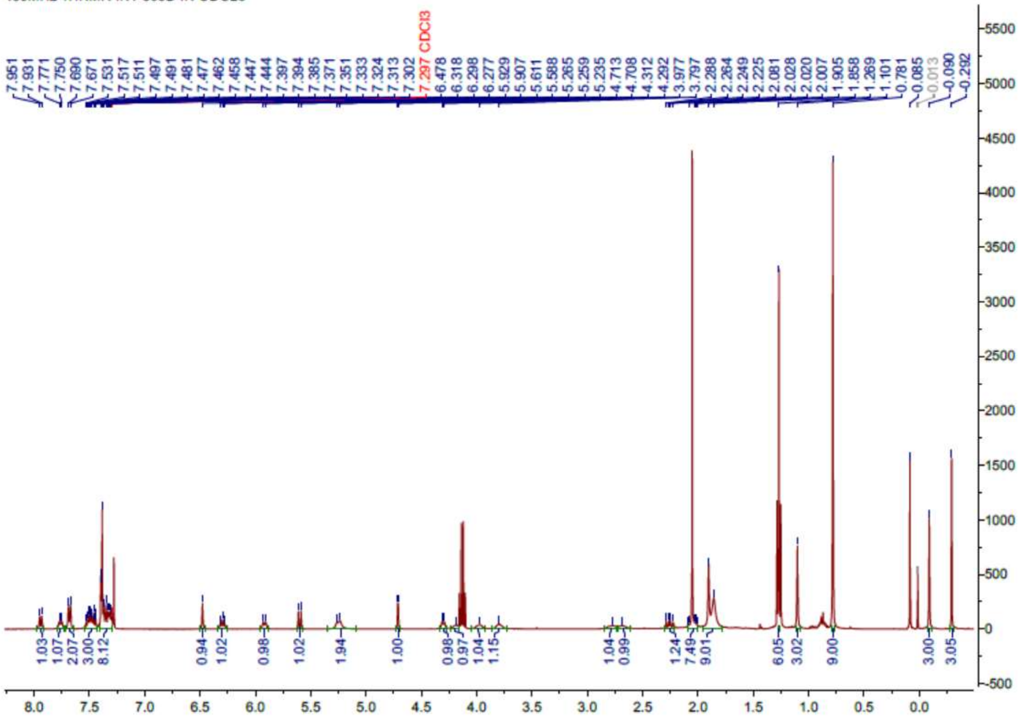

100MHz 13CNMR INT-309D IN CDCL3

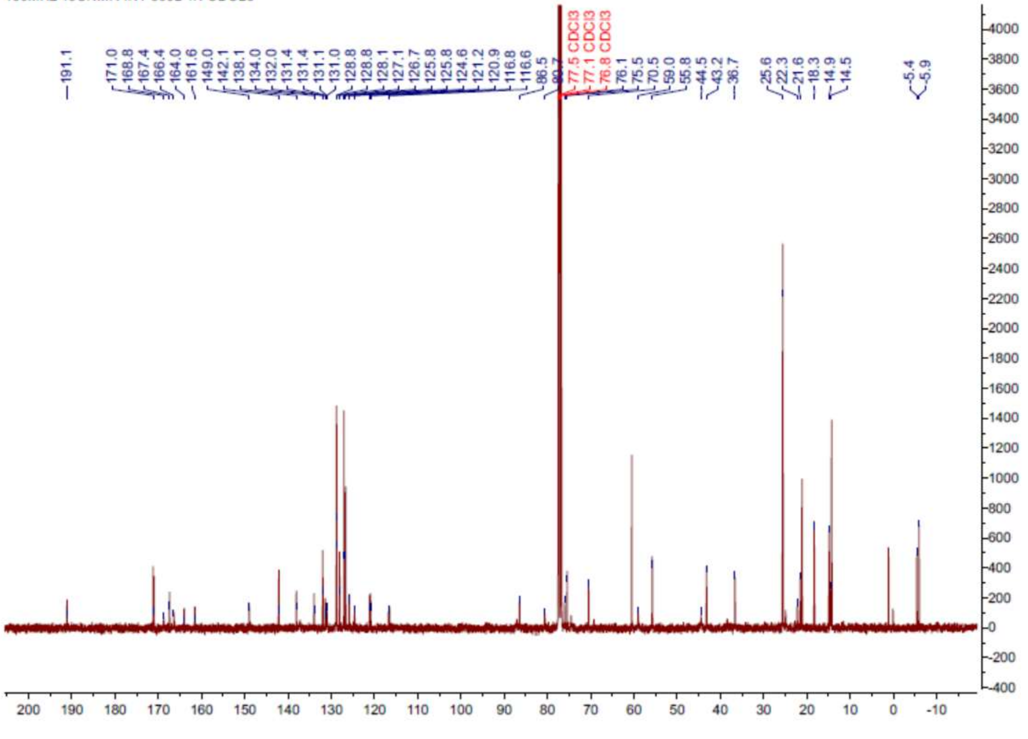

ESI-MS, ^{1}H-NMR and ^{13}C-NMR spectra of compound **15d**

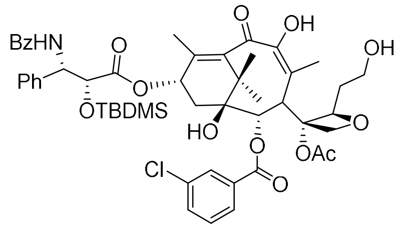

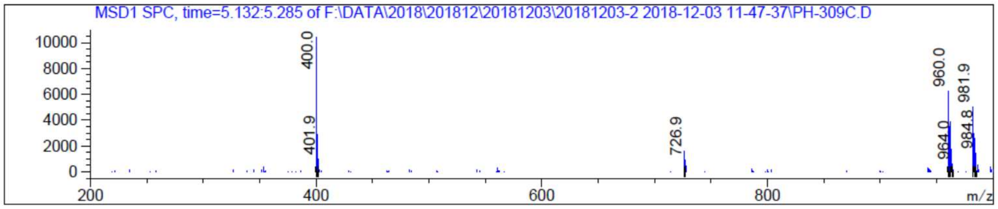

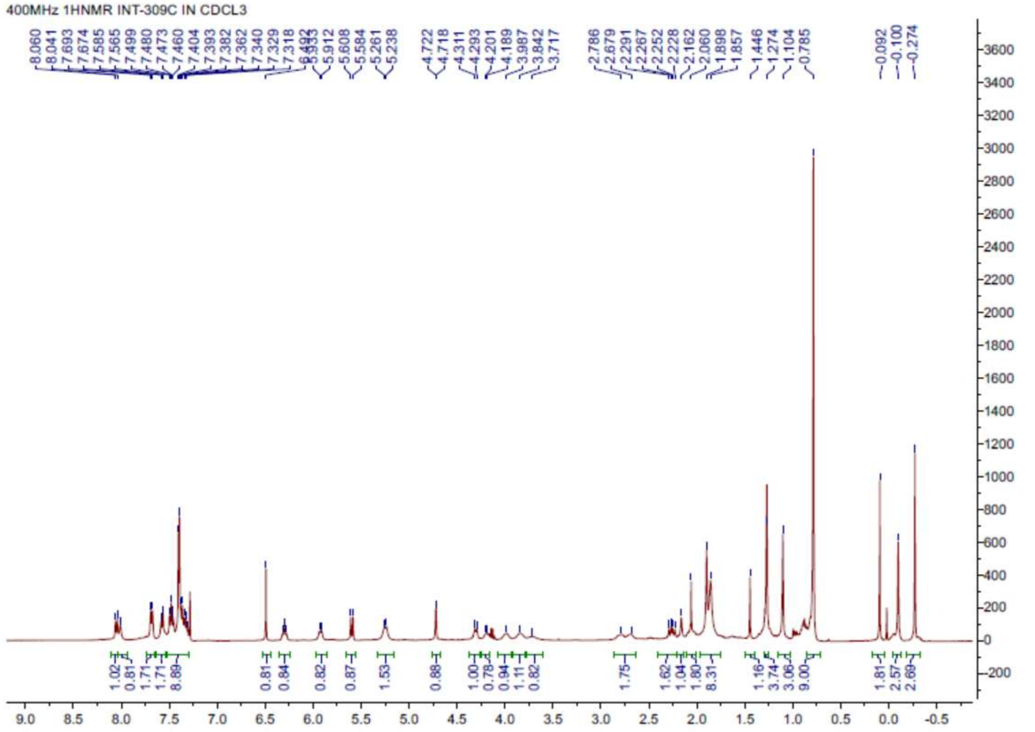

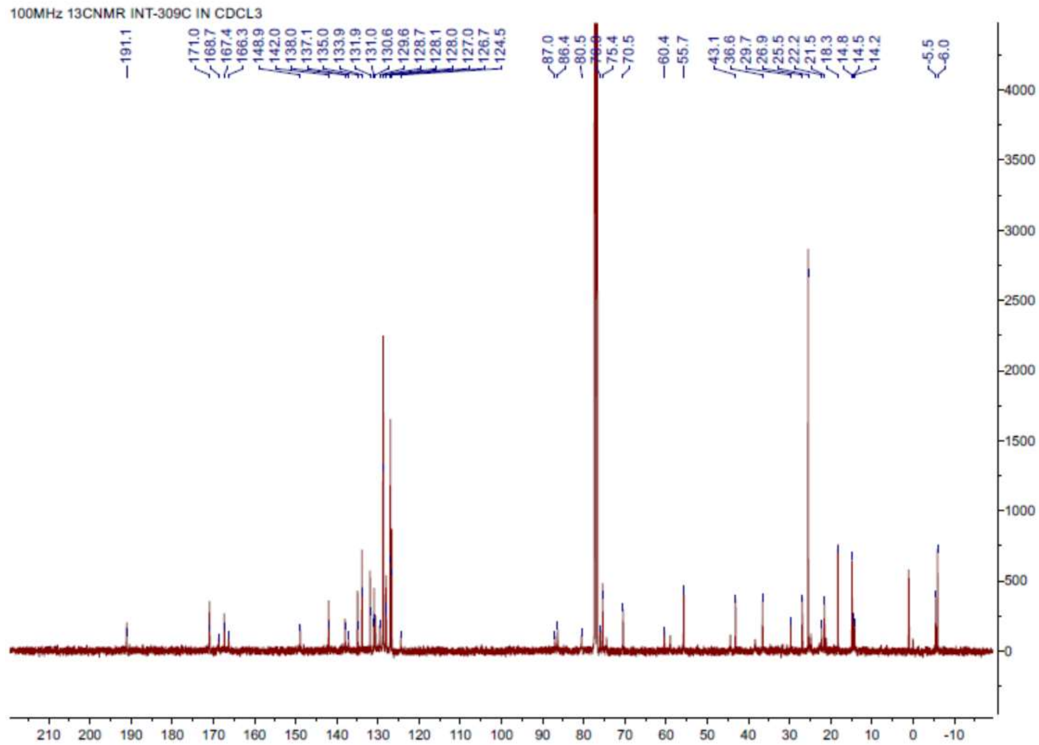

100MHz 13CNMR INT-309C IN CDCL3

ESI-MS, ^{1}H-NMR and ^{13}C-NMR spectra of compound **15e**

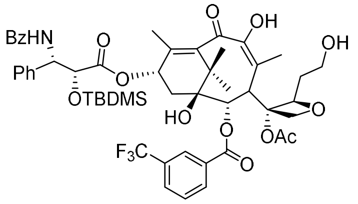

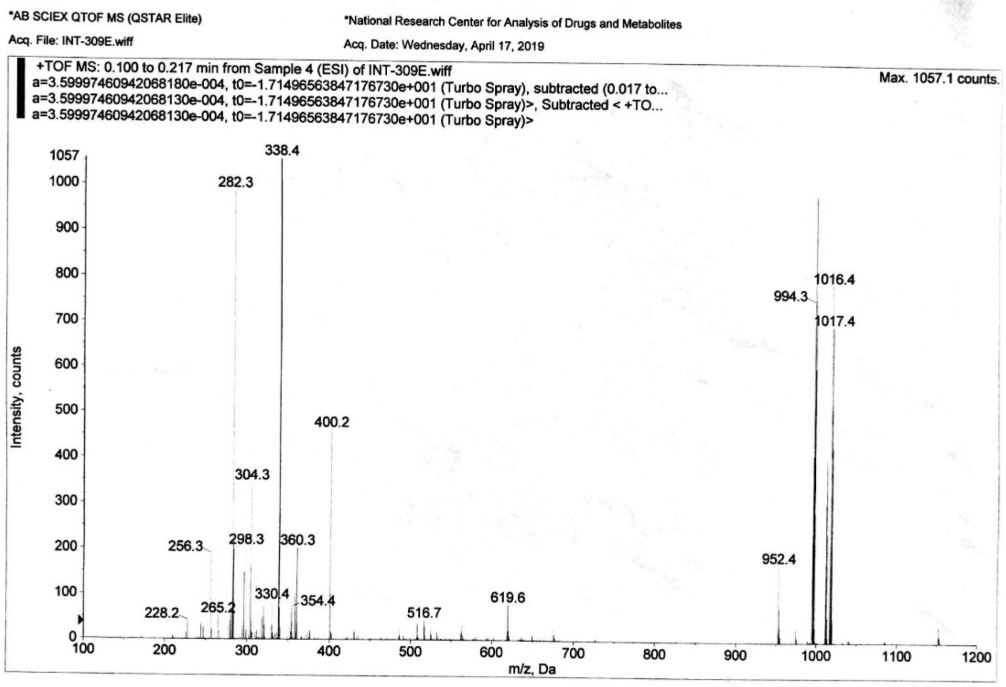

400MHz 1HNMR INT-309E IN CDCL3

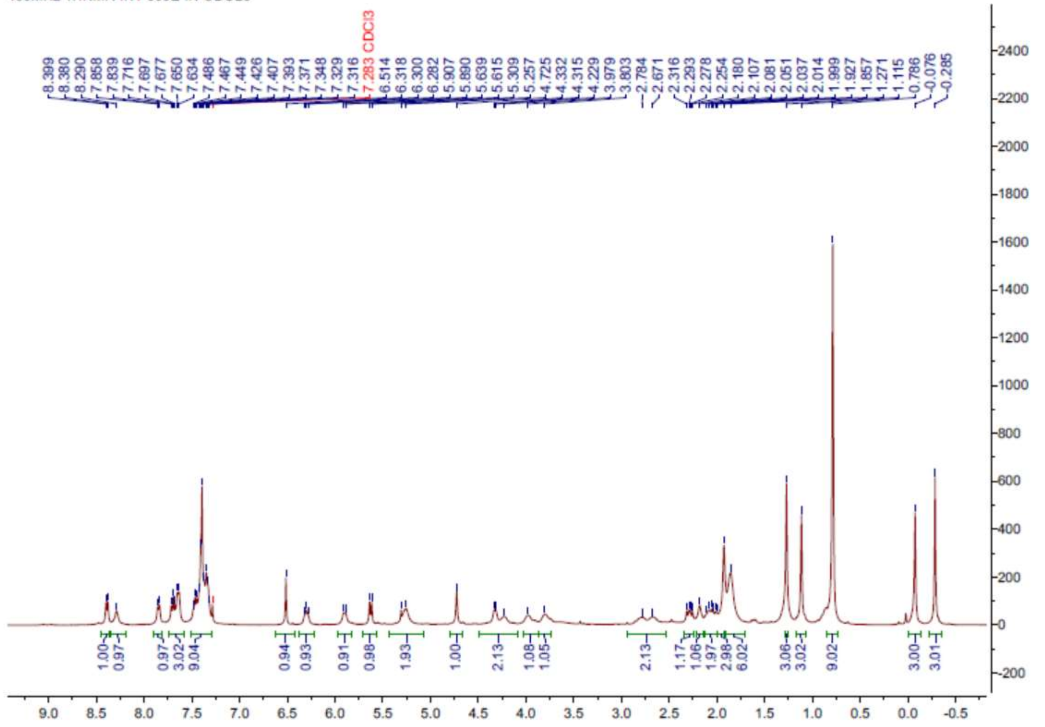

100MHz 13CNMR INT-309E IN CDCL3

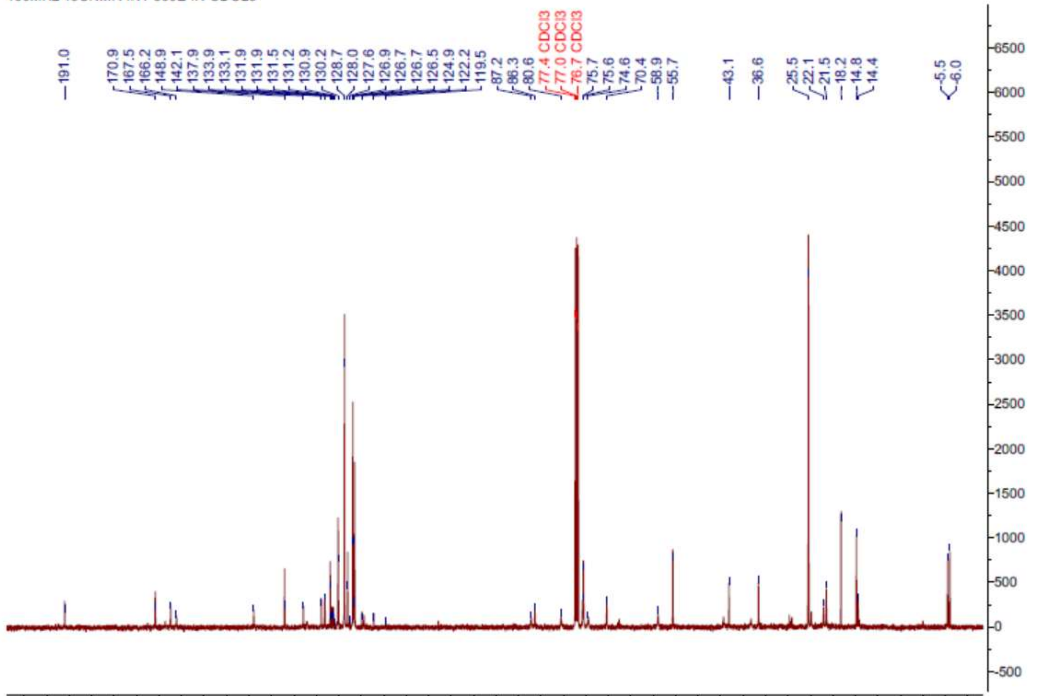

ESI-MS, HPLC, ^{1}H NMR and ^{13}C NMR spectra of compound **16a**

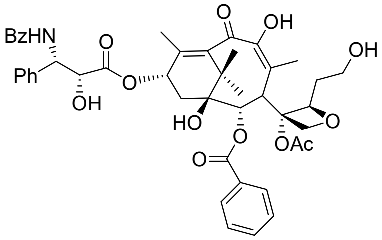

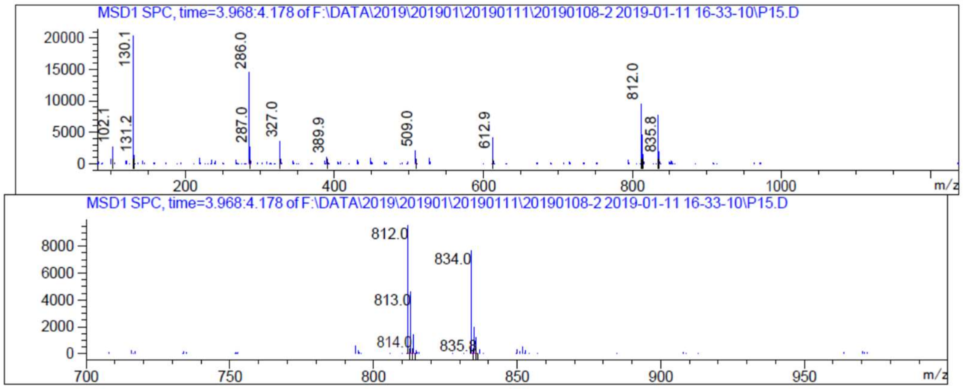

```
Sample Name       : P15
Injection Date    : Fri, 11. Jan. 2019                    Seq. Line :   1
Inj. Volumn       : 3.0  ul
Acq Operator      :
Acq   Method      : F:\DATA\2019\201901\20190111\20190108-2 2019-01-11 16-33-10\MTH-GEN-
                    MS.M
                    HPLC Analysis Conditions
1. Column        : XDB C18  4.6*50mm  1.8um
2. Mobile Phase :  A:Water+0.05%TFA    B :ACN+0.05%TFA
                   Time              A%                  B%
3. Flow Mode     : 0.00              90.0                10.0
                   4.50              0.0                 100.0
                   6.50              0.0                 100.0
                   6.60              90.0                10.0
 4. Flow         :   Start Flow :  1.0  ml/min
 5. UV Wavelength:   C: 220nm ;
 6. Column Temp. :   Left :    40.0C    Right :     40.0C
 7. Sample Preparation:
```

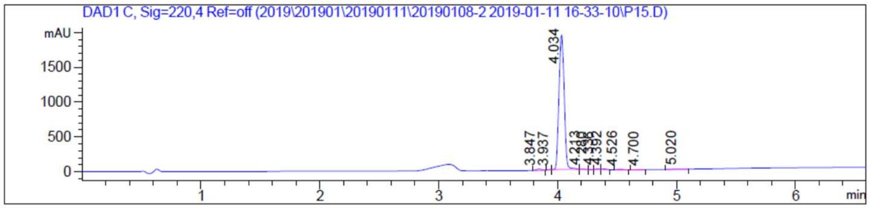

#	Meas. Ret. Time	Height	Height %	Area	Area %
1	3.847	19.160	0.955	54.417	0.895
2	3.937	2.686	0.134	6.574	0.108
3	4.034	1944.258	96.875	5908.744	97.216
4	4.213	5.175	0.258	11.531	0.190
5	4.280	1.172	0.058	1.535	0.025
6	4.336	6.235	0.311	11.735	0.193
7	4.392	8.282	0.413	17.960	0.295
8	4.526	11.149	0.556	31.937	0.525
9	4.700	3.612	0.180	13.318	0.219
10	5.020	5.247	0.261	20.221	0.333

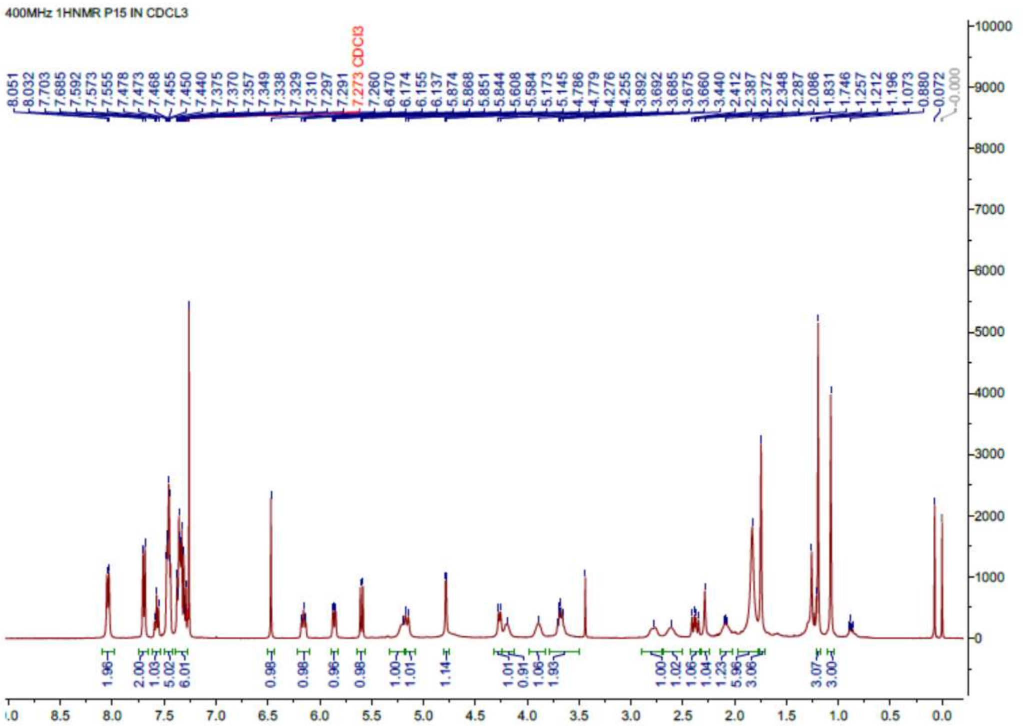
400MHz 1HNMR P15 IN CDCL3
7.273 CDCl3

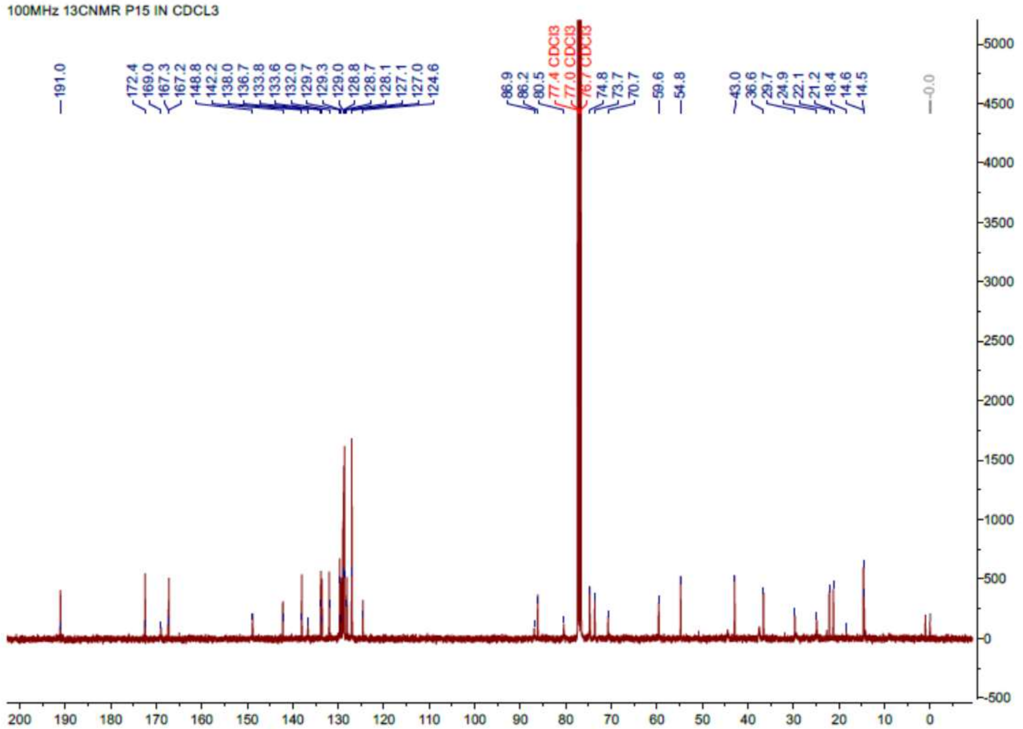
100MHz 13CNMR P15 IN CDCL3
77.4 CDCl3
77.0 CDCl3
76.7 CDCl3

ESI-MS, HPLC, ^{1}H NMR and ^{13}C NMR spectra of compound **16b**

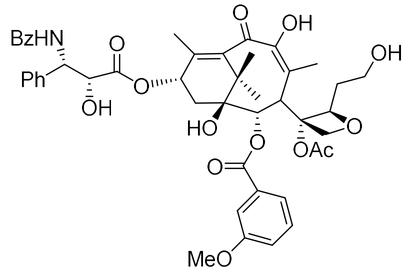

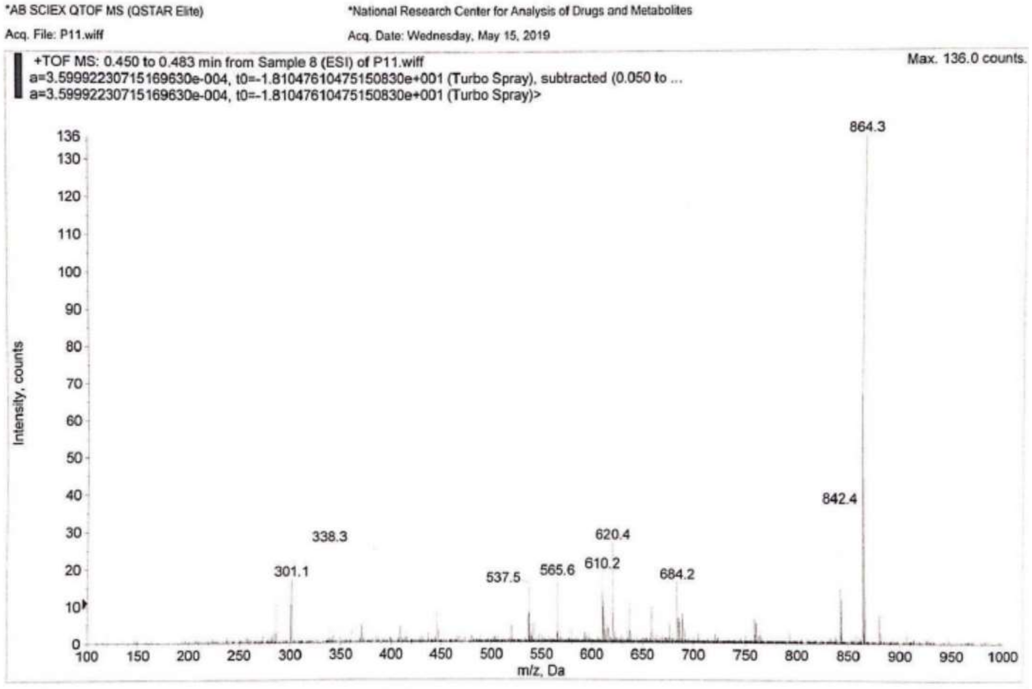

Sample Name : P11
Injection Date : Fri, 30. Nov. 2018 Seq. Line : 7
Inj. Volumn : 10.0 ul
Acq Operator :
Acq Method : F:\DATA\2018\201811\20181130\20181130-1 2018-11-30 15-34-15\MTH-GEN-MS.M
HPLC Analysis Conditions
1. Column : XDB C18 4.6*50mm 1.8um
2. Mobile Phase : A:Water+0.05%TFA B :ACN+0.05%TFA

	Time	A%	B%
3. Flow Mode :	0.00	90.0	10.0
	4.50	0.0	100.0
	6.50	0.0	100.0
	6.60	90.0	10.0

4. Flow : Start Flow : 1.2 ml/min
5. UV Wavelength: C: 220nm ;
6. Column Temp. : Left : 40.0C Right : 40.0C
7. Sample Preparation:

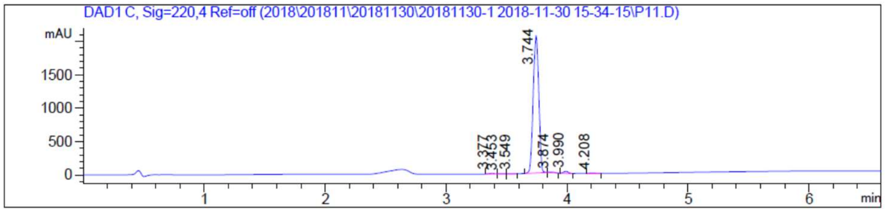

#	Meas. Ret. Time	Height	Height %	Area	Area %
1	3.377	8.992	0.423	24.694	0.369
2	3.453	2.653	0.125	6.044	0.090
3	3.549	5.269	0.248	13.300	0.199
4	3.744	2062.169	96.944	6521.324	97.398
5	3.874	9.349	0.440	26.006	0.388
6	3.990	30.292	1.424	80.850	1.208
7	4.208	8.442	0.397	23.357	0.349

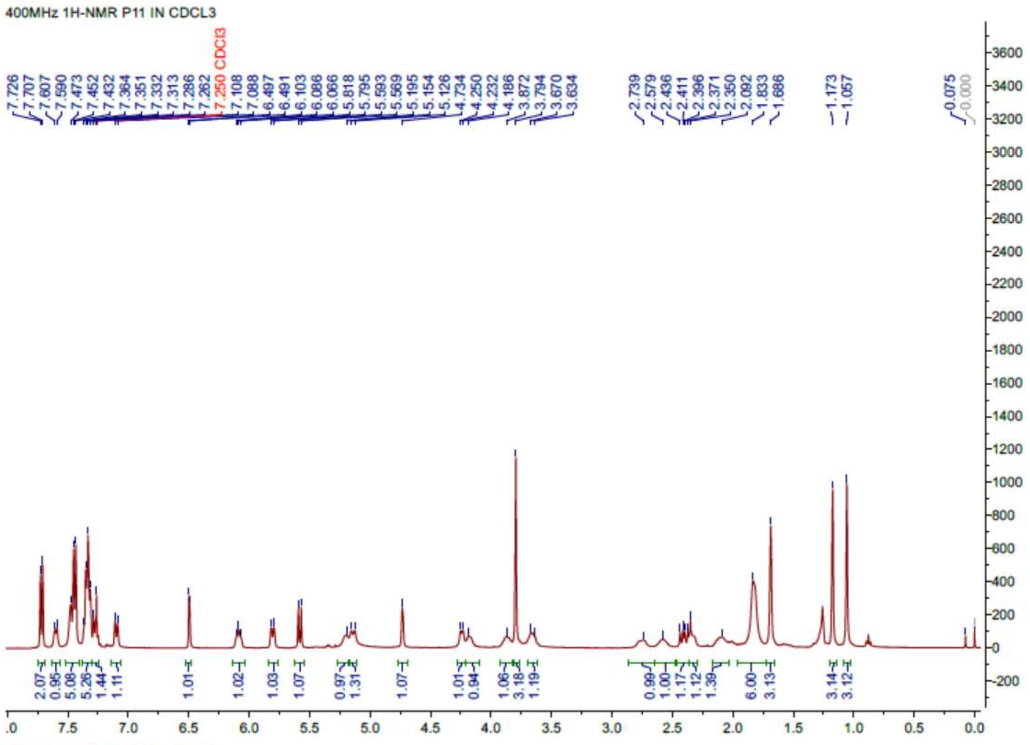
400MHz 1H-NMR P11 IN CDCL3

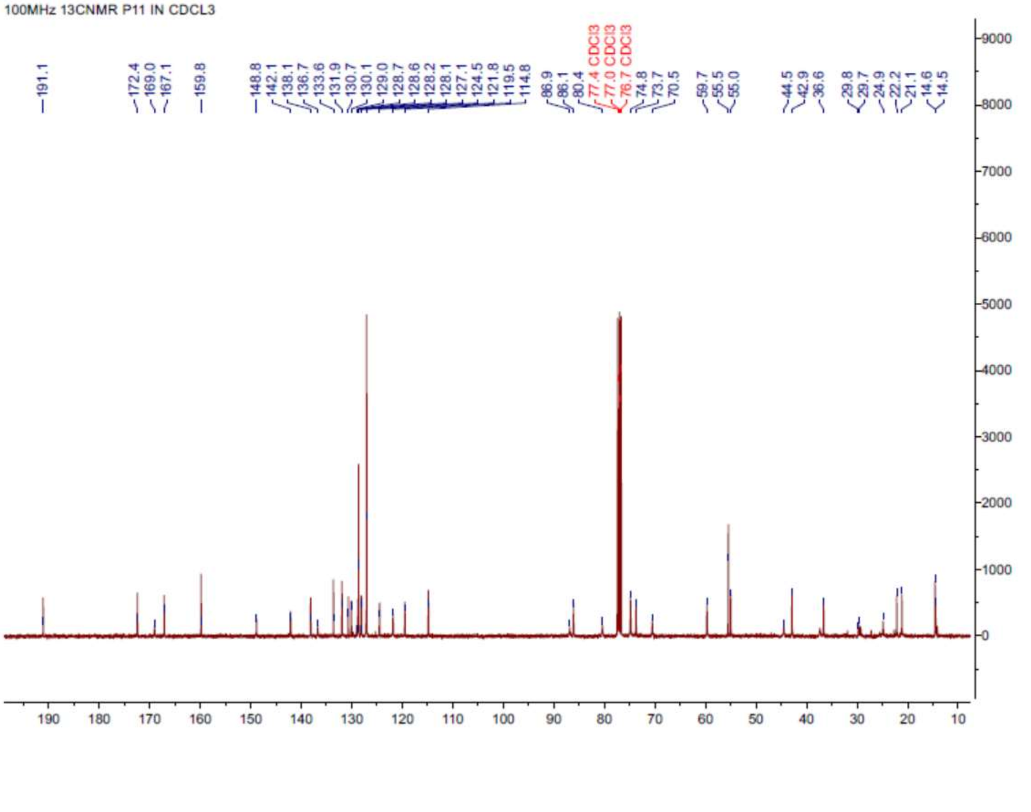
100MHz 13CNMR P11 IN CDCL3

ESI-MS, HPLC, ^{1}H NMR and ^{13}C NMR spectra of compound **16c**

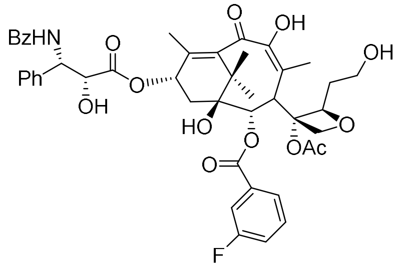

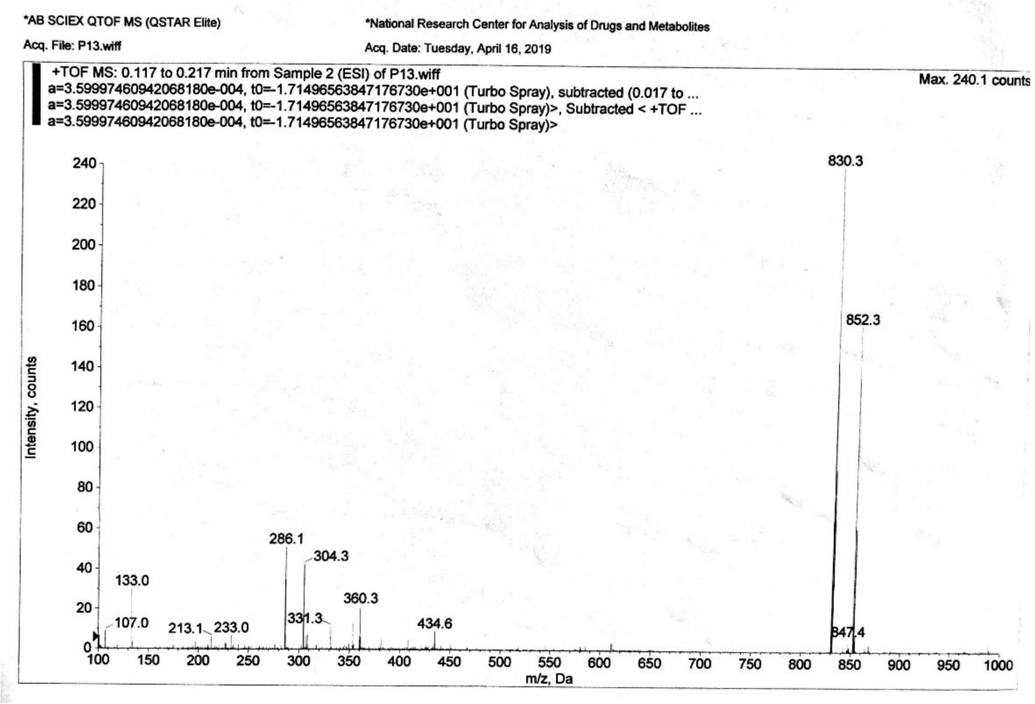

```
Sample Name       : P13
Injection Date    : Fri, 7. Dec. 2018                        Seq. Line :   6
Inj. Volumn       : 10.0 ul
Acq Operator      :
Acq   Method      : F:\DATA\2018\201812\20181205\20181206-3 2018-12-07 17-31-08\MTH-GEN-
                    MS.M
                    HPLC Analysis Conditions

1. Column        : XDB C18  4.6*50mm  1.8um
2. Mobile Phase :  A:Water+0.05%TFA    B :ACN+0.05%TFA
                  Time              A%                  B%
3. Flow Mode     : 0.00             90.0                10.0
                  4.50              0.0                 100.0
                  6.50              0.0                 100.0
                  6.60              90.0                10.0
 4. Flow          :   Start Flow :  1.0  ml/min

 5. UV Wavelength:   C: 220nm ;

 6. Column Temp. :   Left :   40.0C    Right :     40.0C

 7. Sample Preparation:
```

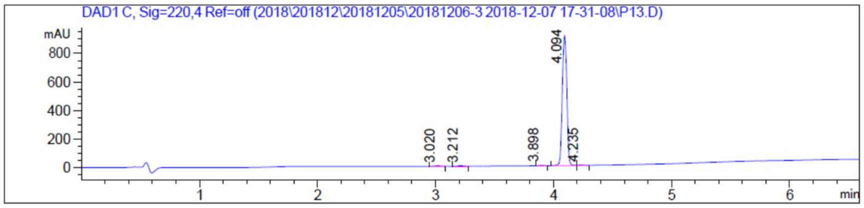

#	Meas. Ret. Time	Height	Height %	Area	Area %
1	3.020	4.379	0.468	14.777	0.595
2	3.212	4.599	0.491	13.769	0.554
3	3.898	3.676	0.393	9.549	0.384
4	4.094	920.609	98.320	2436.865	98.102
5	4.235	3.075	0.328	9.043	0.364

300MHz 1HNMR P13 IN CDCL3

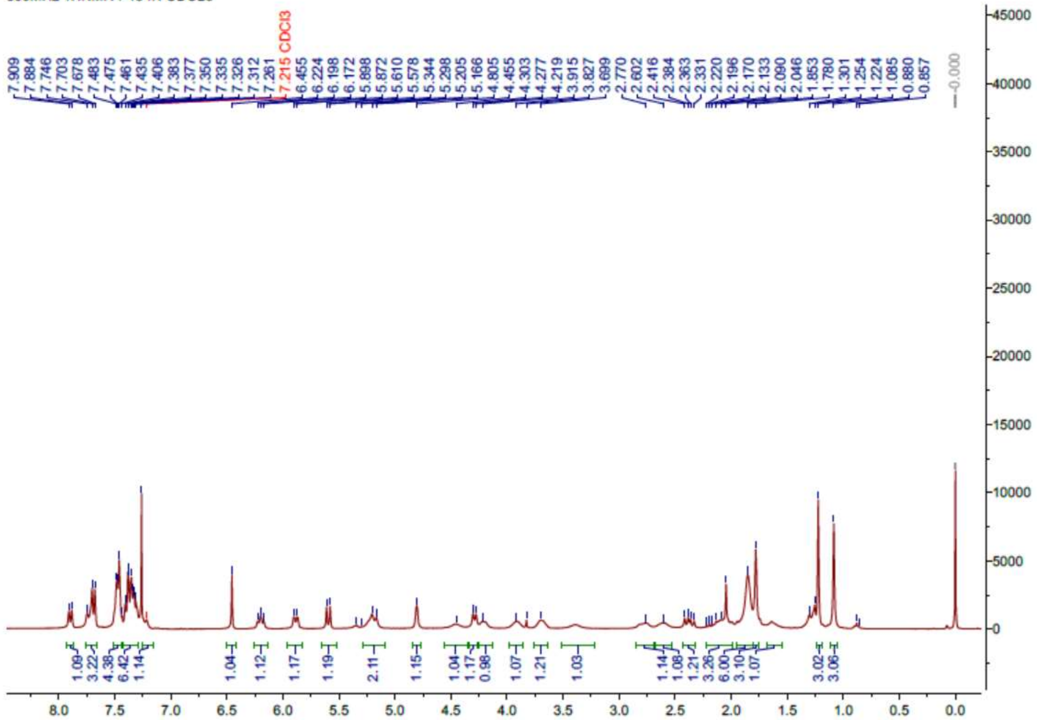

75MHz 13CNMR P13 IN CDCL3

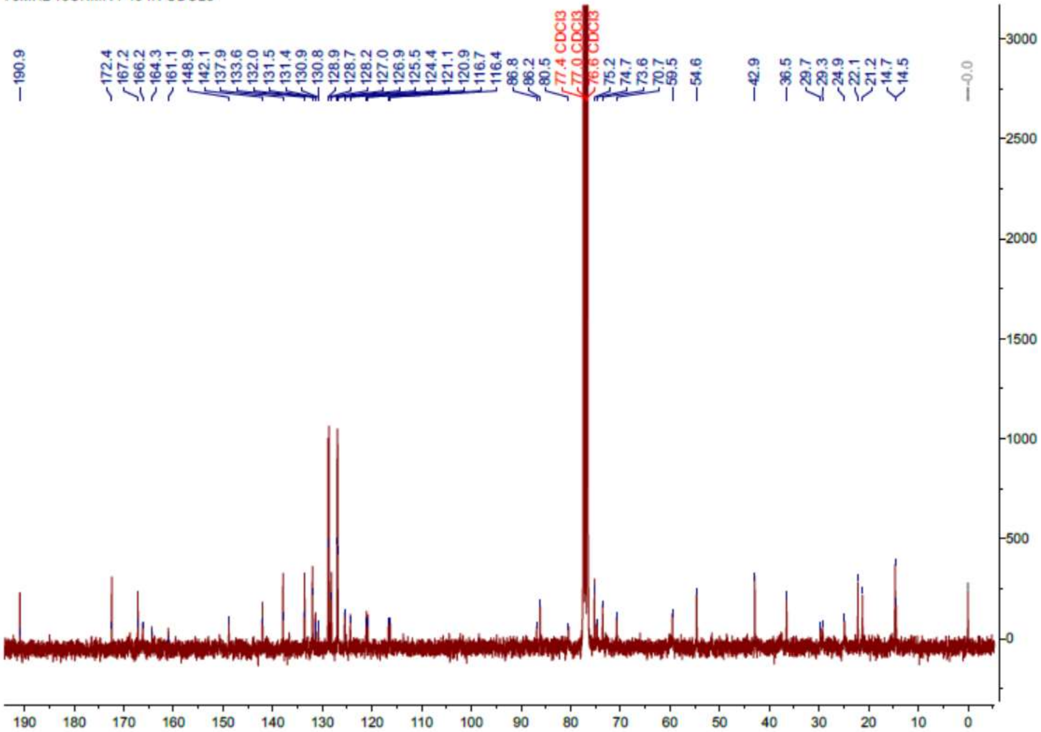

ESI-MS, HPLC, ^{1}H-NMR and ^{13}C-NMR spectra of compound **16d**

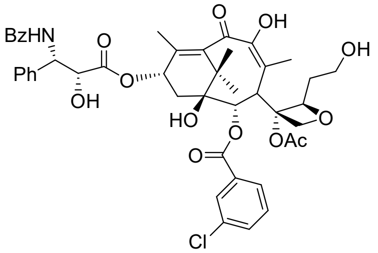

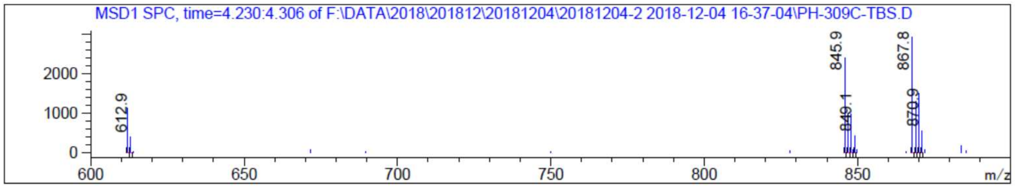

Sample Name : P12
Injection Date : Tue, 4. Dec. 2018 Seq. Line : 7
Inj. Volumn : 10.0 ul
Acq Operator :
Acq Method : F:\DATA\2018\201812\20181204\20181204-2 2018-12-04 16-37-04\MTH-GEN-MS.M
HPLC Analysis Conditions
1. Column : XDB C18 4.6*50mm 1.8um
2. Mobile Phase : A:Water+0.05%TFA B :ACN+0.05%TFA

	Time	A%	B%
3. Flow Mode :	0.00	90.0	10.0
	4.50	0.0	100.0
	6.50	0.0	100.0
	6.60	90.0	10.0

4. Flow : Start Flow : 1.0 ml/min

5. UV Wavelength: C: 220nm ;

6. Column Temp. : Left : 40.0C Right : 40.0C

7. Sample Preparation:

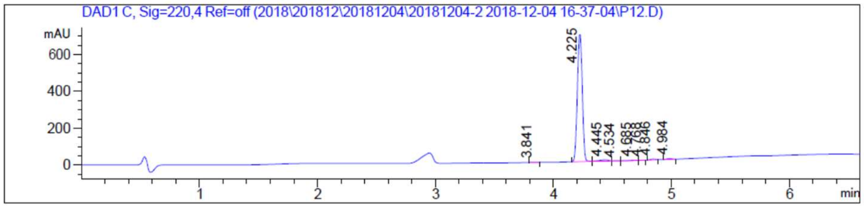

#	Meas. Ret. Time	Height	Height %	Area	Area %
1	3.841	1.481	0.206	3.904	0.188
2	4.225	698.170	97.032	1995.691	96.335
3	4.445	6.865	0.954	35.214	1.700
4	4.534	1.452	0.202	3.294	0.159
5	4.685	1.514	0.210	6.240	0.301
6	4.768	0.318	0.044	0.831	0.040
7	4.846	4.569	0.635	12.370	0.597
8	4.984	5.155	0.716	14.065	0.679

400MHz 1HNMR P12 IN CDCL3

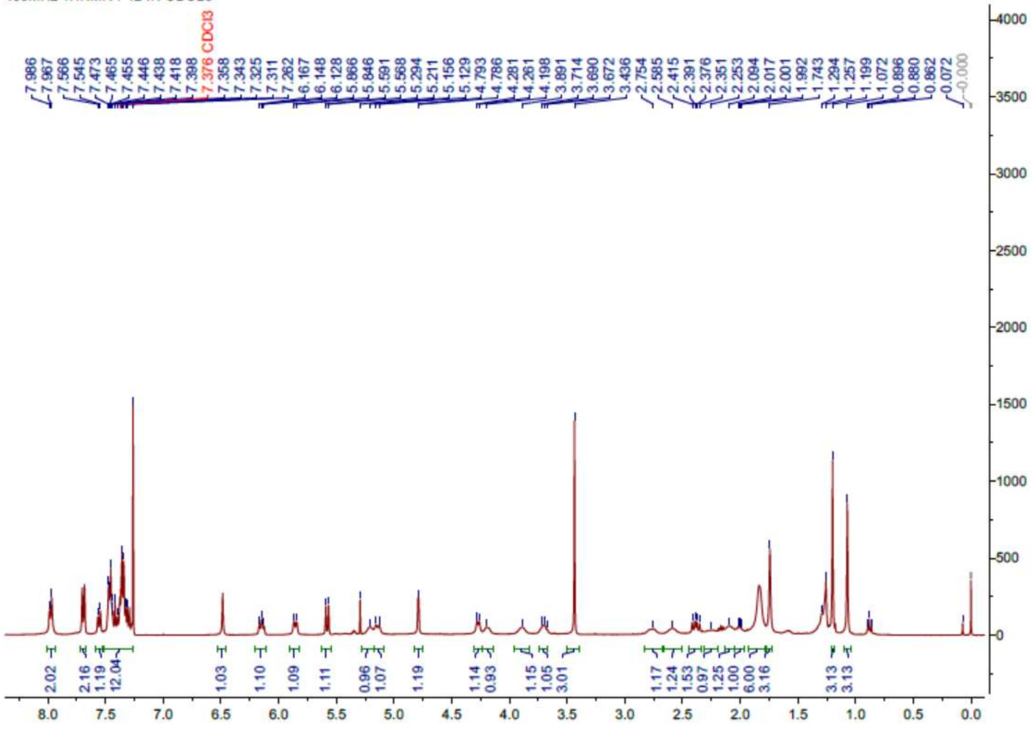

100MHz 13CNMR P12 IN CDCL3

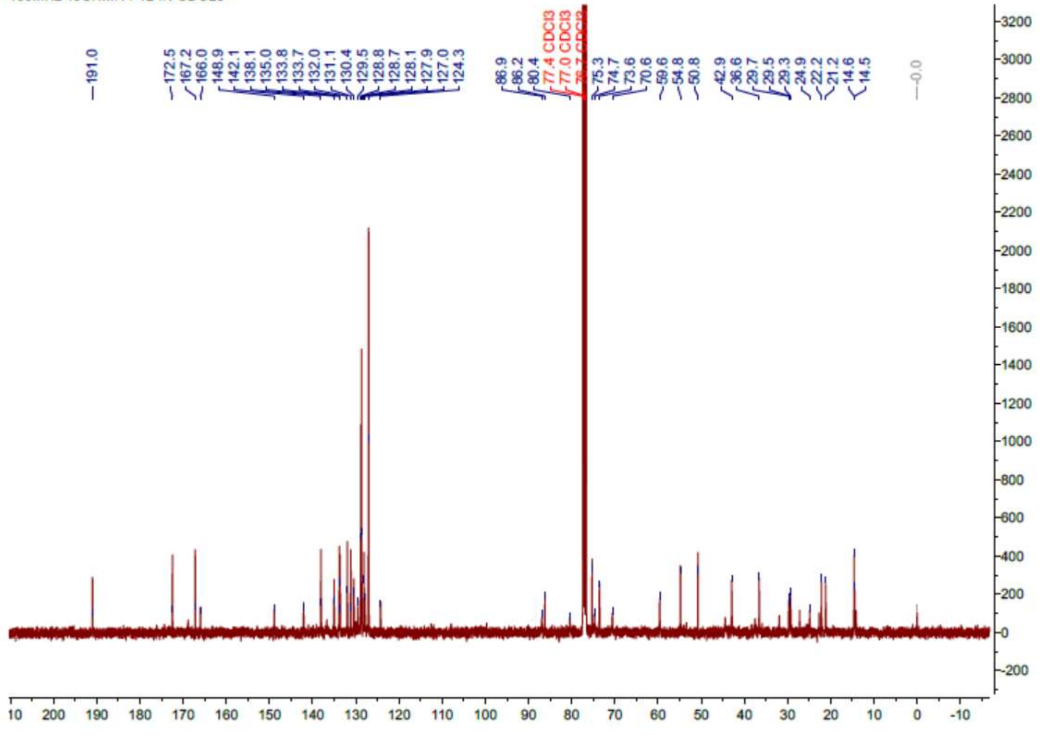

ESI-MS, HPLC, ^{1}H-NMR and ^{13}C-NMR spectra of compound **16e**

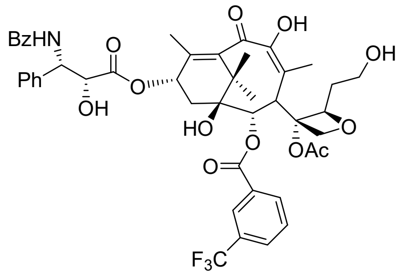

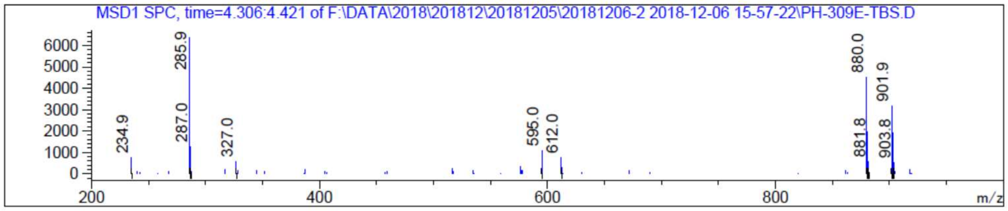


```
Sample Name       : P14
Injection Date    : Thu, 6. Dec. 2018                        Seq. Line :   2
Inj. Volumn       : 15.0 ul
Acq Operator      :
Acq   Method      : F:\DATA\2018\201812\20181205\20181206-2 2018-12-06 15-57-22\MTH-GEN-
                    MS.M
                    HPLC Analysis Conditions
1. Column        : XDB C18  4.6*50mm  1.8um
2. Mobile Phase :  A:Water+0.05%TFA     B :ACN+0.05%TFA
                   Time              A%                    B%
3. Flow Mode     : 0.00              90.0                  10.0
                   4.50              0.0                   100.0
                   6.50              0.0                   100.0
                   6.60              90.0                  10.0
 4. Flow         :   Start Flow :  1.0  ml/min

 5. UV Wavelength:   C: 220nm ;

 6. Column Temp. :   Left :    40.0C    Right :     40.0C

 7. Sample Preparation:
```

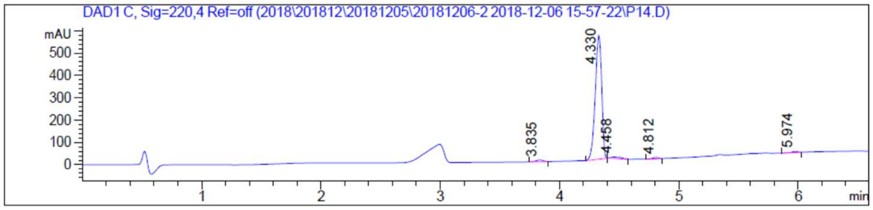

#	Meas. Ret. Time	Height	Height %	Area	Area %
1	3.835	7.757	1.334	29.258	1.341
2	4.330	555.961	95.580	2075.914	95.136
3	4.458	5.782	0.994	39.019	1.788
4	4.812	6.368	1.095	20.925	0.959
5	5.974	5.801	0.997	16.933	0.776

400MHz 1HNMR P14 IN CDCL3

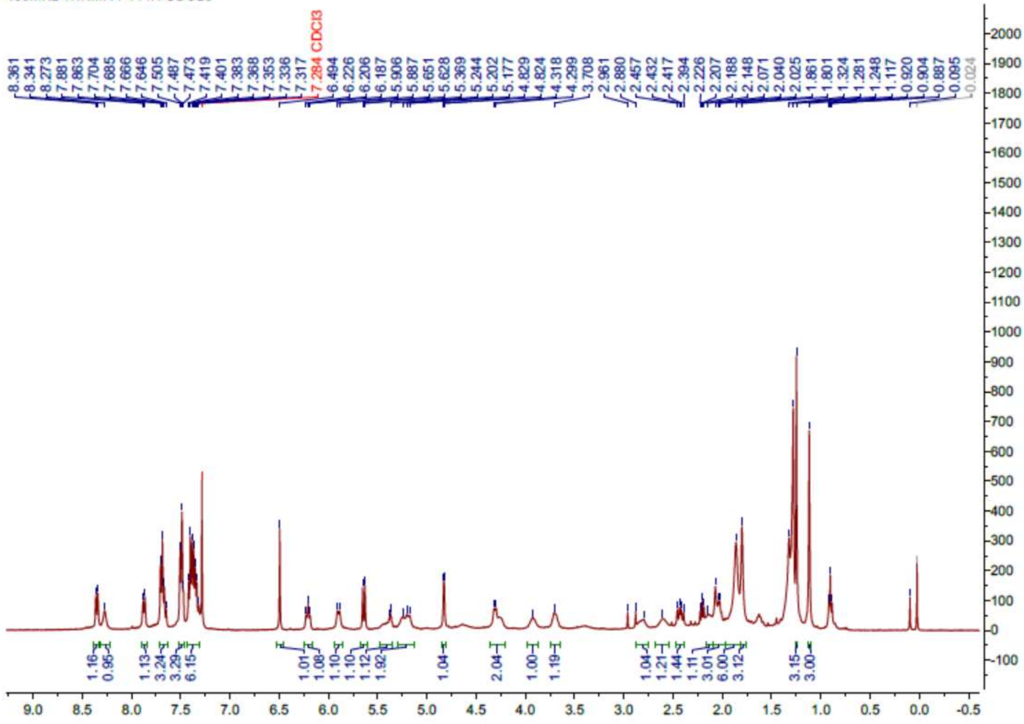

100MHz 13CNMR P14 IN CDCL3

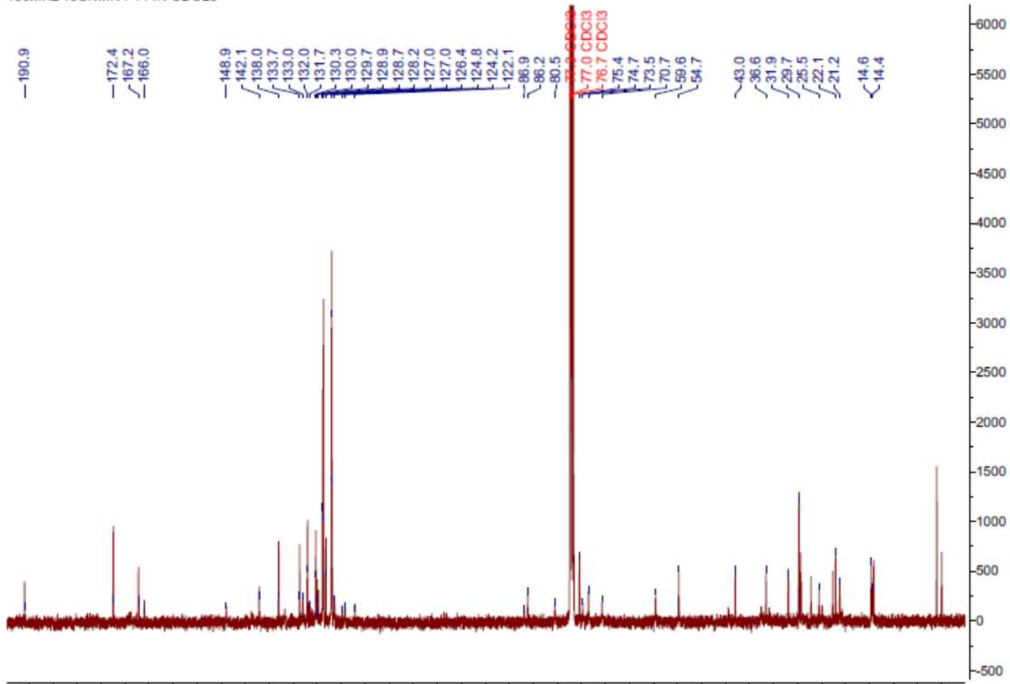

ESI-MS, ^{1}H-NMR and ^{13}C-NMR spectra of compound **17a**

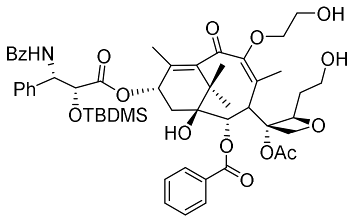

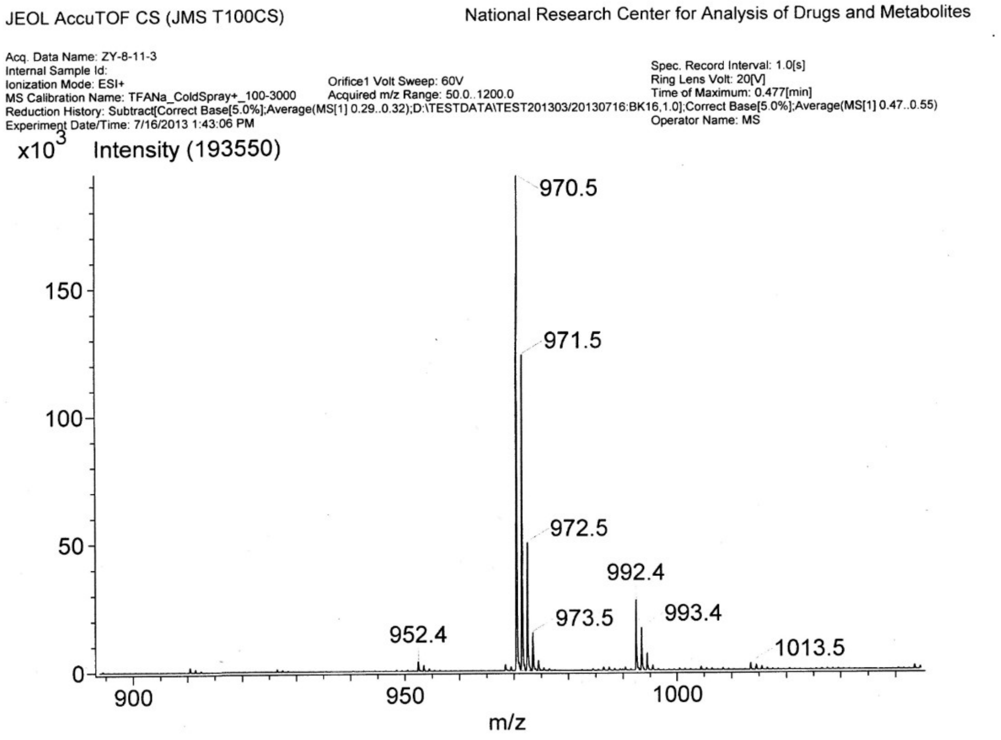

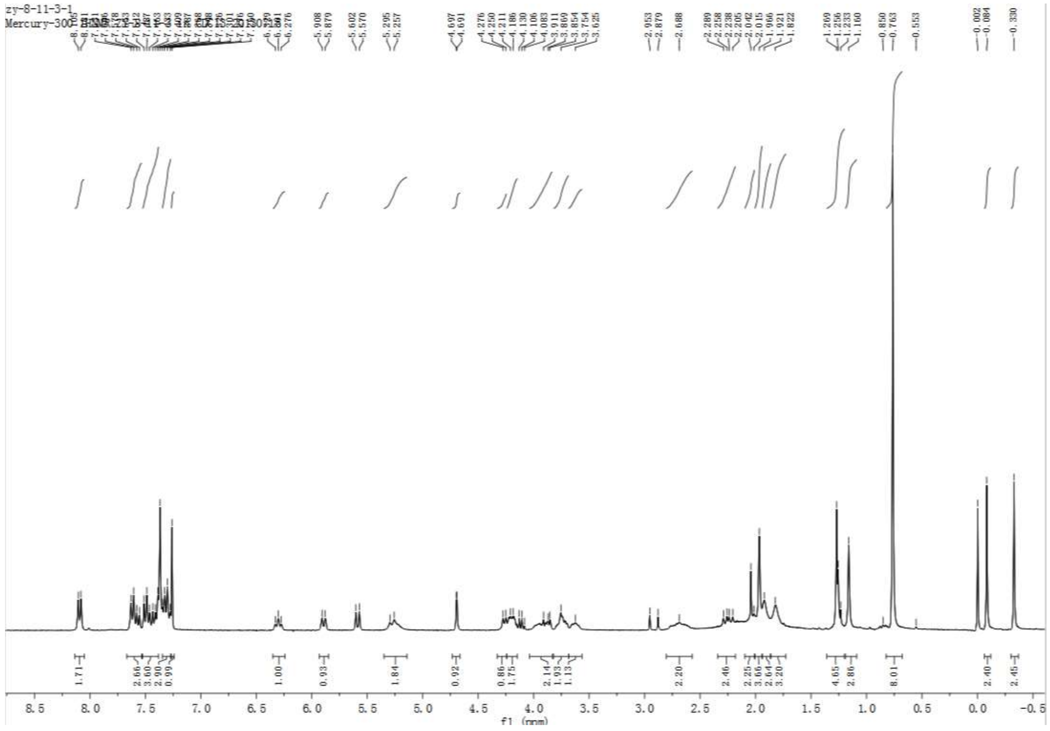

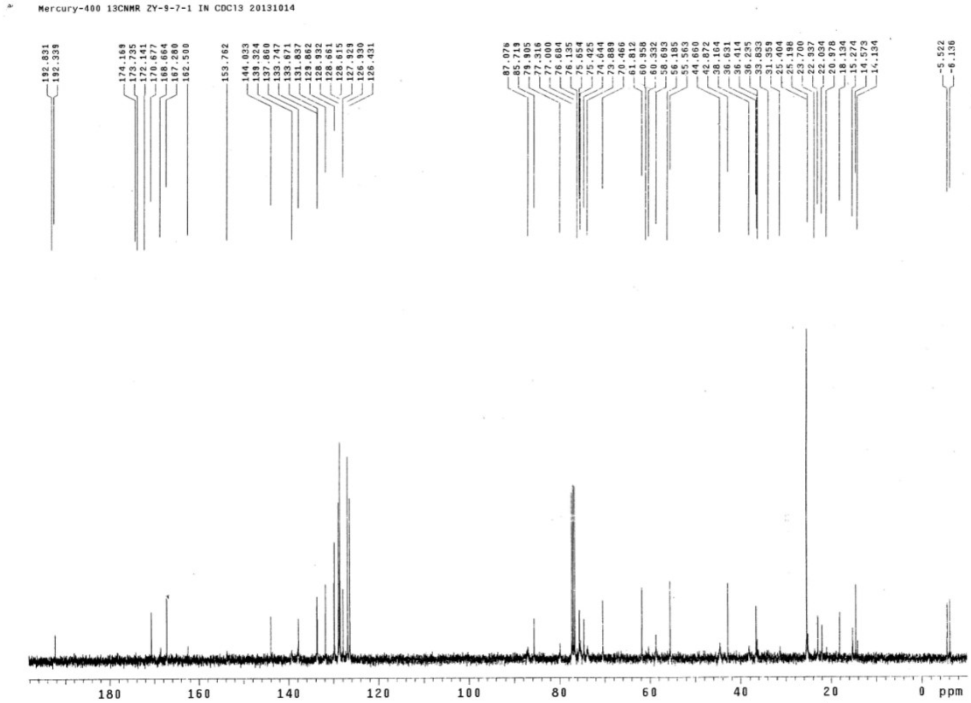
Mercury-400 13CNMR ZY-8-7-1 IN CDCl3 20131014

ESI-MS, ^{1}H-NMR and ^{13}C-NMR spectra of compound **17b**

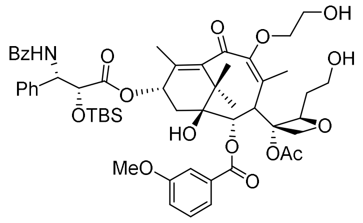

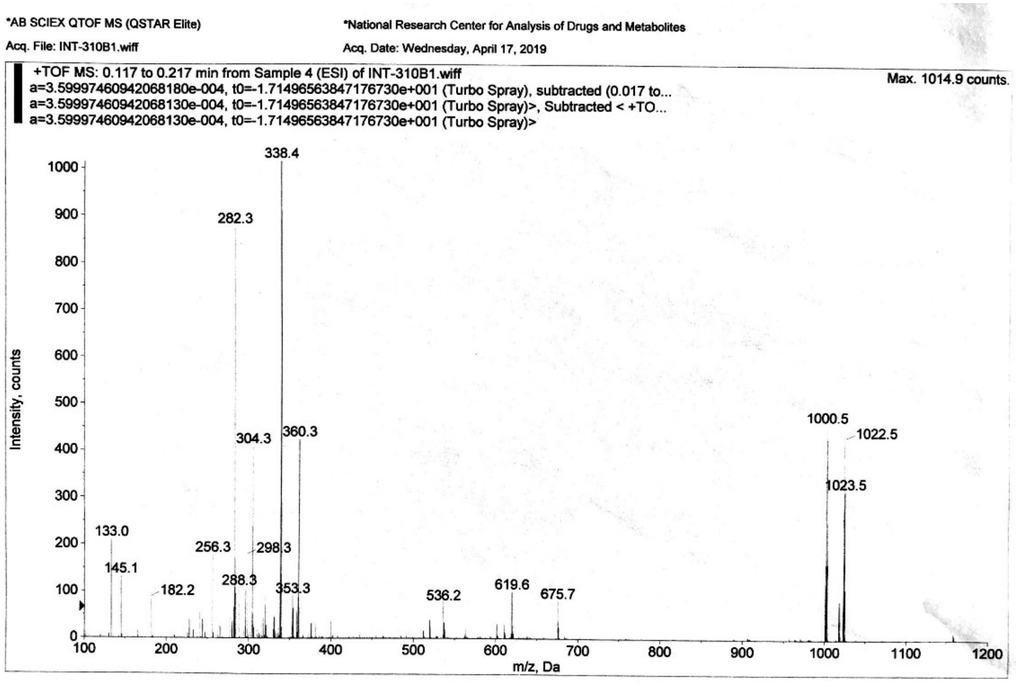

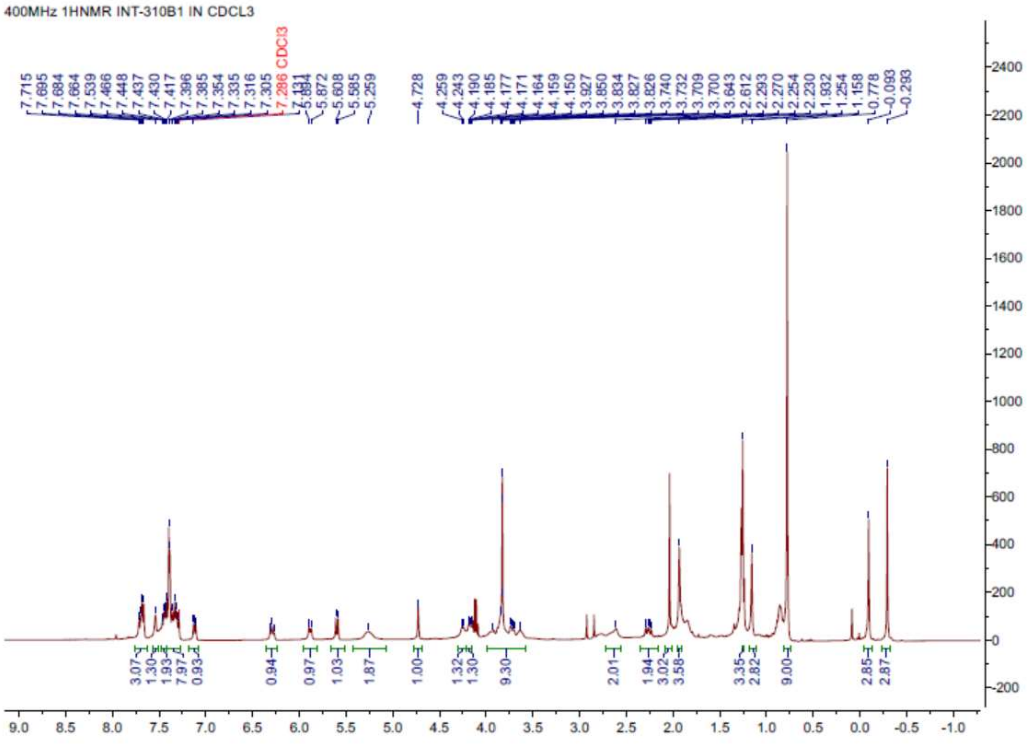

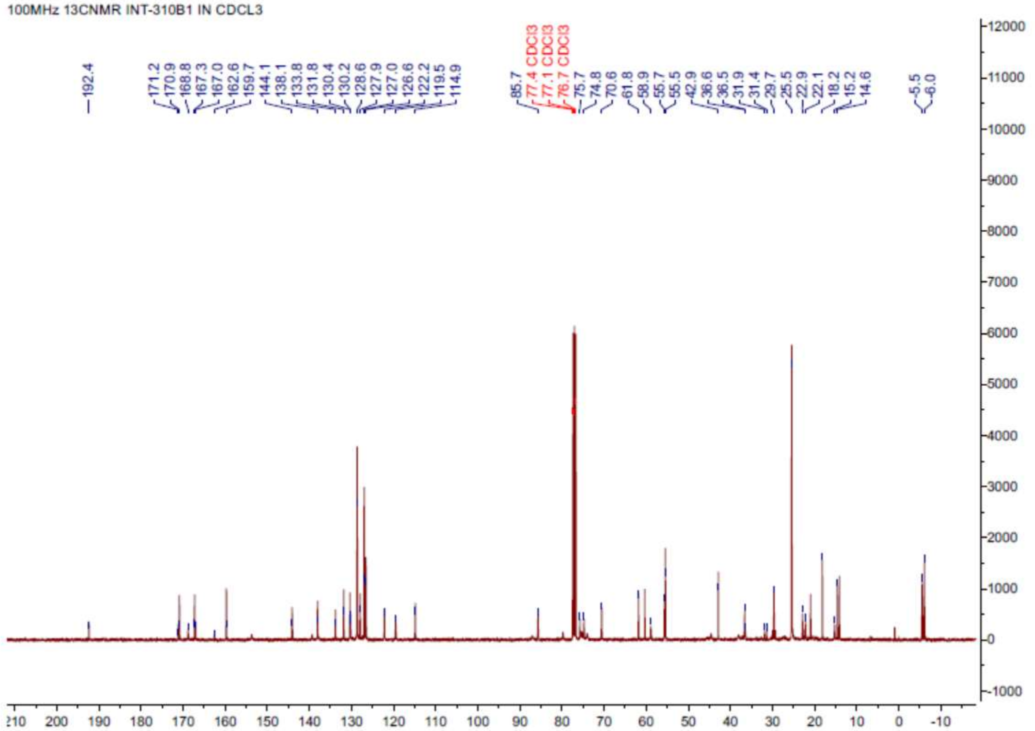
100MHz 13CNMR INT-310B1 IN CDCL3
192.4
171.2
170.9
168.8
167.3
167.0
162.6
159.7
144.1
138.1
133.8
131.8
130.4
130.2
128.6
127.9
127.0
126.6
122.2
119.5
114.9
85.7
77.4 CDCl3
77.1 CDCl3
76.7 CDCl3
75.7
74.8
70.6
61.8
58.9
55.7
55.5
42.9
36.6
36.5
31.9
31.4
29.7
25.5
22.9
22.1
18.2
15.2
14.6
-5.5
-6.0
12000
11000
10000
9000
8000
7000
6000
5000
4000
3000
2000
1000
0
-1000
210 200 190 180 170 160 150 140 130 120 110 100 90 80 70 60 50 40 30 20 10 0 -10

ESI-MS, ^{1}H-NMR and ^{13}C-NMR spectra of compound **17c**

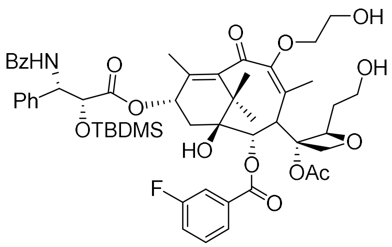

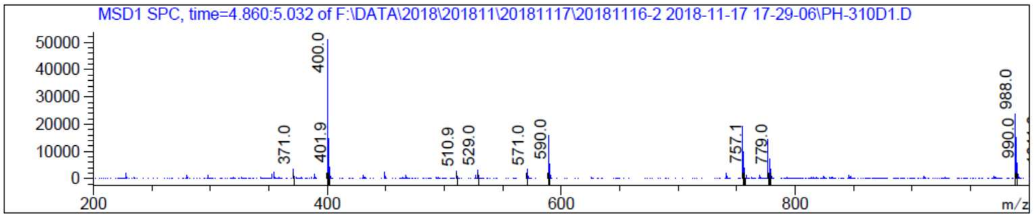

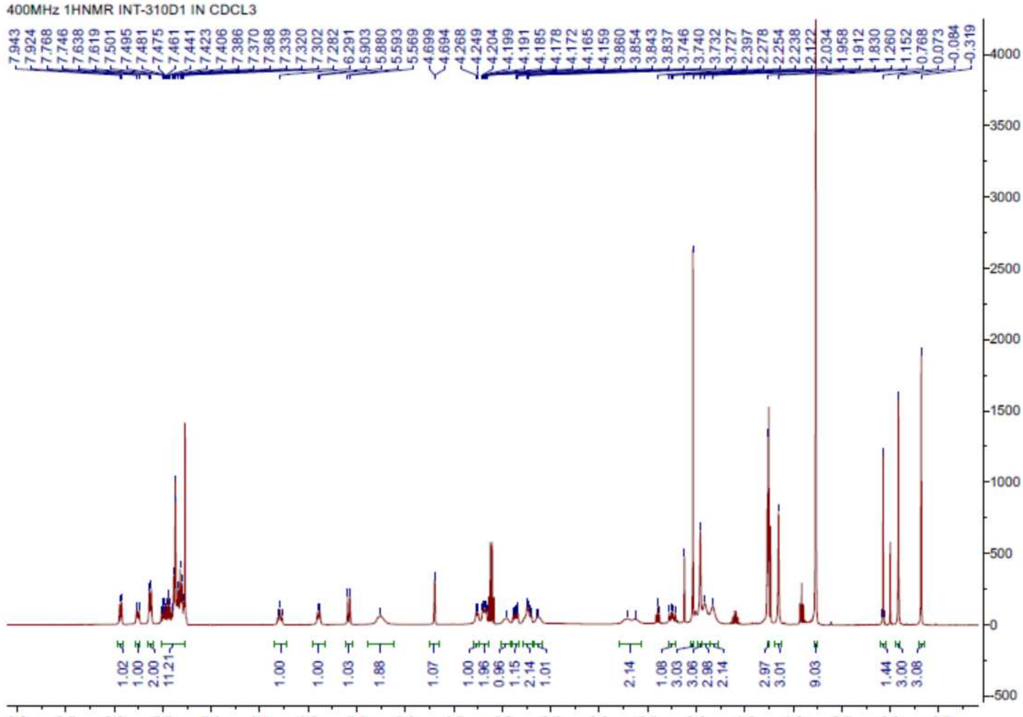

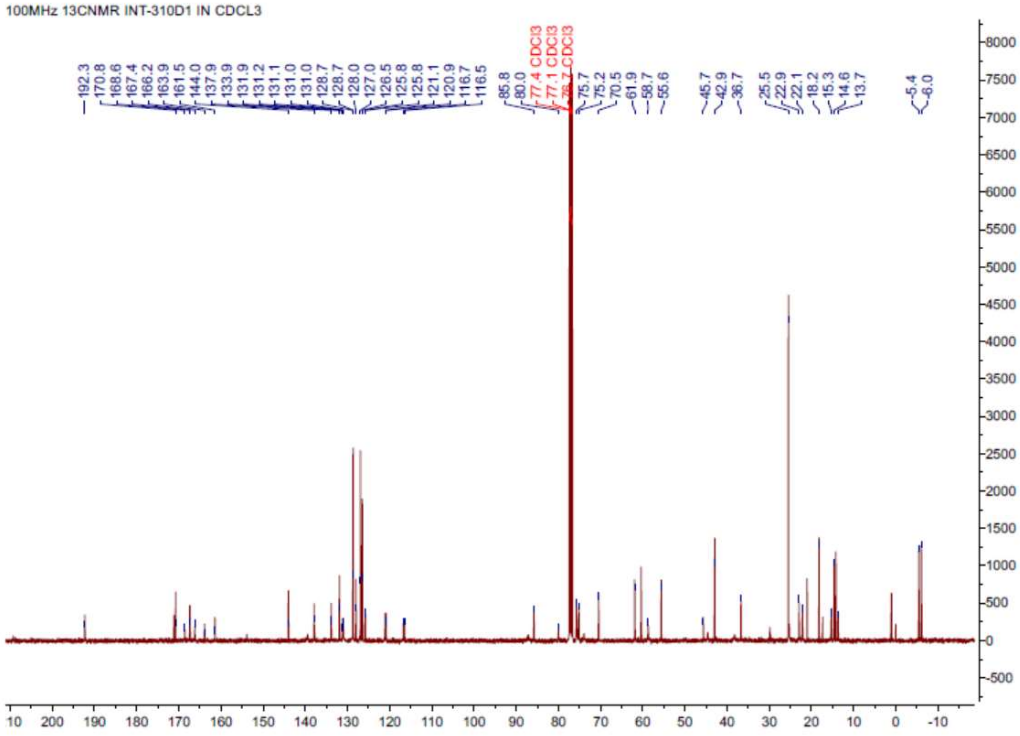
100MHz 13CNMR INT-310D1 IN CDCL3

ESI-MS, ^{1}H-NMR and ^{13}C-NMR spectra of compound **17d**

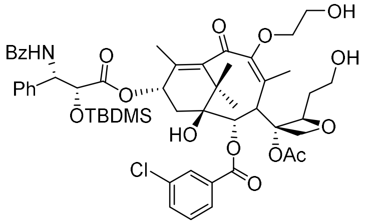

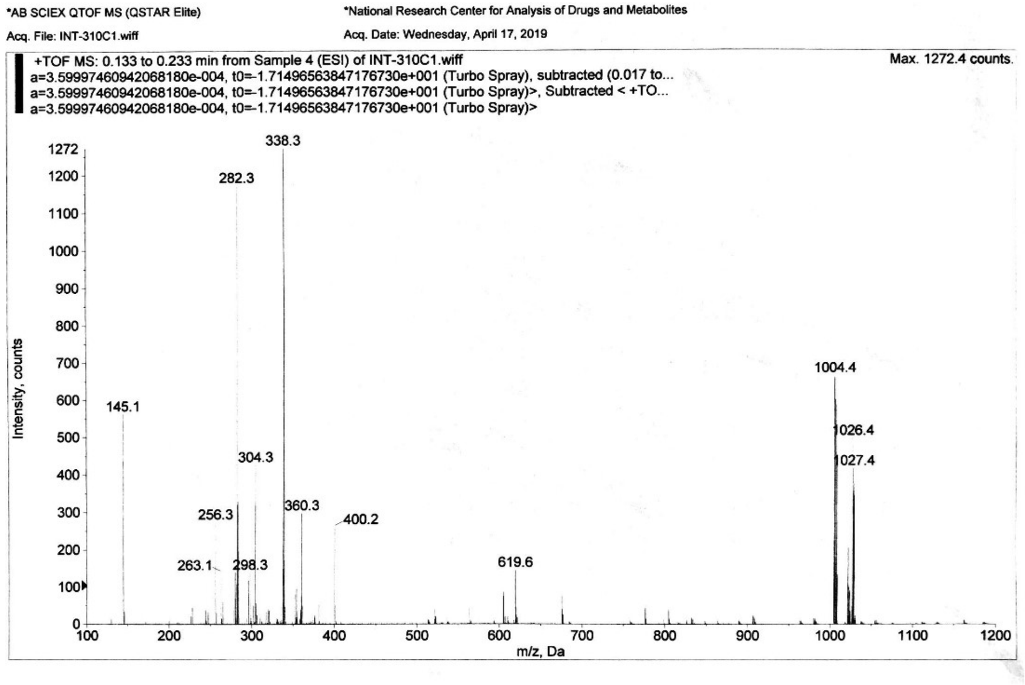

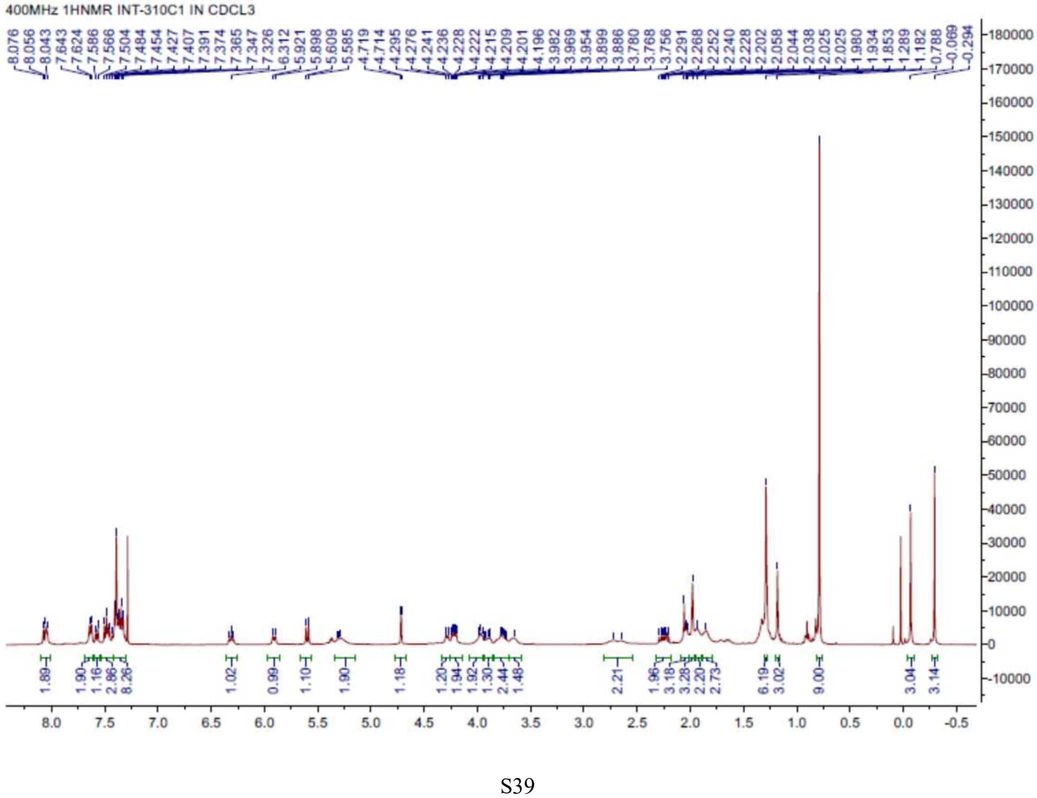

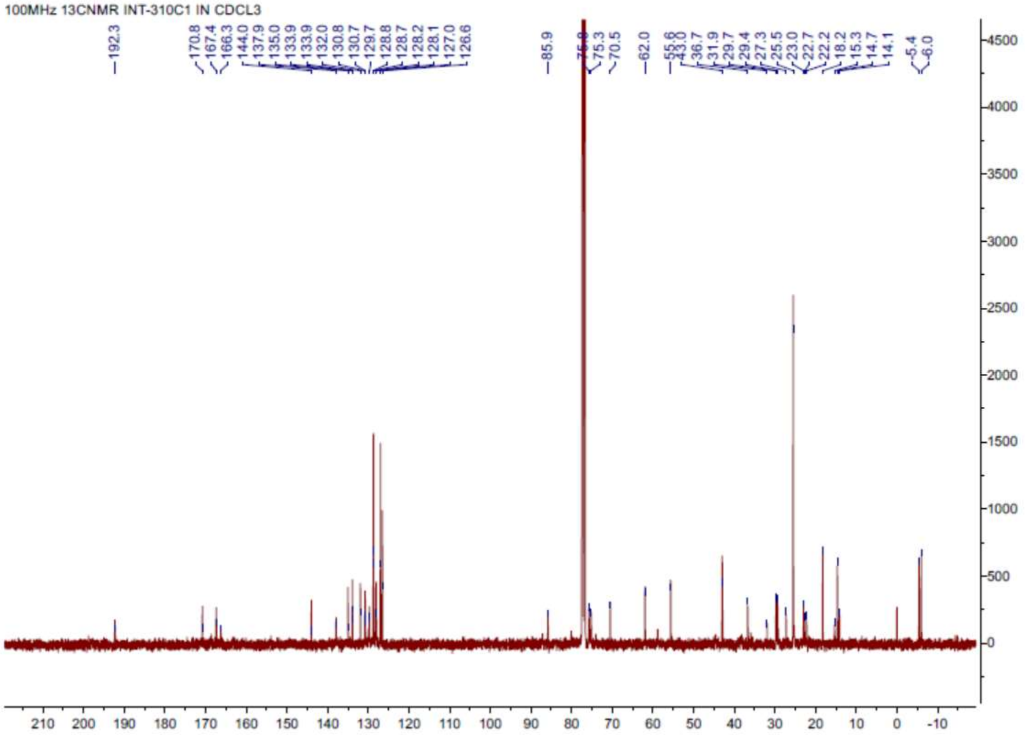
100MHz 13CNMR INT-310C1 IN CDCL3

ESI-MS, ^{1}H-NMR and ^{13}C-NMR spectra of compound **17e**

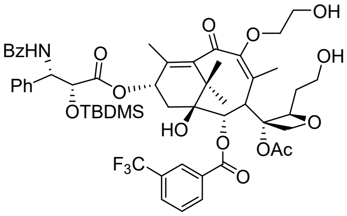

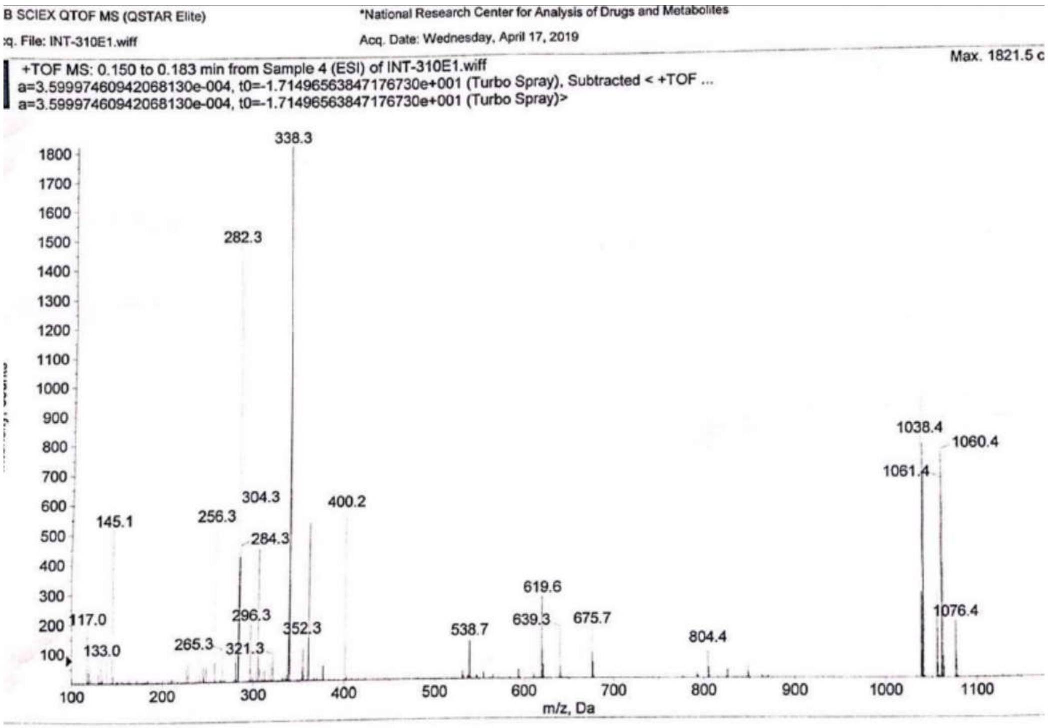

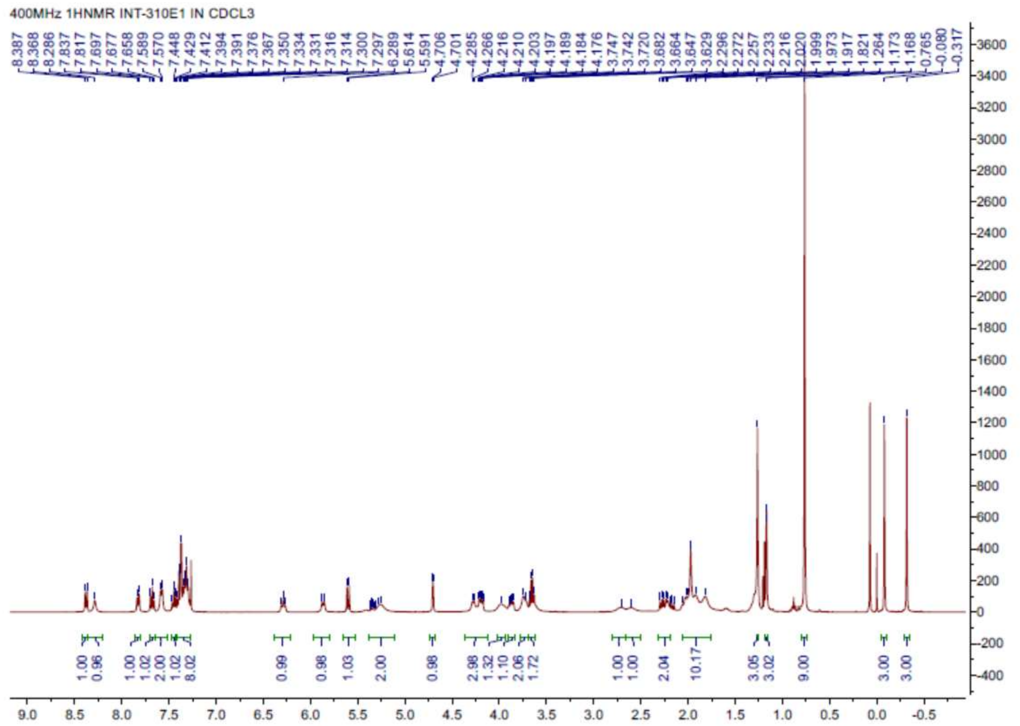
400MHz 1HNMR INT-310E1 IN CDCL3

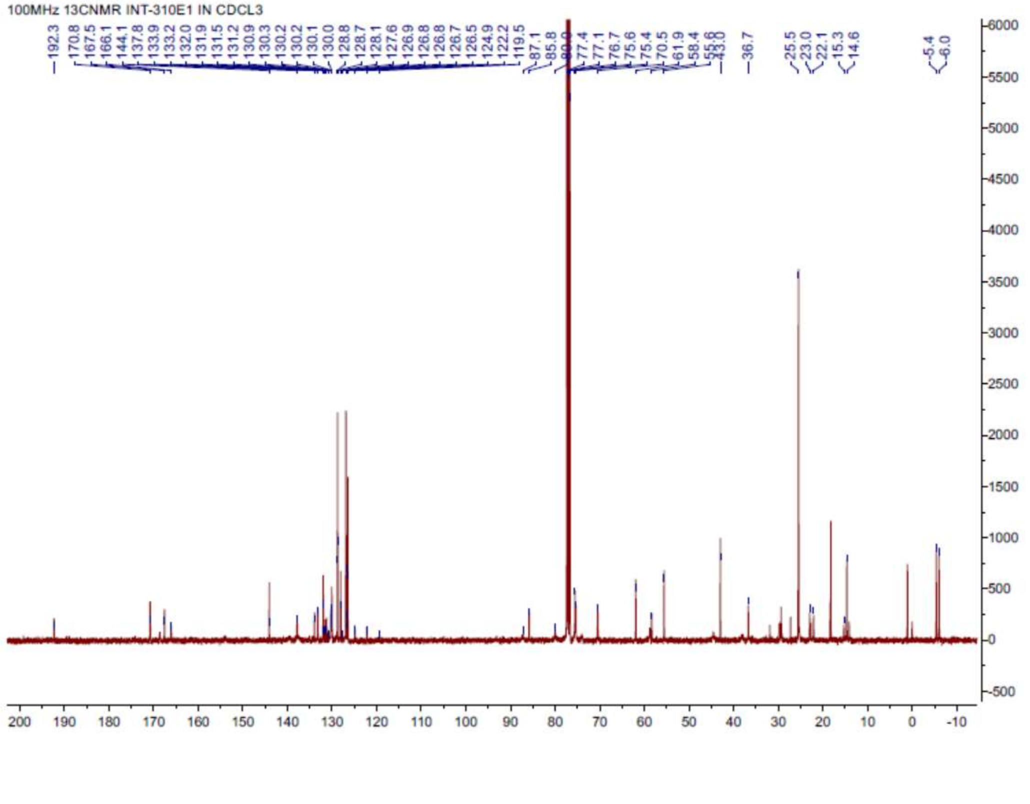
100MHz 13CNMR INT-310E1 IN CDCL3

ESI-MS, ^{1}H-NMR and ^{13}C-NMR spectra of compound **18a**

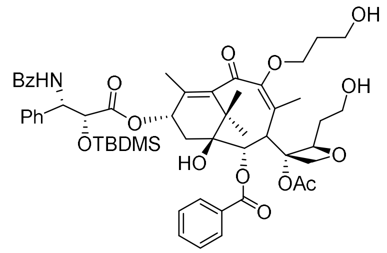

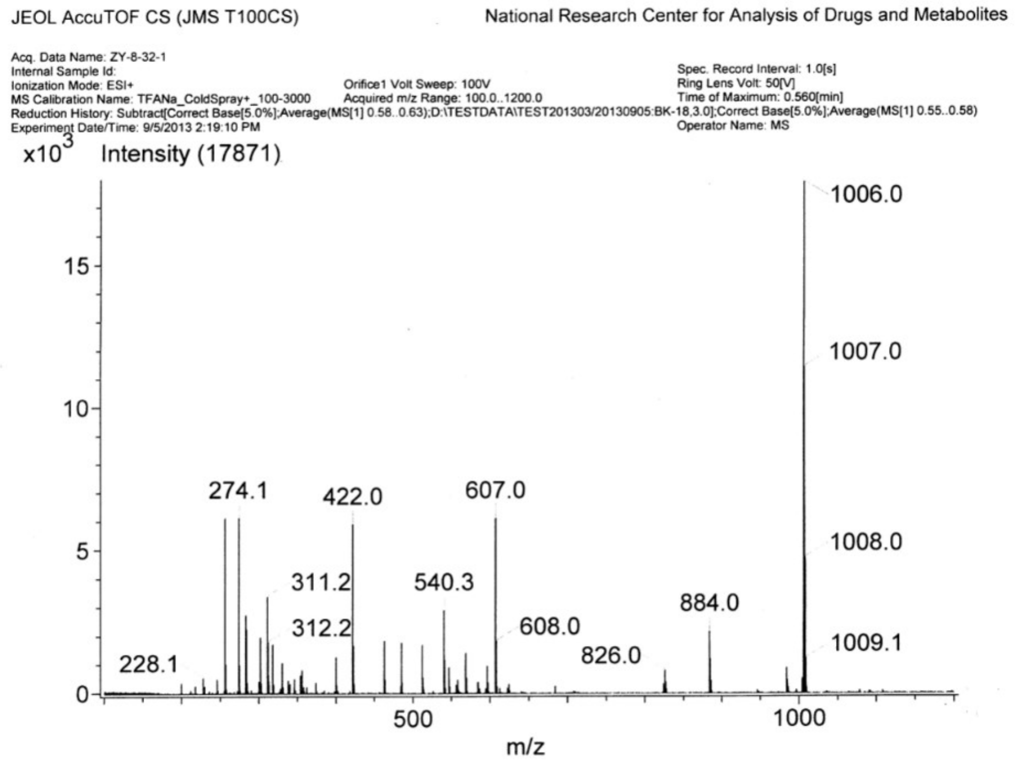

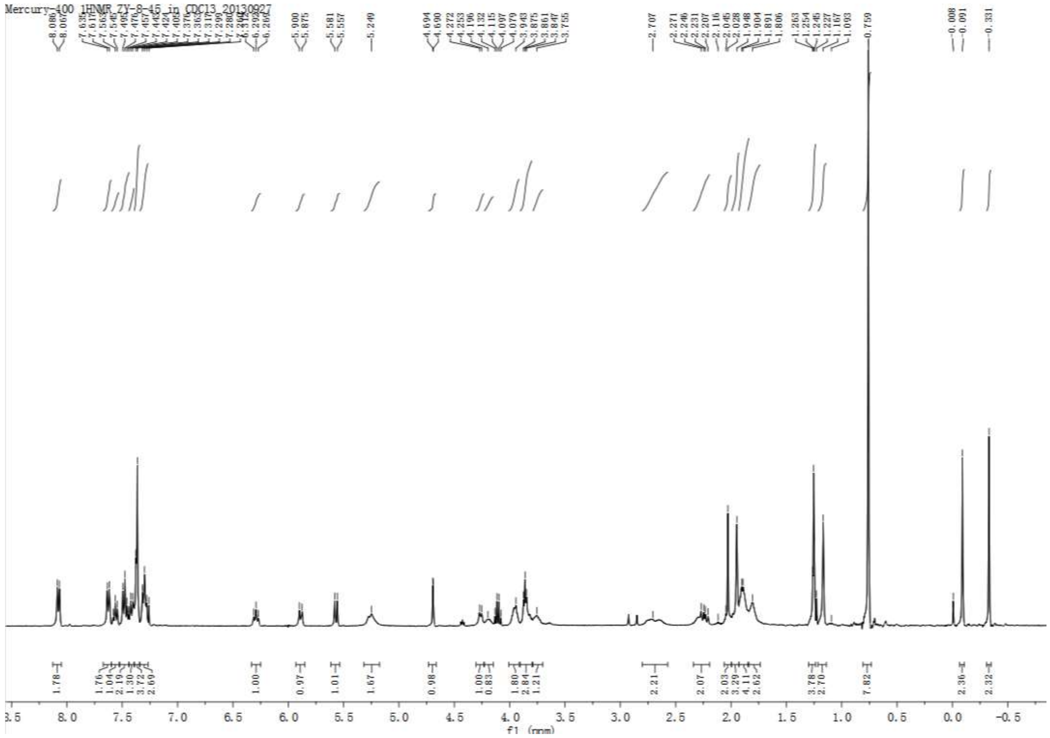
Mercury-400 1HNMR ZY-8-45 in CDCl3 20130927
f1 (ppm)

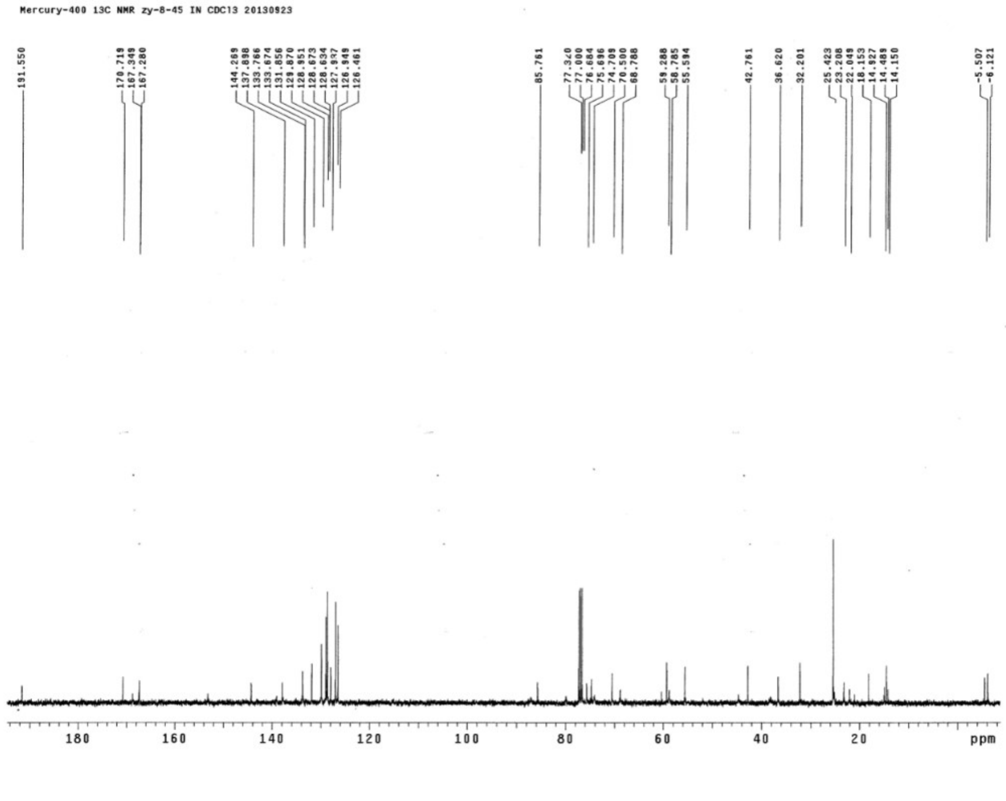
Mercury-400 13C NMR zy-8-45 IN CDCl3 20130923
ppm

ESI-MS, ^{1}H-NMR and ^{13}C-NMR spectra of compound **18b**

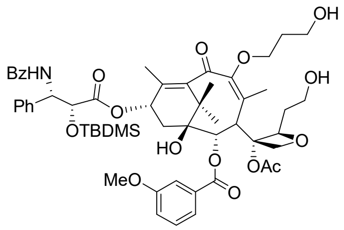

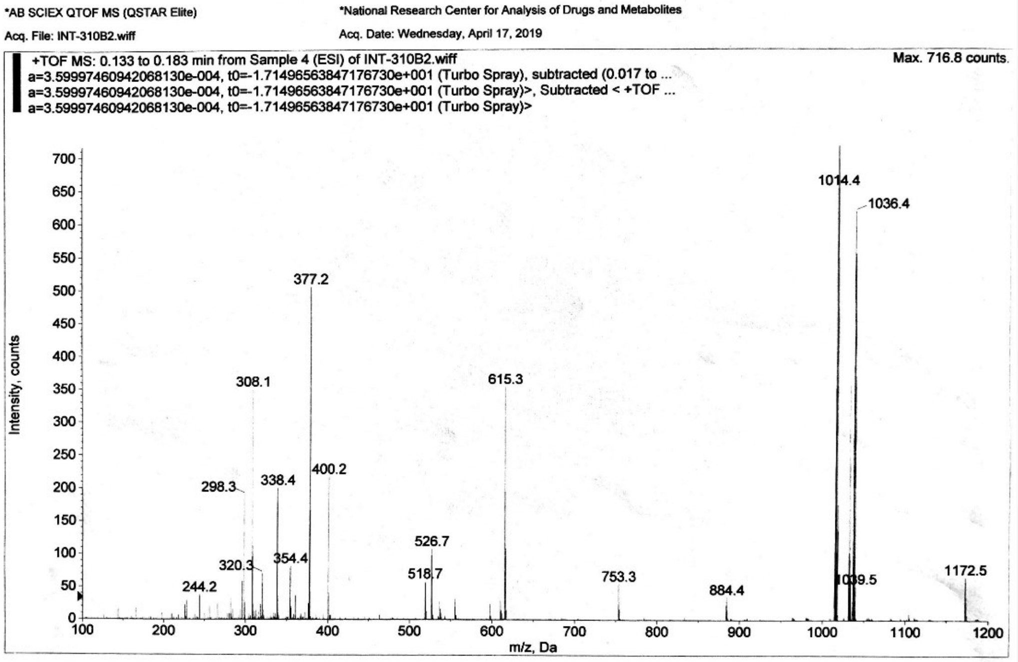

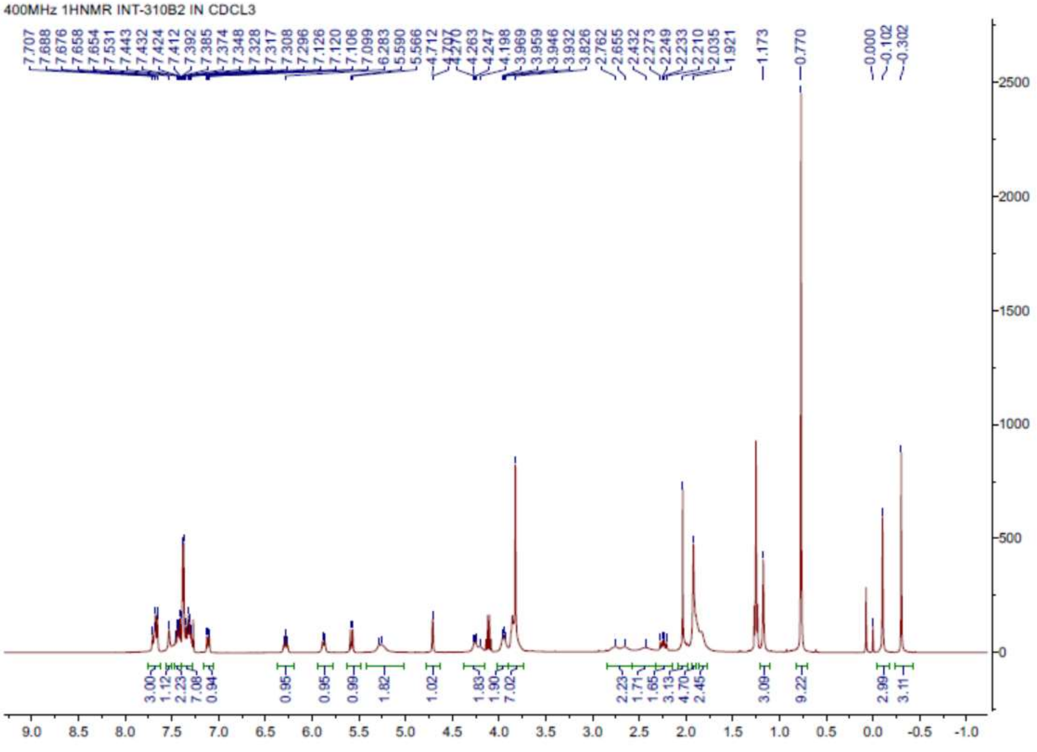

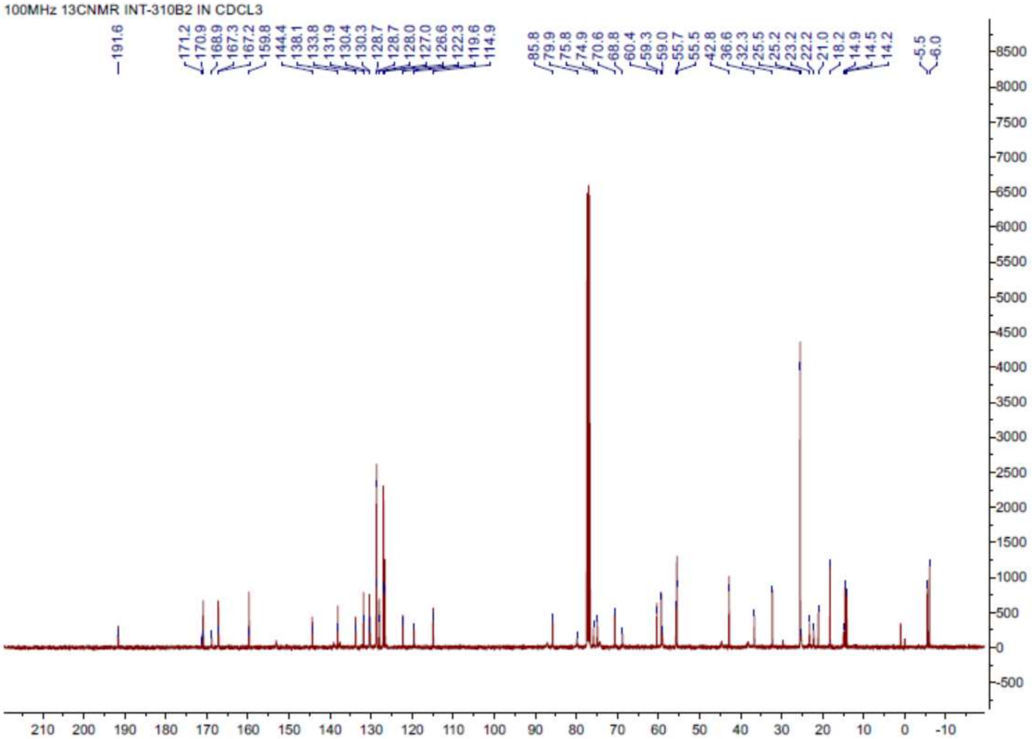

100MHz 13CNMR INT-310B2 IN CDCL3
191.6
171.2
170.9
168.9
167.3
167.2
159.8
144.4
138.1
133.8
131.9
130.4
130.3
128.7
128.7
128.0
127.0
126.6
122.3
119.6
114.9
85.8
79.9
75.8
74.9
70.6
68.8
60.4
59.3
59.0
55.7
55.5
42.8
36.6
32.3
25.5
25.2
23.2
22.2
21.0
18.2
14.9
14.5
14.2
-5.5
-6.0

ESI-MS, ^{1}H-NMR and ^{13}C-NMR spectra of compound **18c**

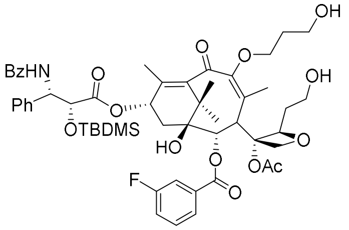

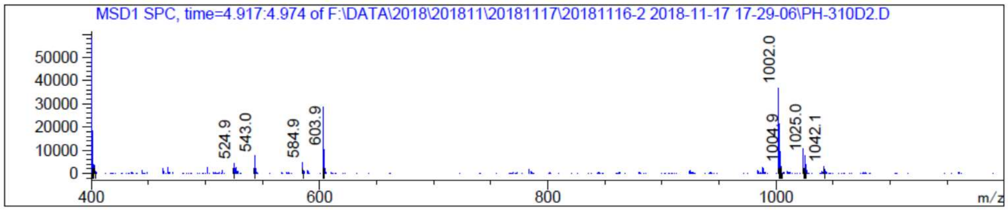

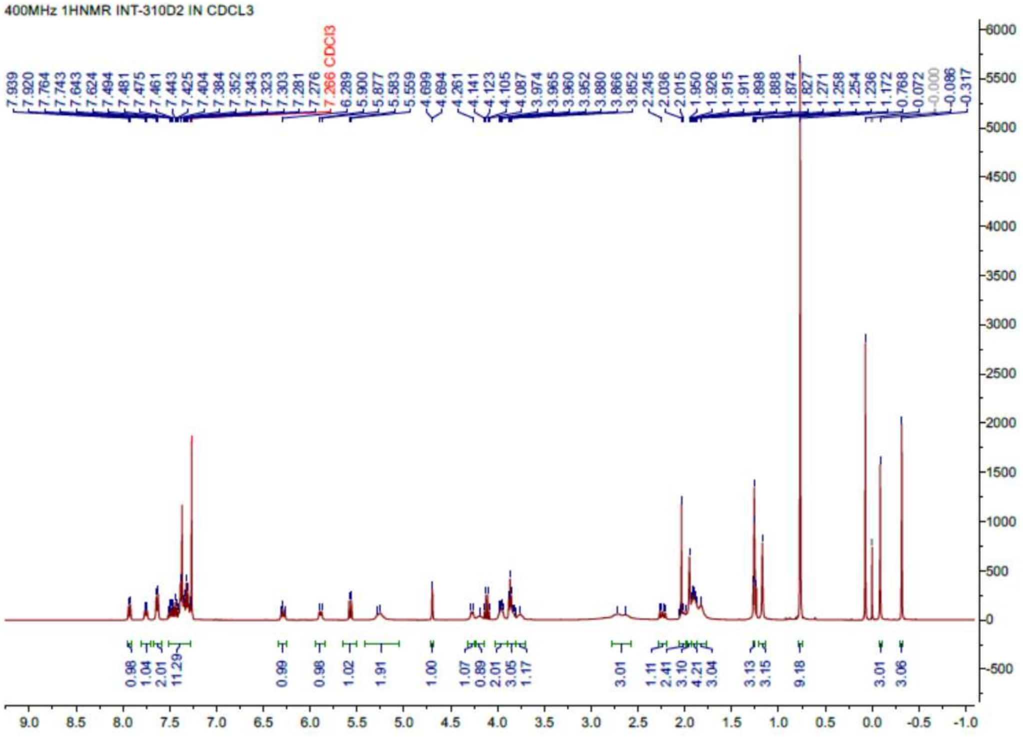

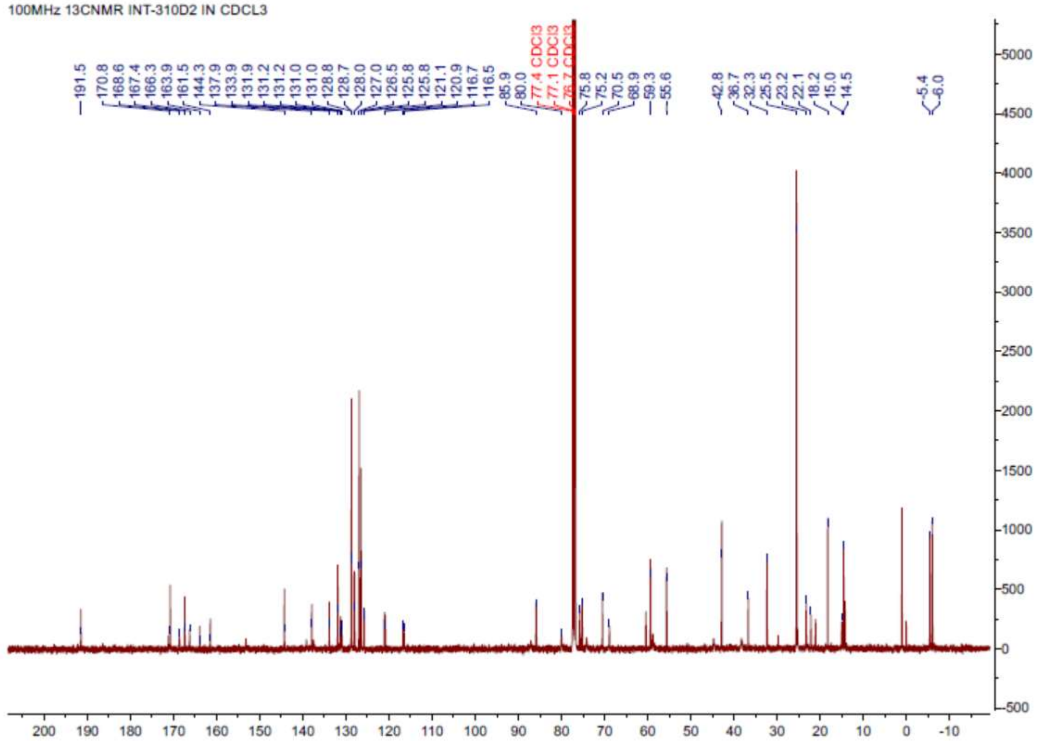
100MHz 13CNMR INT-310D2 IN CDCL3
191.5
170.8
168.6
167.4
166.3
163.9
161.5
144.3
137.9
133.9
131.9
131.2
131.2
131.0
131.0
128.8
128.7
128.0
127.0
126.5
125.8
125.8
121.1
120.9
116.7
116.5
85.9
80.0
77.4 CDCl3
77.1 CDCl3
76.7 CDCl3
75.8
75.2
70.5
68.9
59.3
55.6
42.8
36.7
32.3
25.5
23.2
22.1
18.2
15.0
14.5
-5.4
-6.0

ESI-MS, ^{1}H-NMR and ^{13}C-NMR spectra of compound **18d**

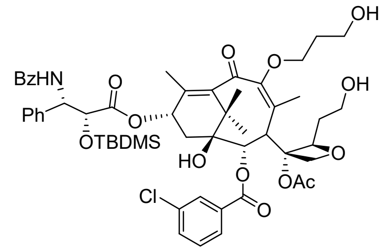

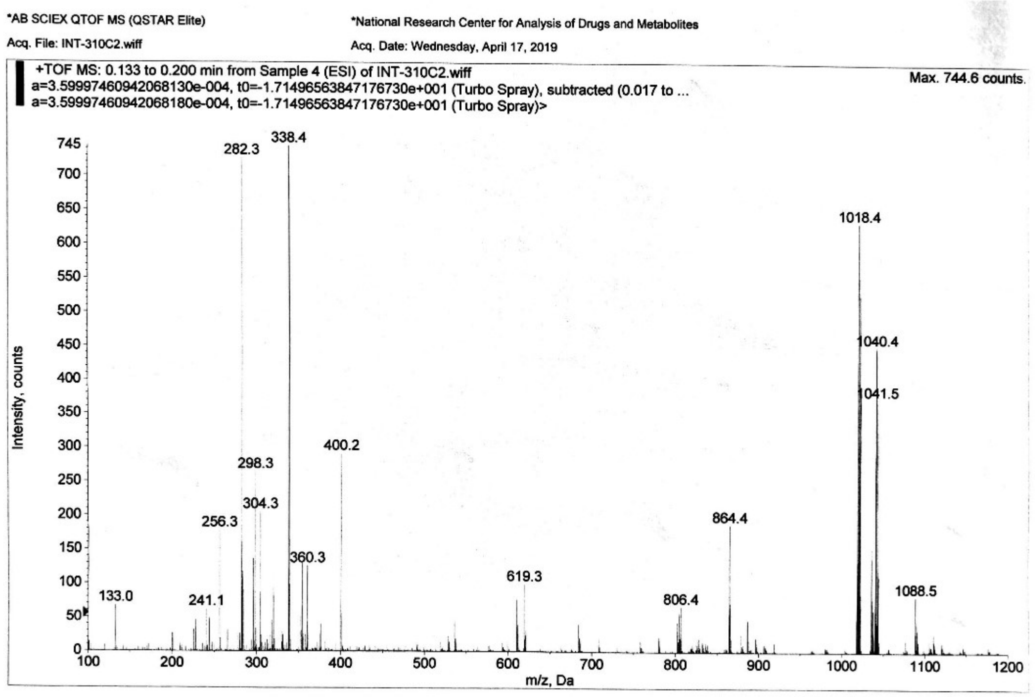

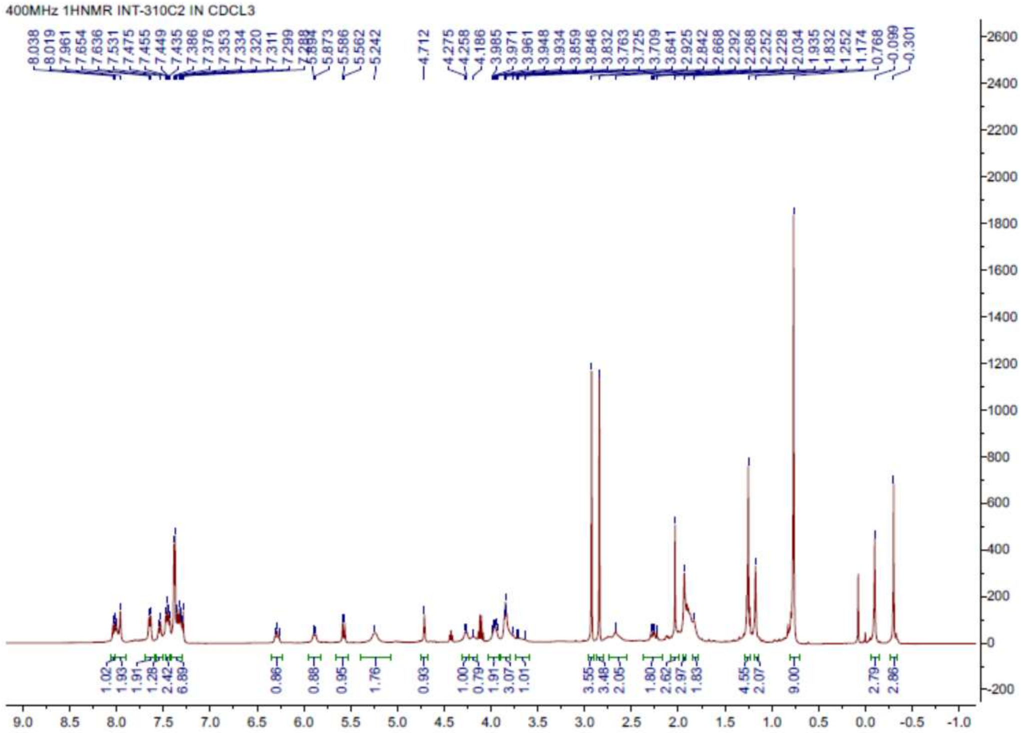
400MHz 1HNMR INT-310C2 IN CDCL3

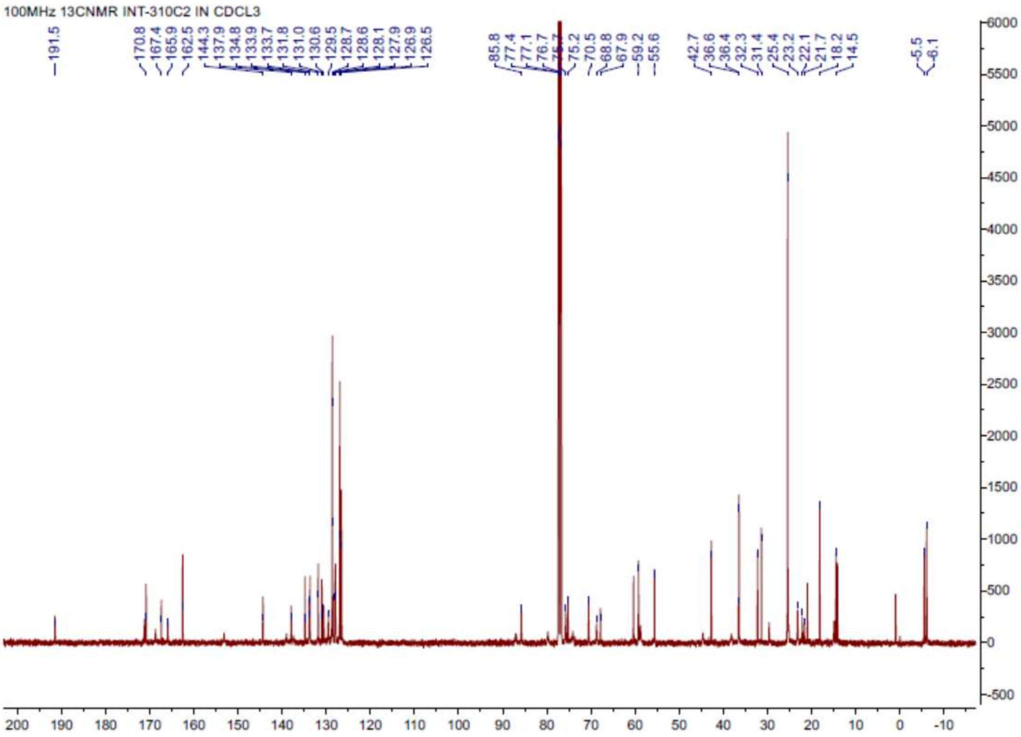
100MHz 13CNMR INT-310C2 IN CDCL3

ESI-MS, ^{1}H-NMR and ^{13}C-NMR spectra of compound **18e**

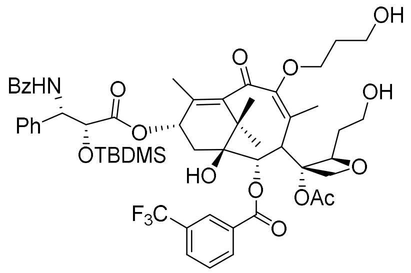

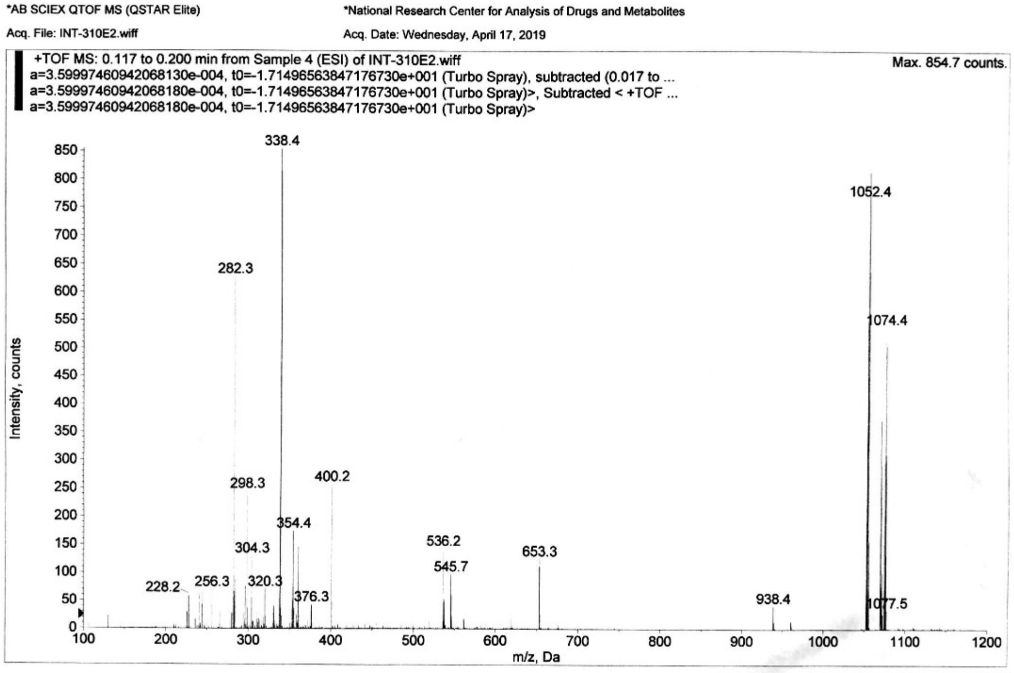

400MHz 1HNMR INT-310E2 IN CDCL3

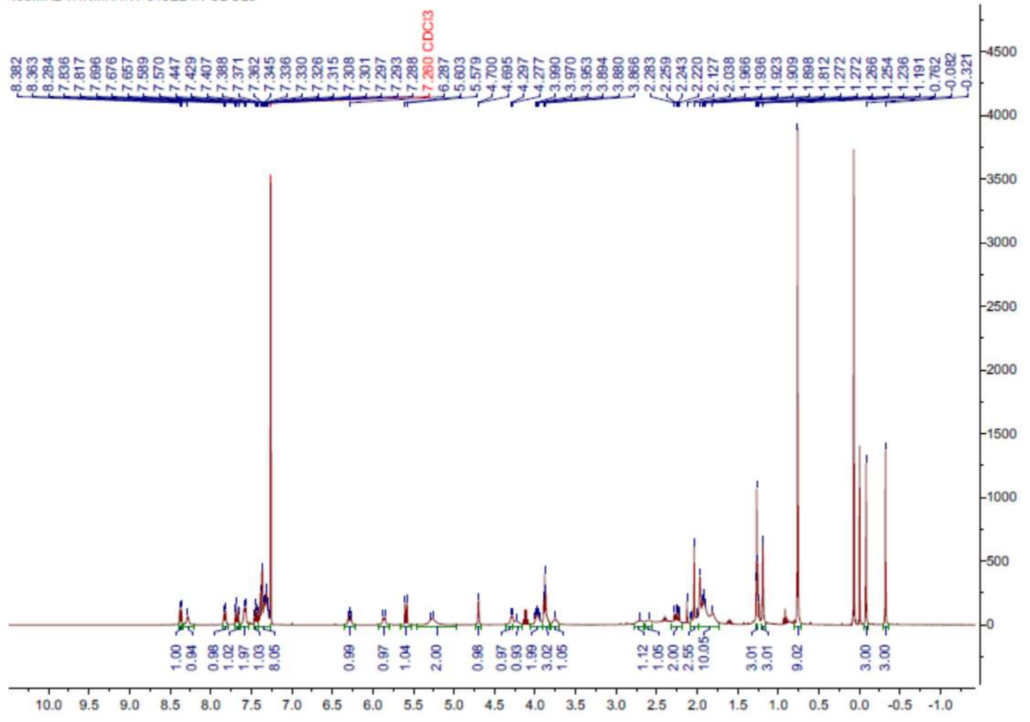

100MHz 13CNMR INT-310E2 IN CDCL3

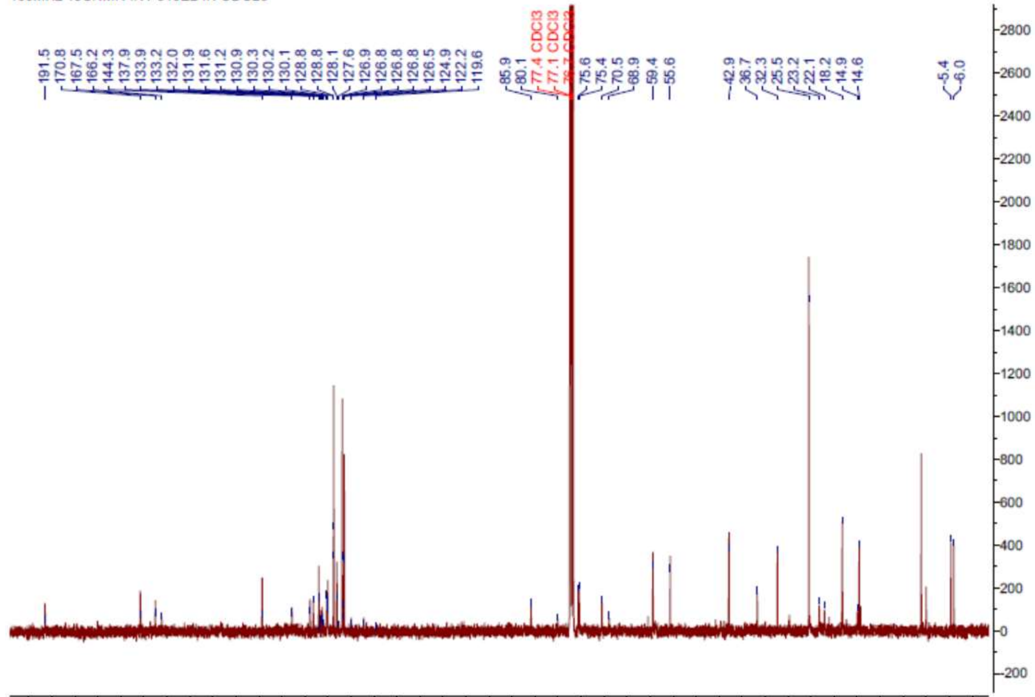

ESI-MS, ^{1}H-NMR and ^{13}C-NMR spectra of compound **19a**

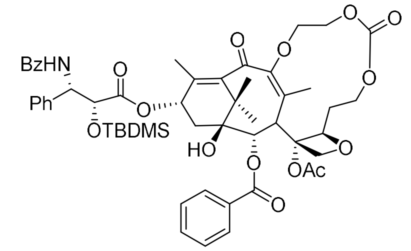

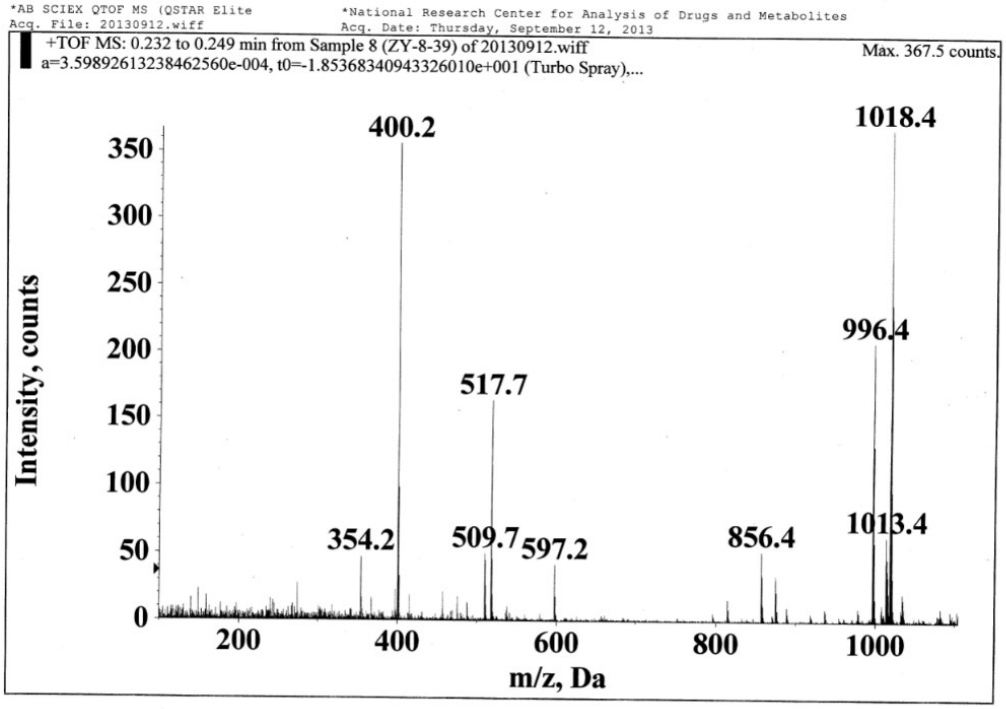

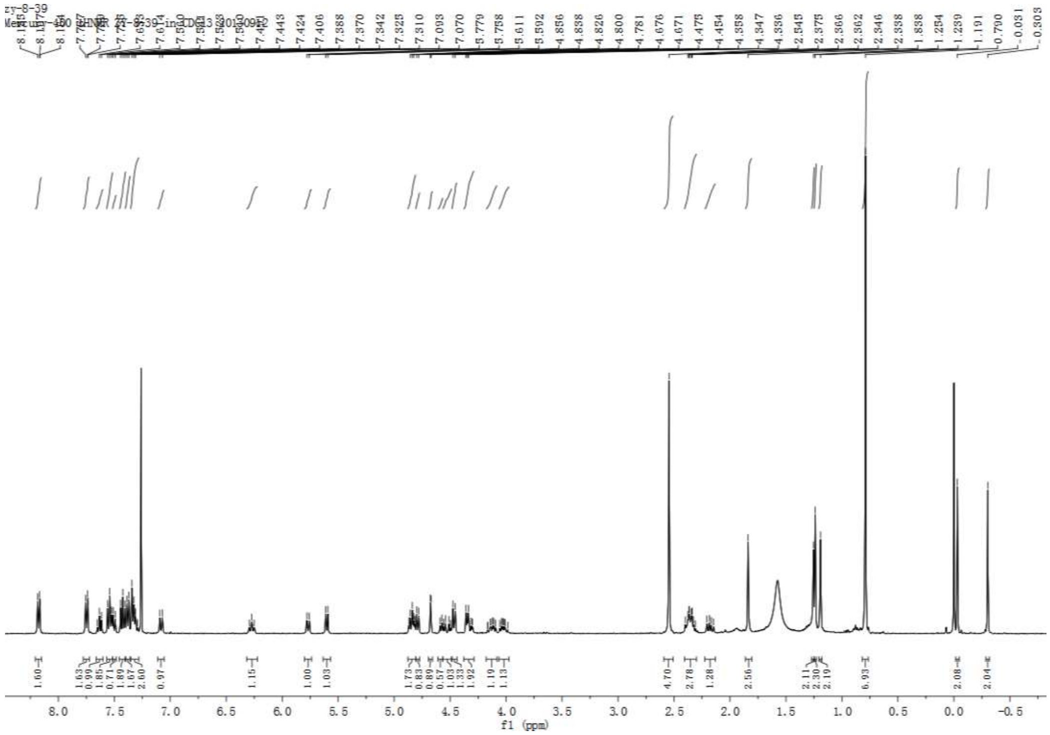
zy-8-39
Mercury-400 1HNMR ZY-8-39 in CDCl3 20130912
7.443
7.424
7.406
7.388
7.370
7.342
7.325
7.310
7.093
7.070
5.779
5.758
5.611
5.592
4.856
4.838
4.826
4.800
4.781
4.676
4.671
4.475
4.454
4.358
4.347
4.336
2.545
2.375
2.366
2.362
2.346
2.338
1.838
1.254
1.239
1.191
0.790
-0.031
-0.303
1.60
1.63
0.99
1.85
0.71
1.89
1.67
2.60
0.97
1.15
1.00
1.03
1.73
0.83
0.89
0.57
1.03
1.33
1.92
1.19
1.13
4.70
2.78
1.28
2.56
2.11
2.30
2.19
6.93
2.08
2.04
8.0
7.5
7.0
6.5
6.0
5.5
5.0
4.5
4.0
3.5
3.0
2.5
2.0
1.5
1.0
0.5
0.0
-0.5
f1 (ppm)

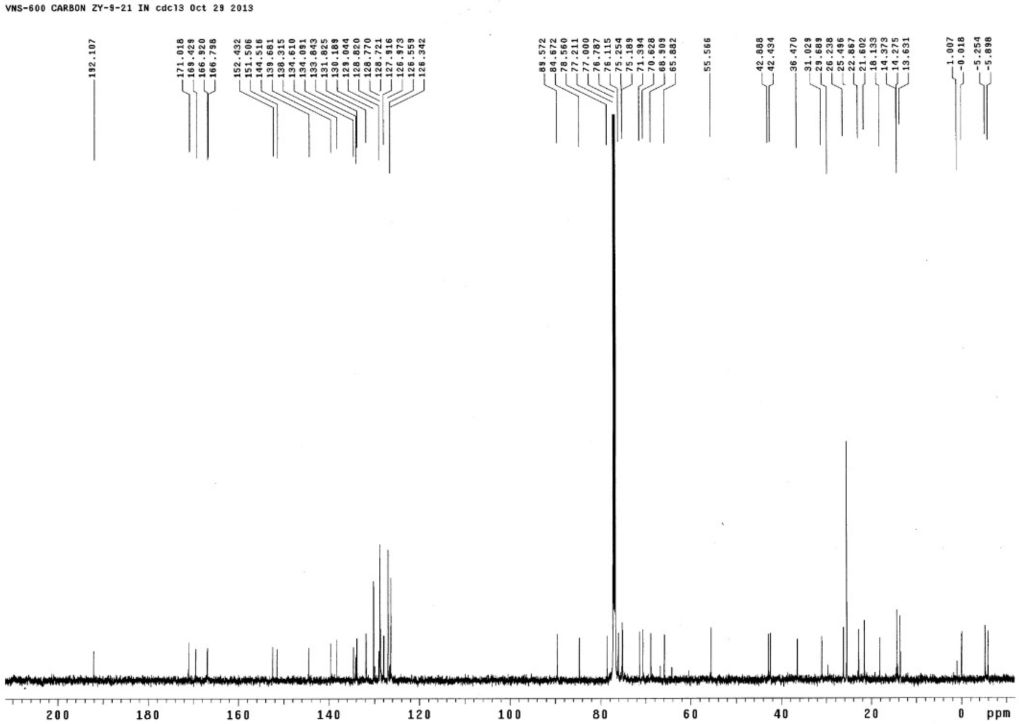
VNS-600 CARBON ZY-9-21 IN cdcl3 Oct 29 2013
192.107
171.018
169.429
166.920
166.798
152.432
151.506
144.516
139.681
138.315
134.610
134.091
133.843
131.825
130.189
129.044
128.820
128.770
128.721
127.916
126.973
126.559
126.342
89.572
84.672
78.560
77.211
77.000
76.787
76.115
75.254
75.189
71.394
70.628
68.909
65.882
55.566
42.888
42.434
36.470
31.029
29.689
26.238
25.496
22.867
21.602
18.133
14.373
14.275
13.631
1.007
-0.018
-5.254
-5.898
200
180
160
140
120
100
80
60
40
20
0
ppm

ESI-MS, ^{1}H-NMR and ^{13}C-NMR spectra of compound **19b**

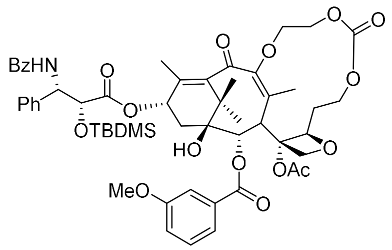

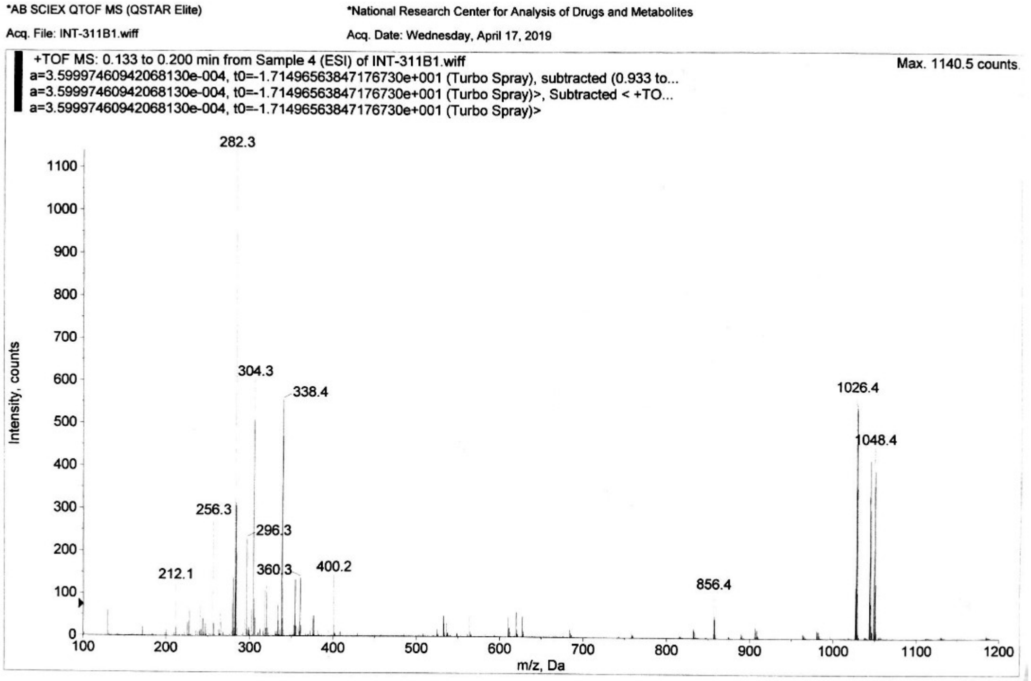

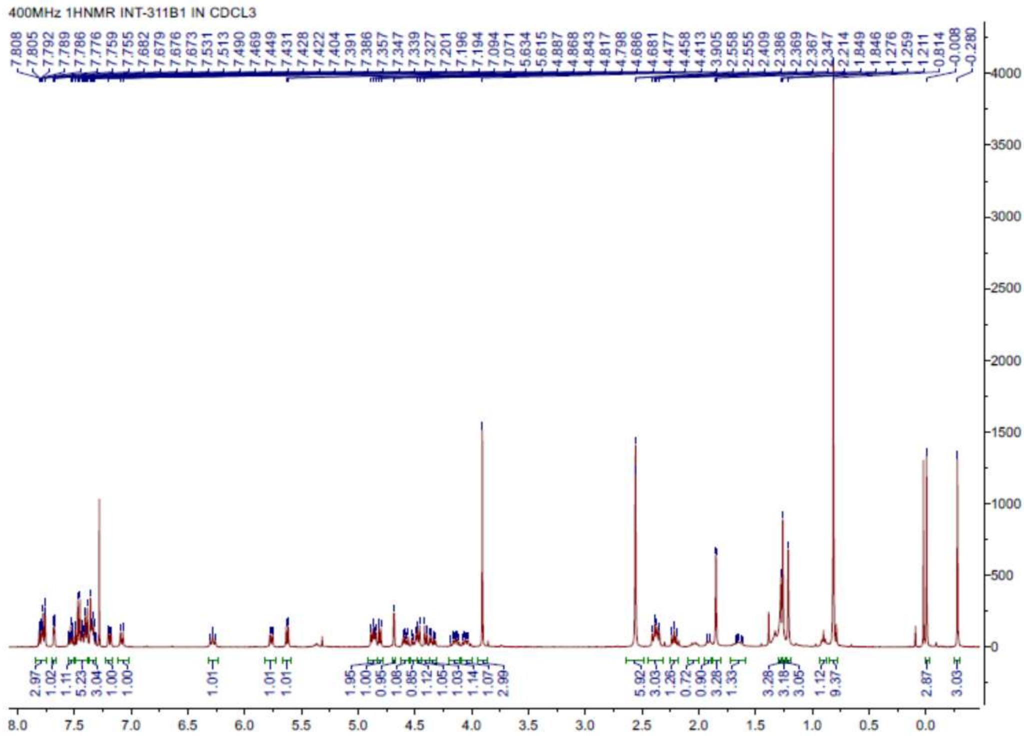
400MHz 1HNMR INT-311B1 IN CDCL3
7.808
7.805
7.792
7.789
7.786
7.776
7.759
7.755
7.682
7.679
7.676
7.673
7.531
7.513
7.490
7.469
7.449
7.431
7.428
7.422
7.404
7.391
7.386
7.357
7.347
7.339
7.327
7.201
7.196
7.194
7.094
7.071
5.634
5.615
4.887
4.868
4.843
4.817
4.798
4.686
4.681
4.477
4.458
4.413
3.905
2.558
2.555
2.409
2.386
2.369
2.367
2.347
2.214
1.849
1.846
1.276
1.259
1.211
0.814
-0.008
-0.280

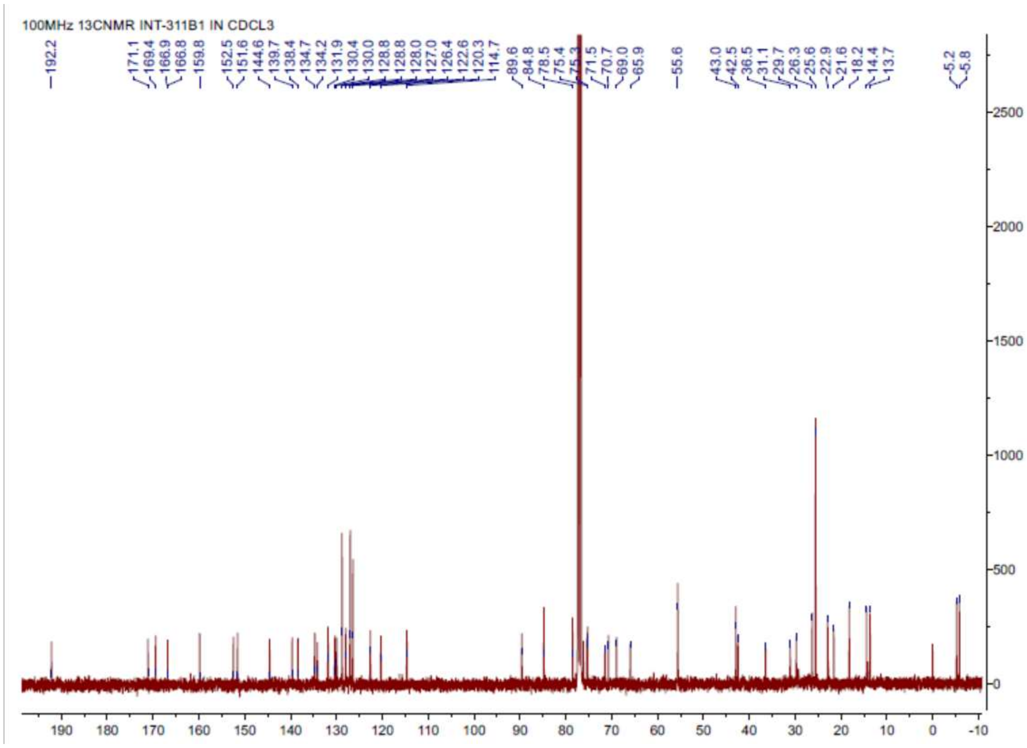
100MHz 13CNMR INT-311B1 IN CDCL3
192.2
171.1
169.4
166.9
166.8
159.8
152.5
151.6
144.6
139.7
138.4
134.7
134.2
131.9
130.4
130.0
128.8
128.8
128.0
127.0
126.4
122.6
120.3
114.7
89.6
84.8
78.5
75.4
75.3
71.5
70.7
69.0
65.9
55.6
43.0
42.5
36.5
31.1
29.7
26.3
25.6
22.9
21.6
18.2
14.4
13.7
-5.2
-5.8

ESI-MS, ^{1}H-NMR and ^{13}C-NMR spectra of compound **19c**

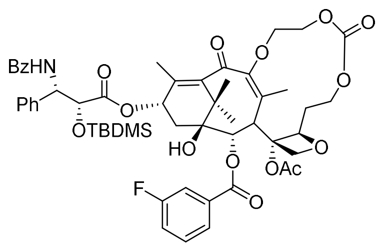

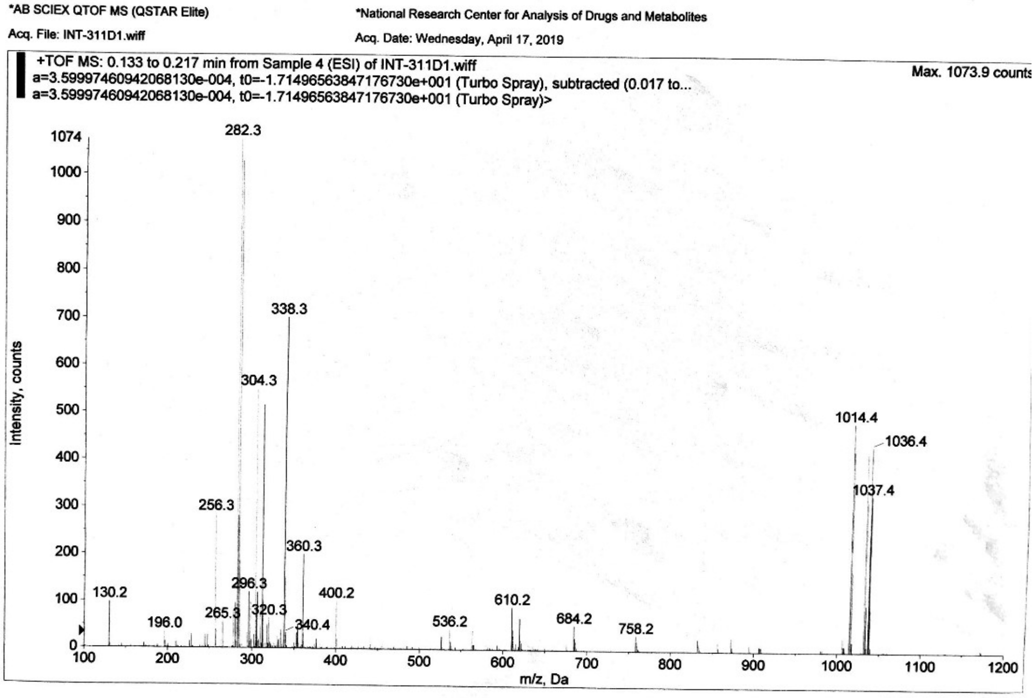

400MHz 1HNMR INT-311D1 IN CDCL3

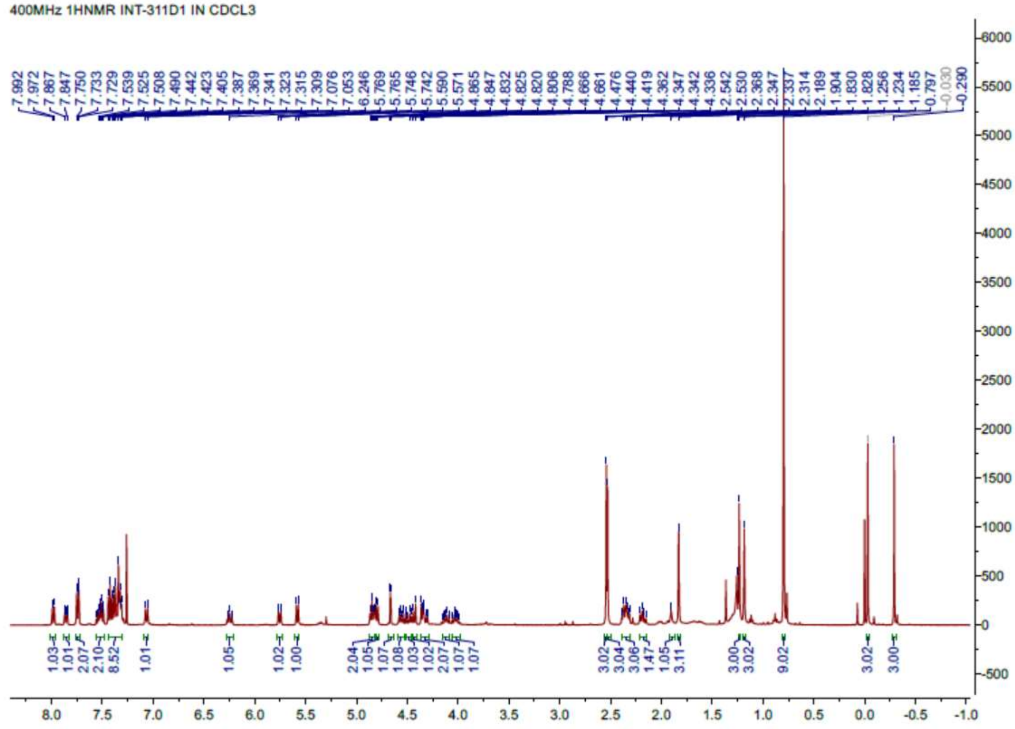

100MHz 13CNMR INT-311D1 IN CDCL3

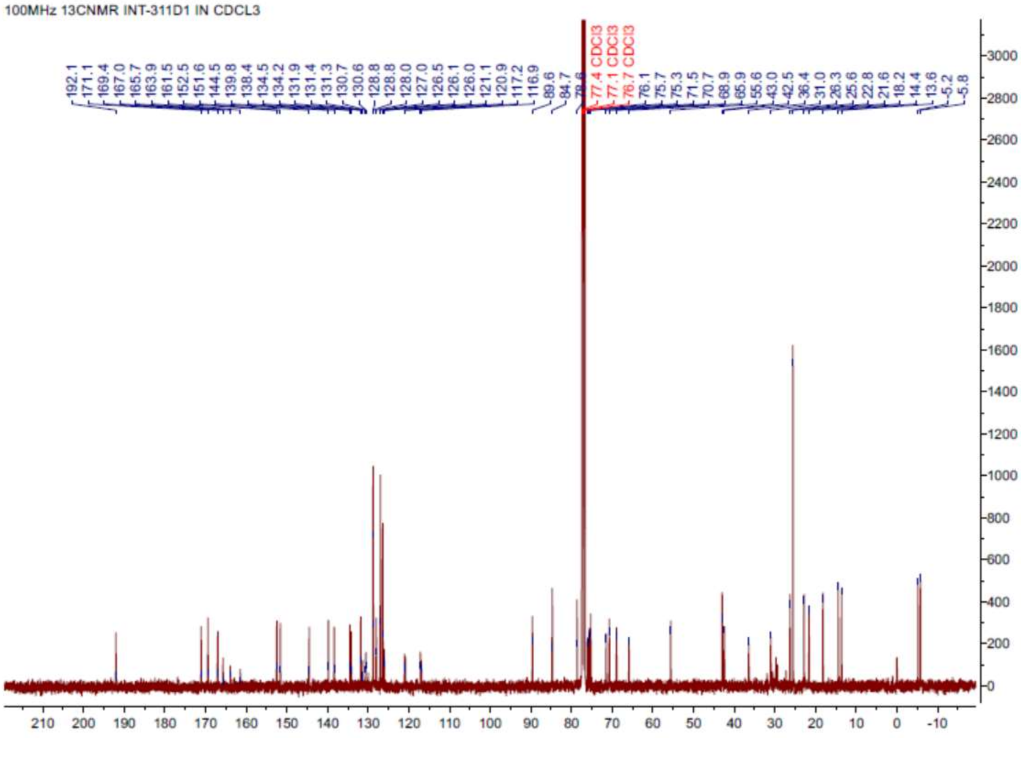

ESI-MS, ^{1}H-NMR and ^{13}C-NMR spectra of compound **19d**

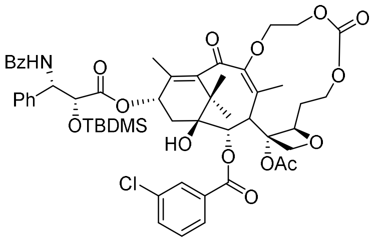

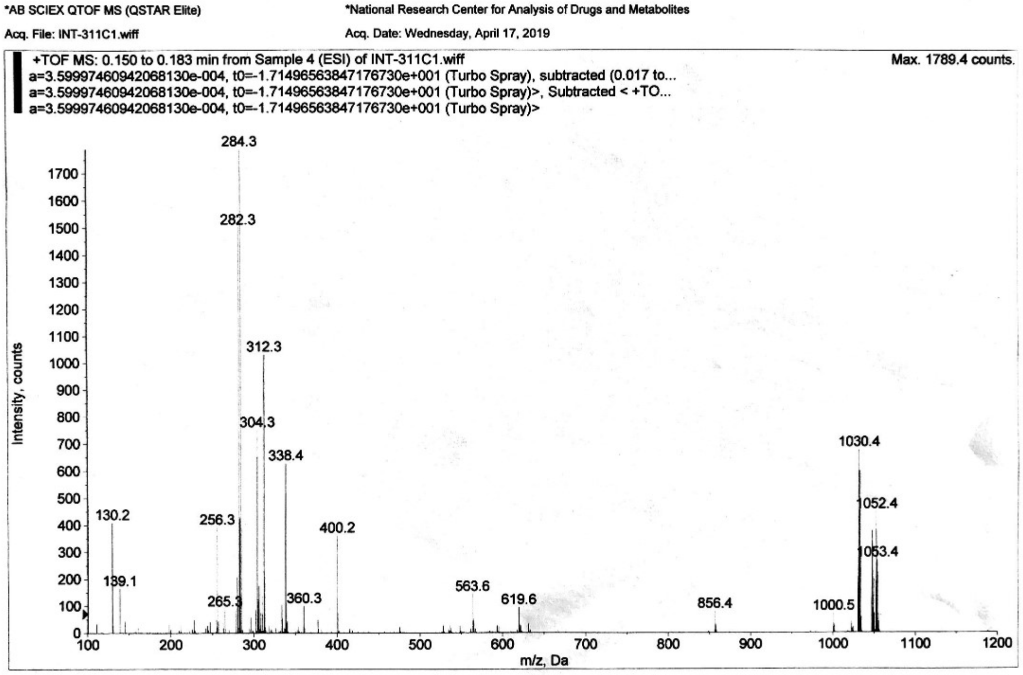

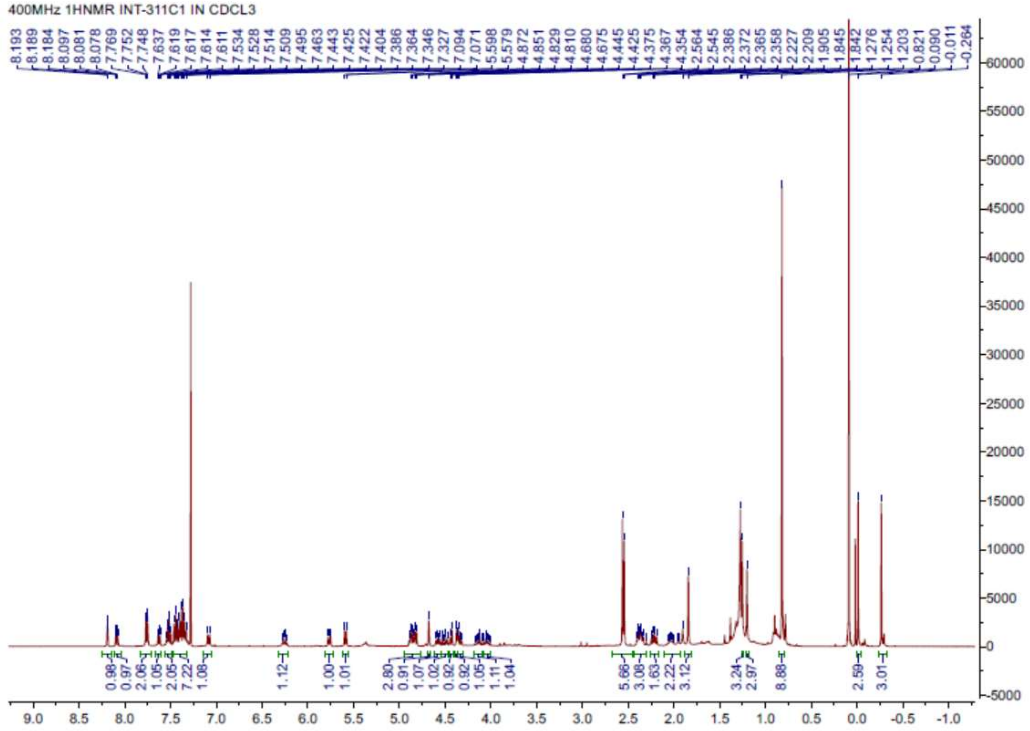
400MHz 1HNMR INT-311C1 IN CDCL3
8.193 8.189 8.184 8.097 8.081 8.078 7.769 7.752 7.748 7.637 7.619 7.617 7.614 7.611 7.534 7.528 7.514 7.509 7.495 7.463 7.443 7.425 7.422 7.404 7.386 7.364 7.346 7.327 7.094 7.071 5.598 5.579 4.872 4.851 4.829 4.810 4.680 4.675 4.445 4.425 4.375 4.367 4.354 2.564 2.545 2.386 2.372 2.365 2.358 2.227 2.209 1.905 1.845 1.842 1.276 1.254 1.203 0.821 0.090 -0.011 -0.264
0.98 0.97 2.06 1.05 2.05 7.22 1.08 1.12 1.00 1.01 2.80 0.91 1.07 1.02 0.92 0.92 1.05 1.11 1.04 5.66 3.08 1.63 2.22 3.12 3.24 2.97 8.88 2.59 3.01
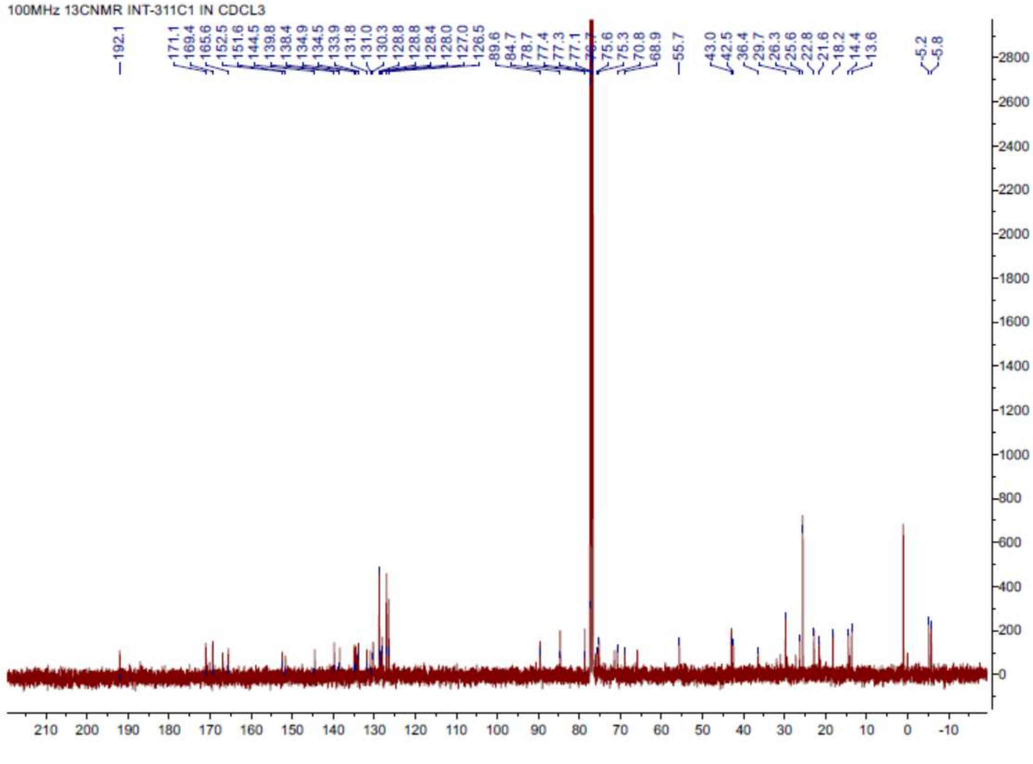
100MHz 13CNMR INT-311C1 IN CDCL3
192.1 171.1 169.4 165.6 152.5 151.6 144.5 139.8 138.4 134.9 134.5 133.9 131.8 131.0 130.3 128.8 128.8 128.4 128.0 127.0 126.5 89.6 84.7 78.7 77.4 77.3 77.1 76.7 75.6 75.3 70.8 68.9 55.7 43.0 42.5 36.4 29.7 26.3 25.6 22.8 21.6 18.2 14.4 13.6 -5.2 -5.8

ESI-MS, ^{1}H-NMR and ^{13}C-NMR spectra of compound **19e**

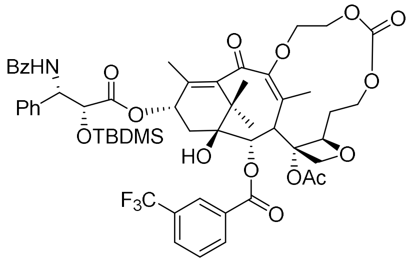

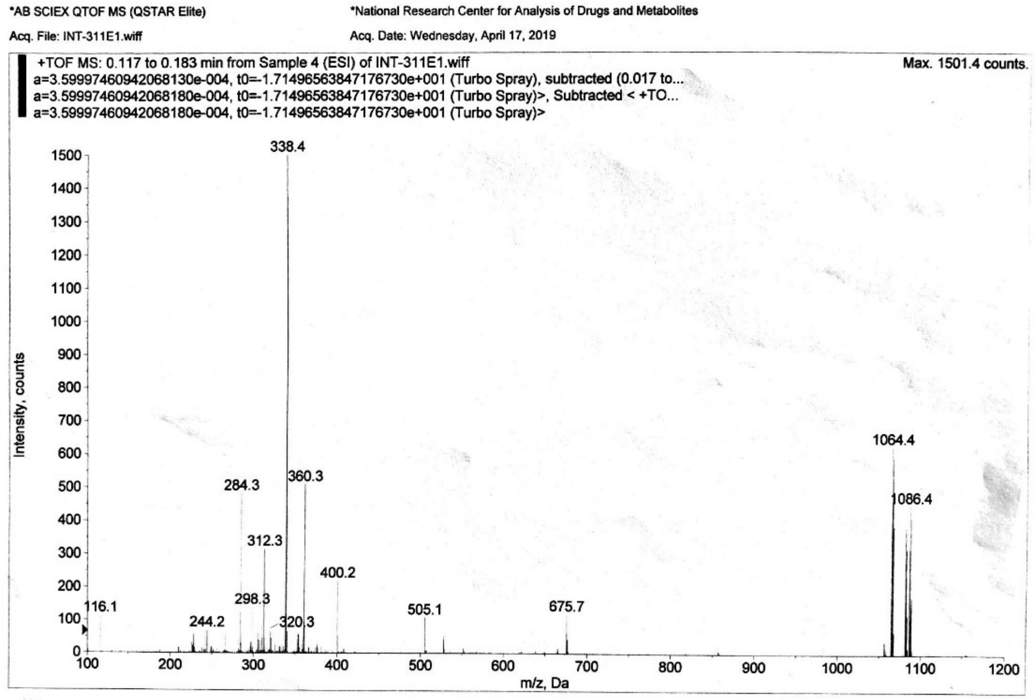

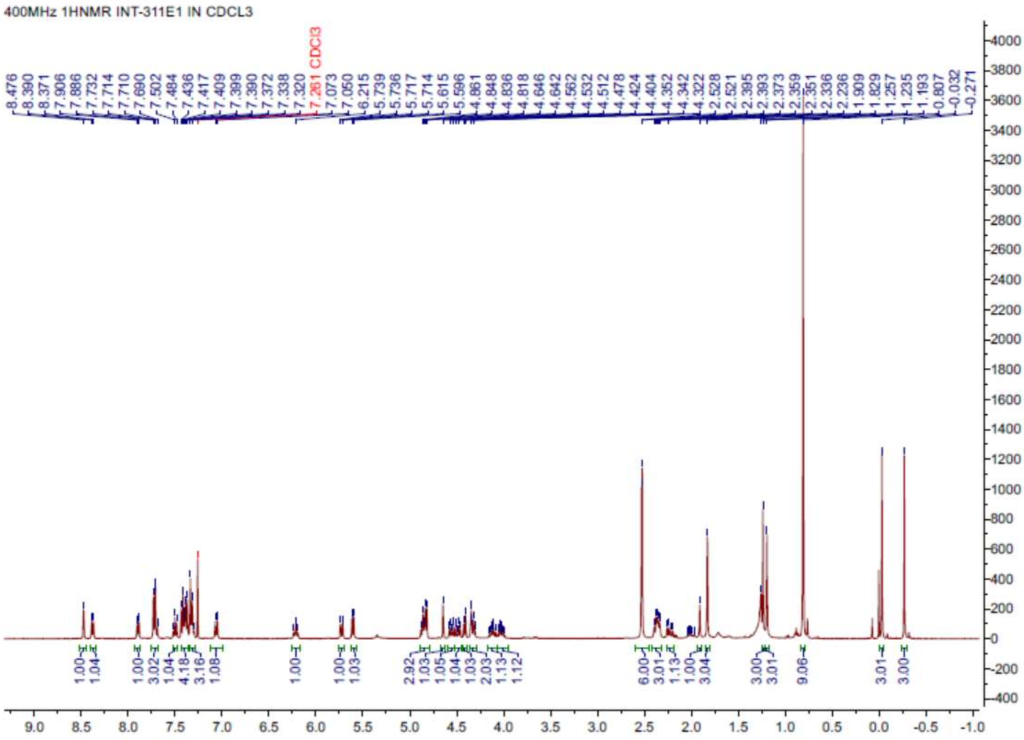
400MHz 1HNMR INT-311E1 IN CDCL3
7.261 CDCl3

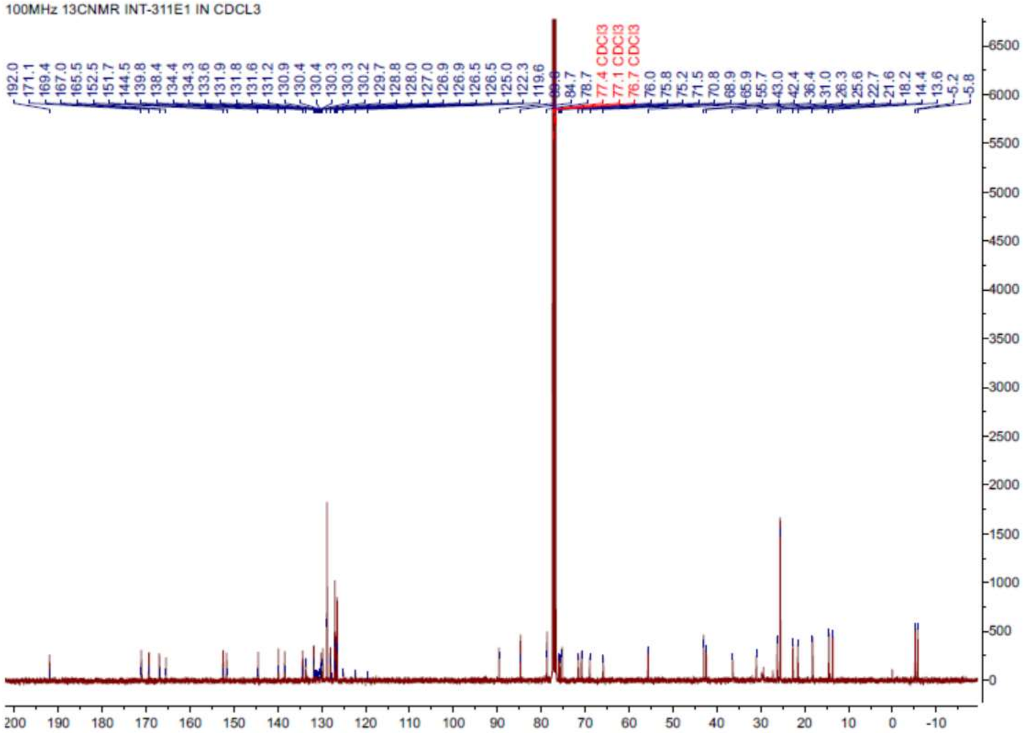
100MHz 13CNMR INT-311E1 IN CDCL3
77.4 CDCl3
77.1 CDCl3
76.7 CDCl3

ESI-MS, ^{1}H-NMR and ^{13}C-NMR spectra of compound **20a**

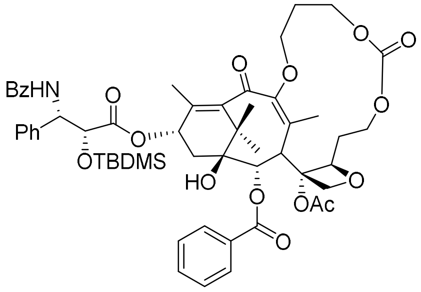

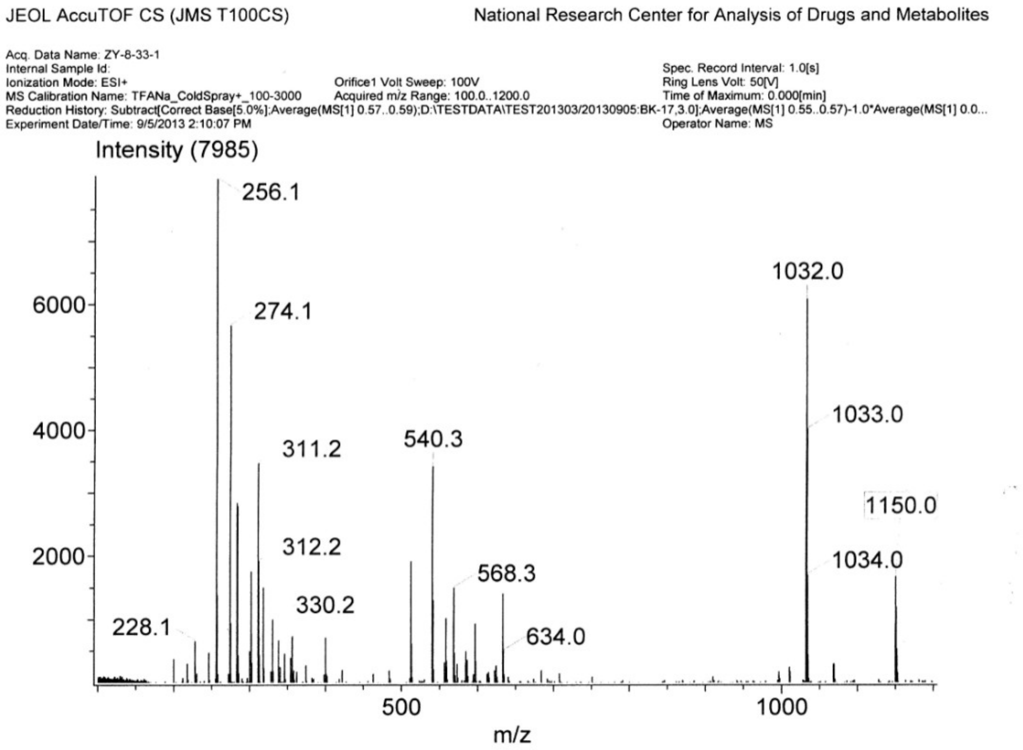

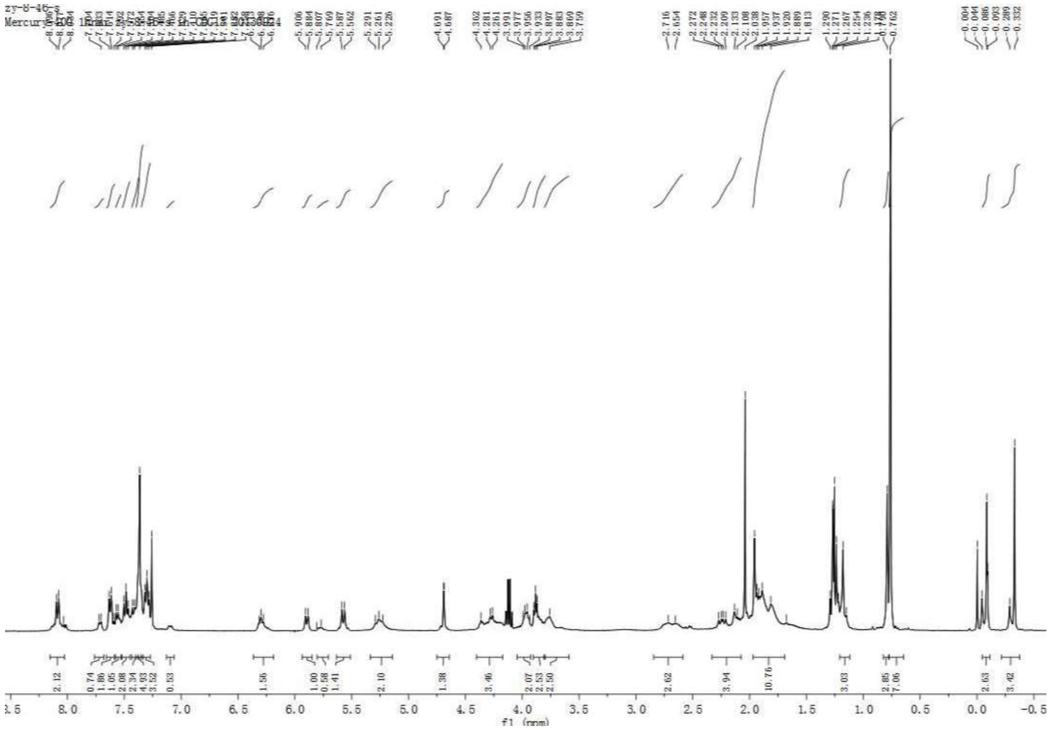

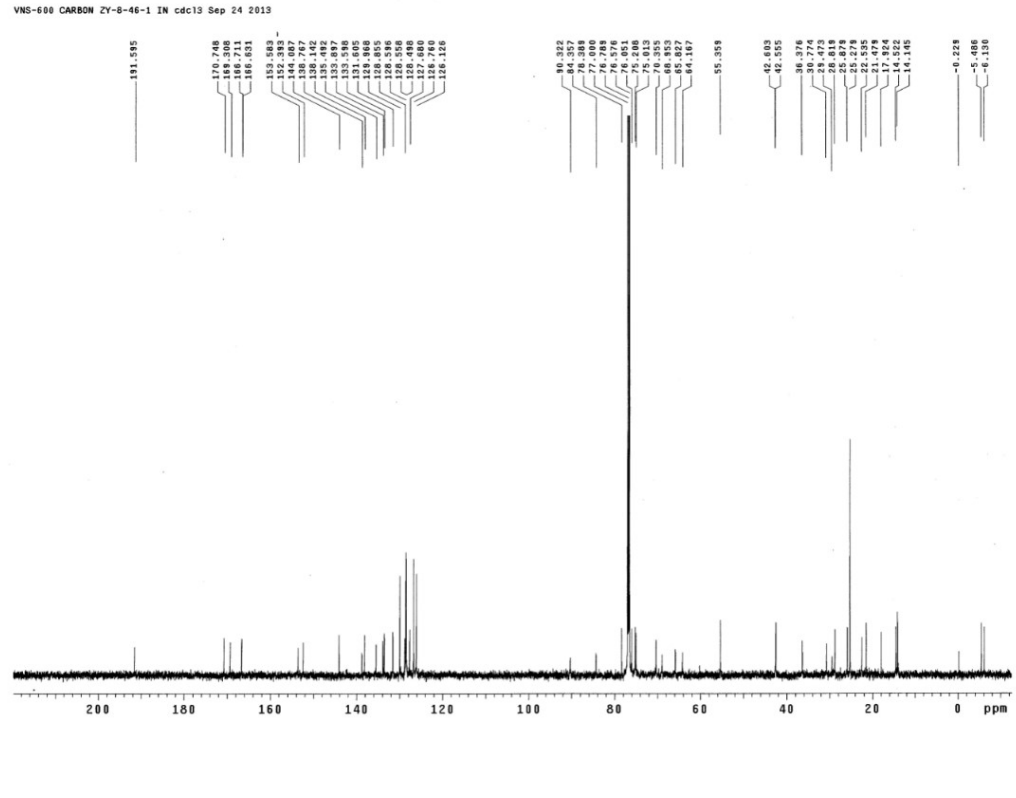

ESI-MS, ^{1}H-NMR and ^{13}C-NMR spectra of compound **20b**

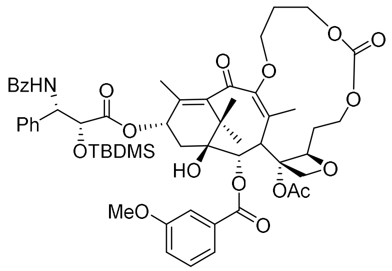

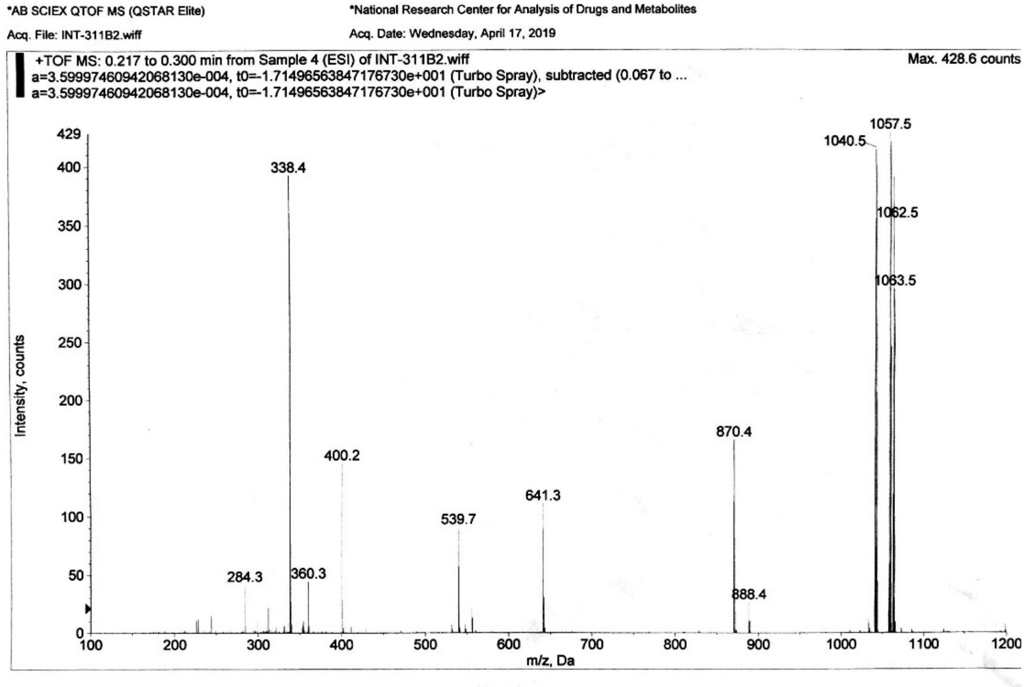

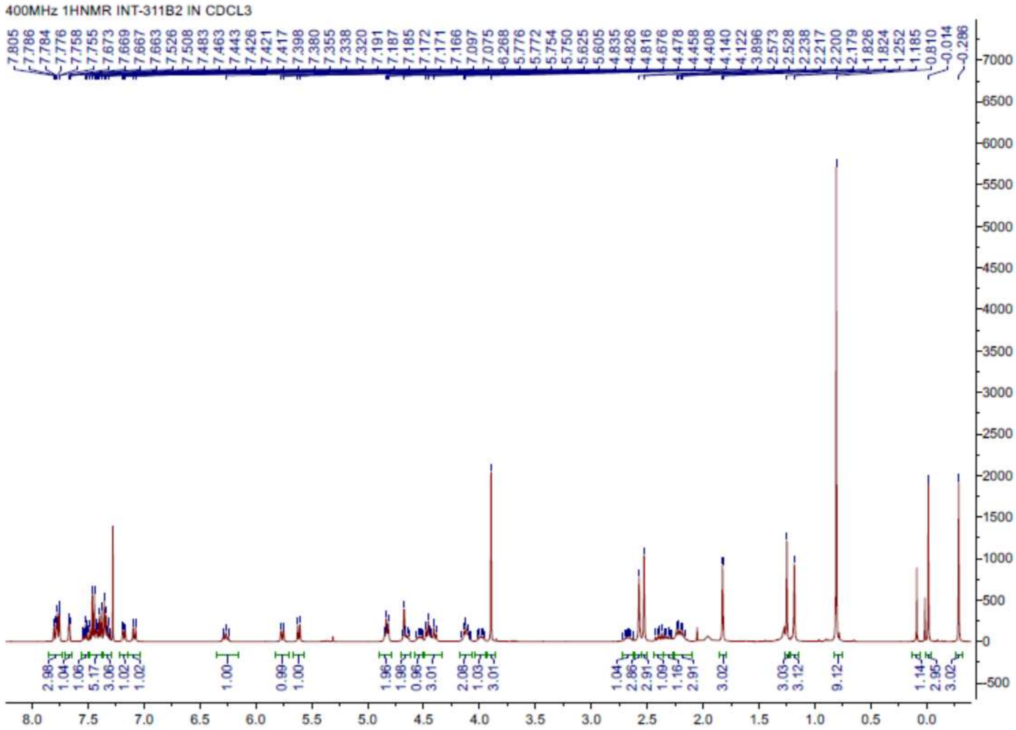
400MHz 1HNMR INT-311B2 IN CDCL3
7.805 7.786 7.784 7.776 7.758 7.755 7.673 7.669 7.667 7.663 7.526 7.508 7.483 7.463 7.443 7.426 7.421 7.417 7.398 7.380 7.355 7.338 7.320 7.191 7.187 7.185 7.172 7.171 7.166 7.097 7.075 6.268 5.776 5.772 5.754 5.750 5.625 5.605 4.835 4.826 4.816 4.676 4.478 4.458 4.408 4.140 4.122 3.896 2.573 2.528 2.238 2.217 2.200 2.179 1.826 1.824 1.252 1.185 0.810 -0.014 -0.286
2.98 1.04 1.06 5.17 3.06 1.02 1.02 1.00 0.99 1.00 1.96 1.98 0.96 3.01 2.08 1.03 3.01 1.04 2.86 2.91 1.09 1.16 2.91 3.02 3.03 3.12 9.12 1.14 2.95 3.02
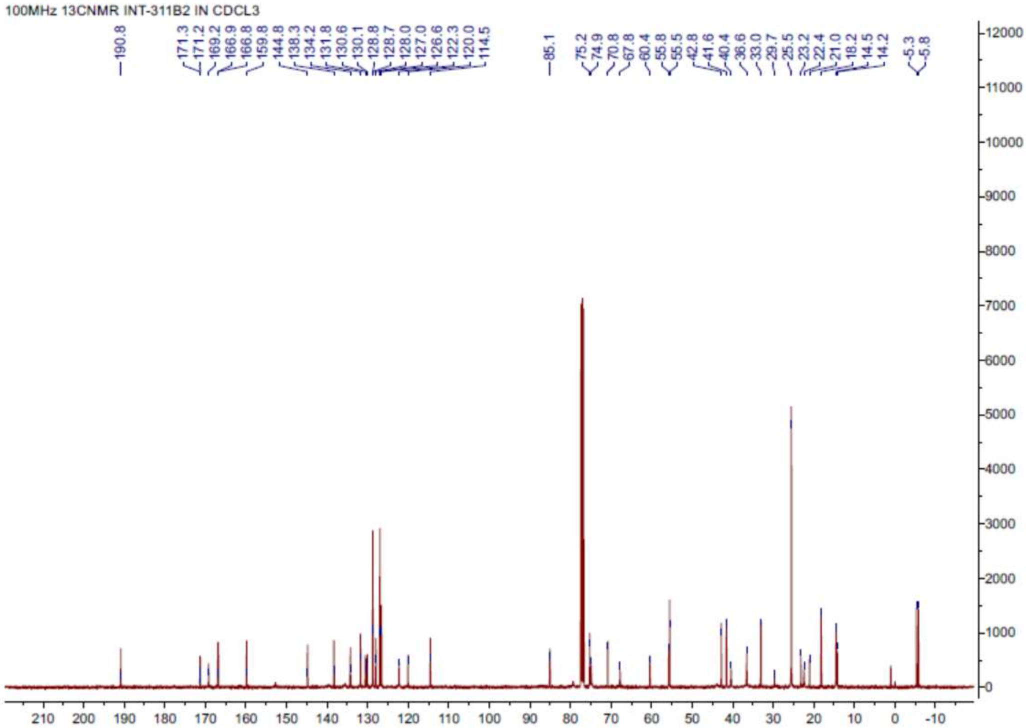
100MHz 13CNMR INT-311B2 IN CDCL3
190.8 171.3 171.2 169.2 166.9 166.8 159.8 144.8 138.3 134.2 131.8 130.6 130.1 128.8 128.7 128.0 127.0 126.6 122.3 120.0 114.5 85.1 75.2 74.9 70.8 67.8 60.4 55.8 55.5 42.8 41.6 40.4 36.6 33.0 29.7 25.5 23.2 22.4 21.0 18.2 14.5 14.2 -5.3 -5.8

ESI-MS, ^{1}H-NMR and ^{13}C-NMR spectra of compound **20c**

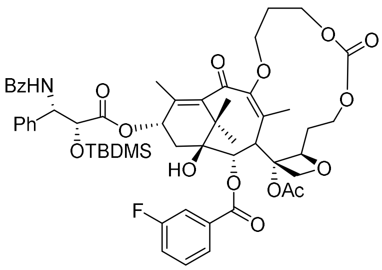

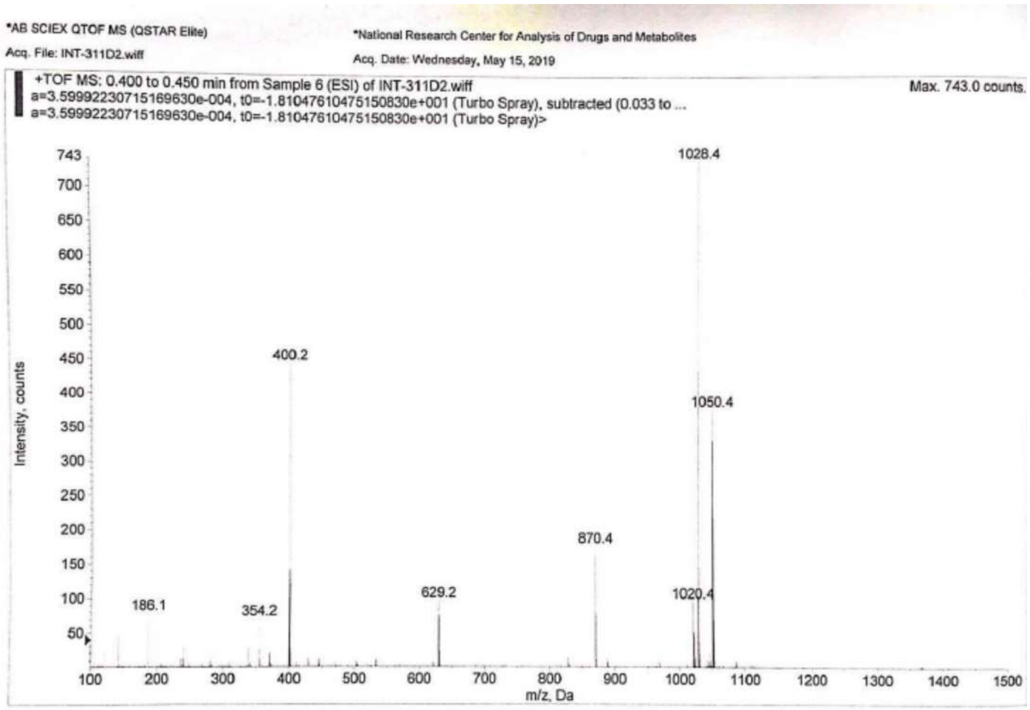

400MHz 1HNMR INT-311D2 IN CDCL3

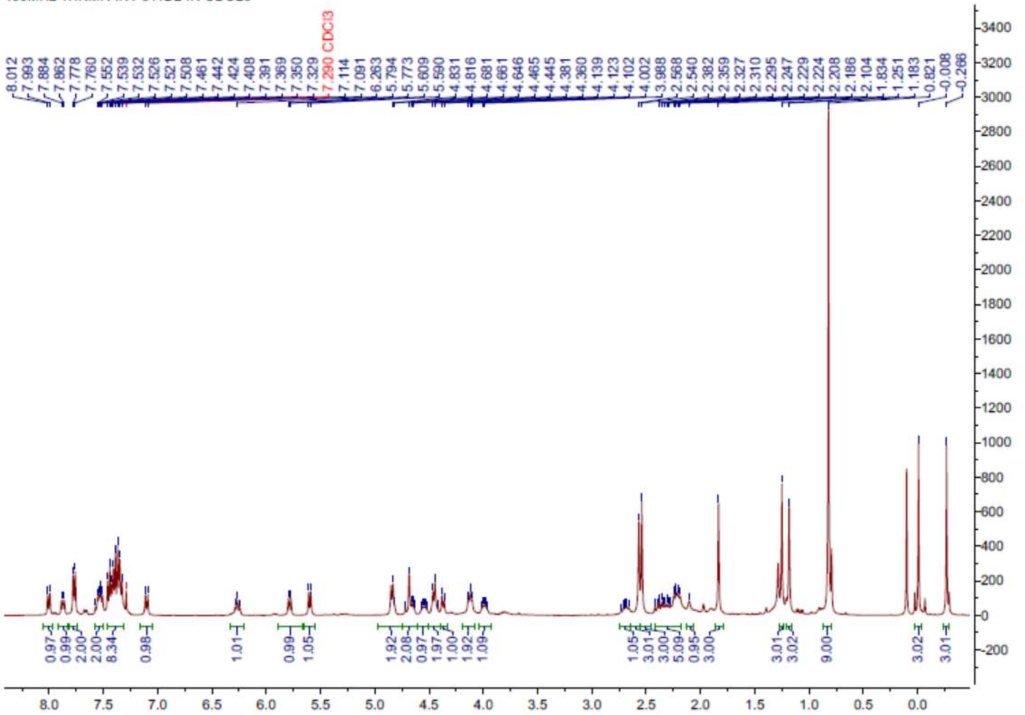

100MHz 13CNMR INT-311D2 IN CDCL3

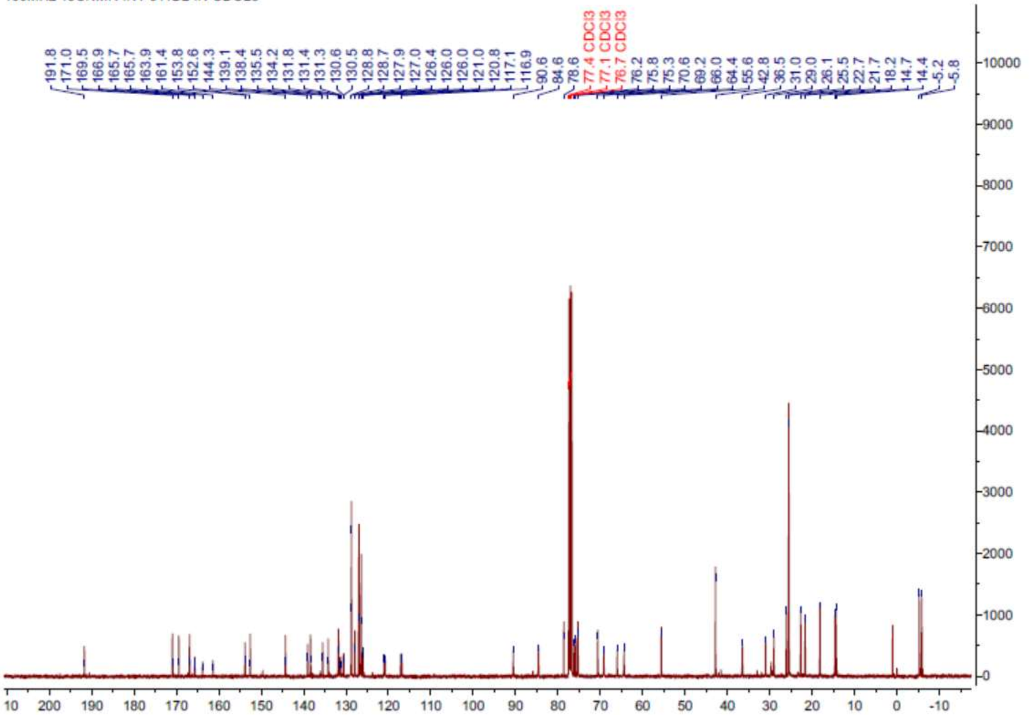

ESI-MS, ^{1}H-NMR and ^{13}C-NMR spectra of compound **20d**

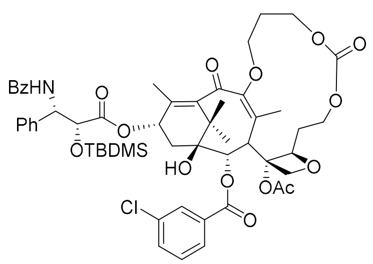

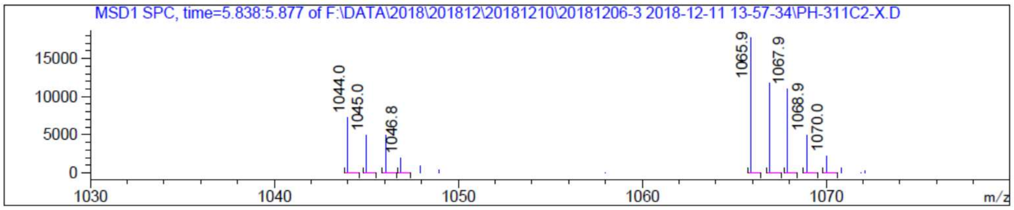

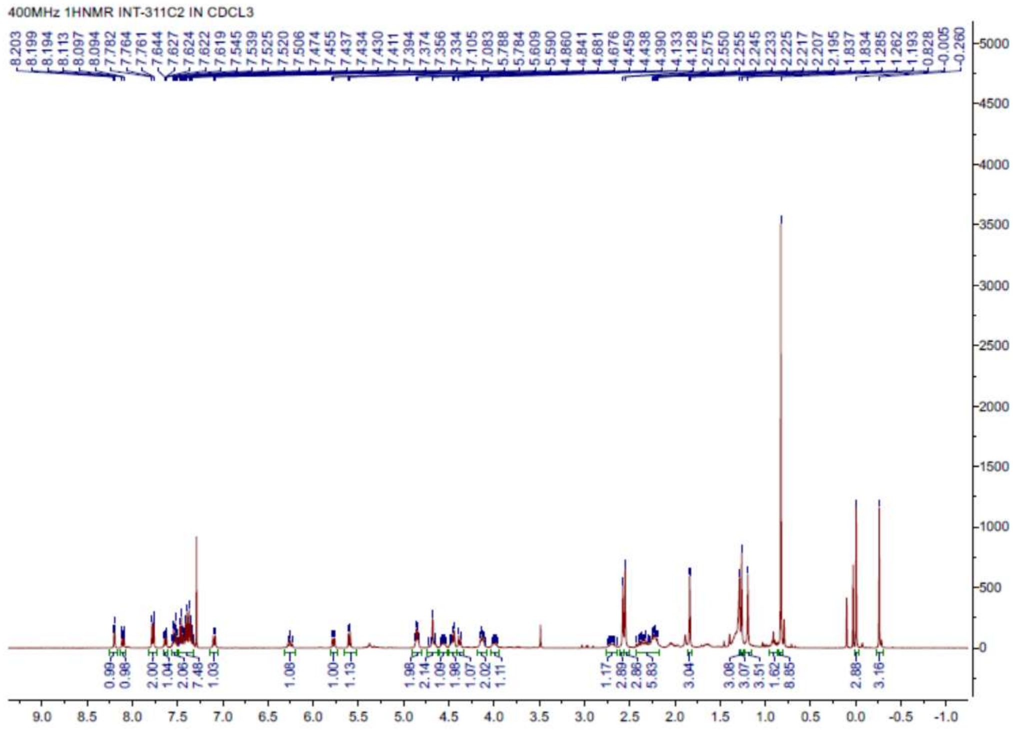

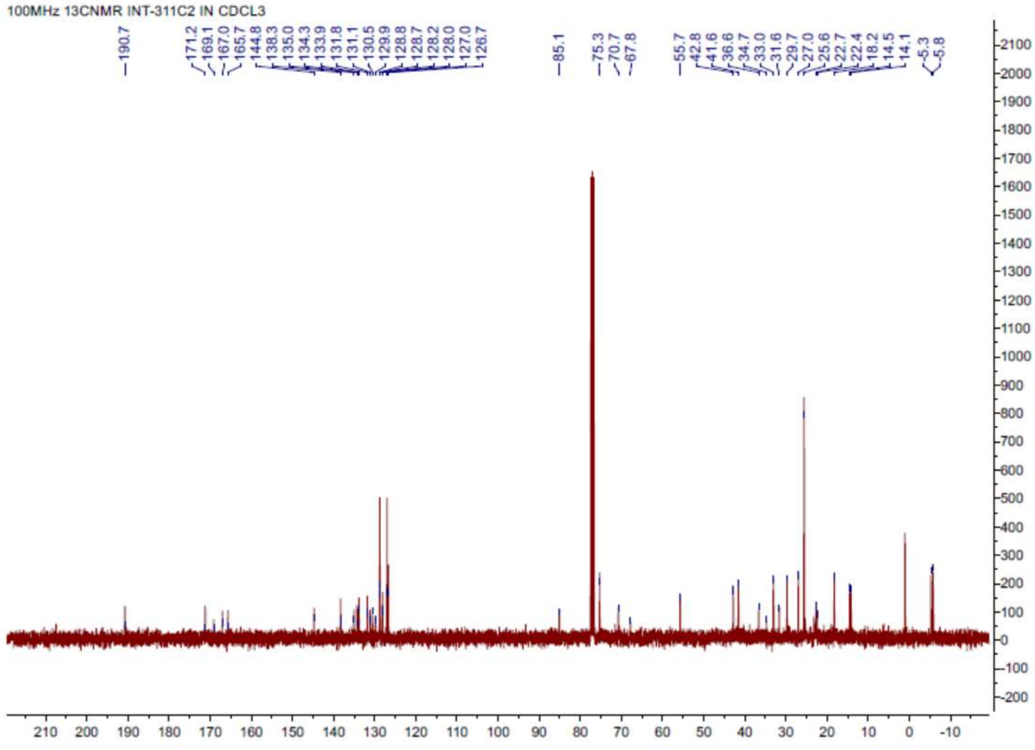
100MHz 13CNMR INT-311C2 IN CDCL3
190.7
171.2
169.1
167.0
165.7
144.8
138.3
135.0
134.3
133.9
131.8
131.1
130.5
129.9
128.8
128.7
128.2
128.0
127.0
126.7
85.1
75.3
70.7
67.8
55.7
42.8
41.6
36.6
34.7
33.0
31.6
29.7
27.0
25.6
22.7
22.4
18.2
14.5
14.1
-5.3
-5.8

ESI-MS, ^{1}H-NMR and ^{13}C-NMR spectra of compound **20e**

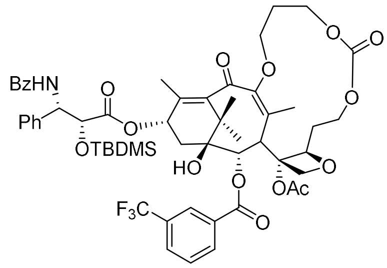

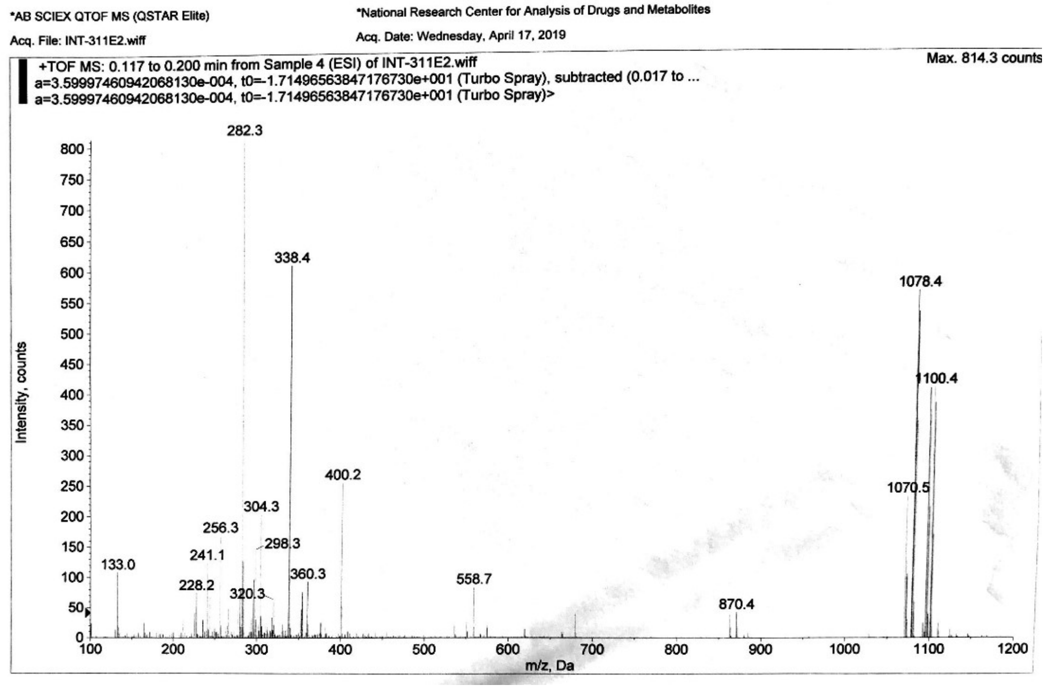

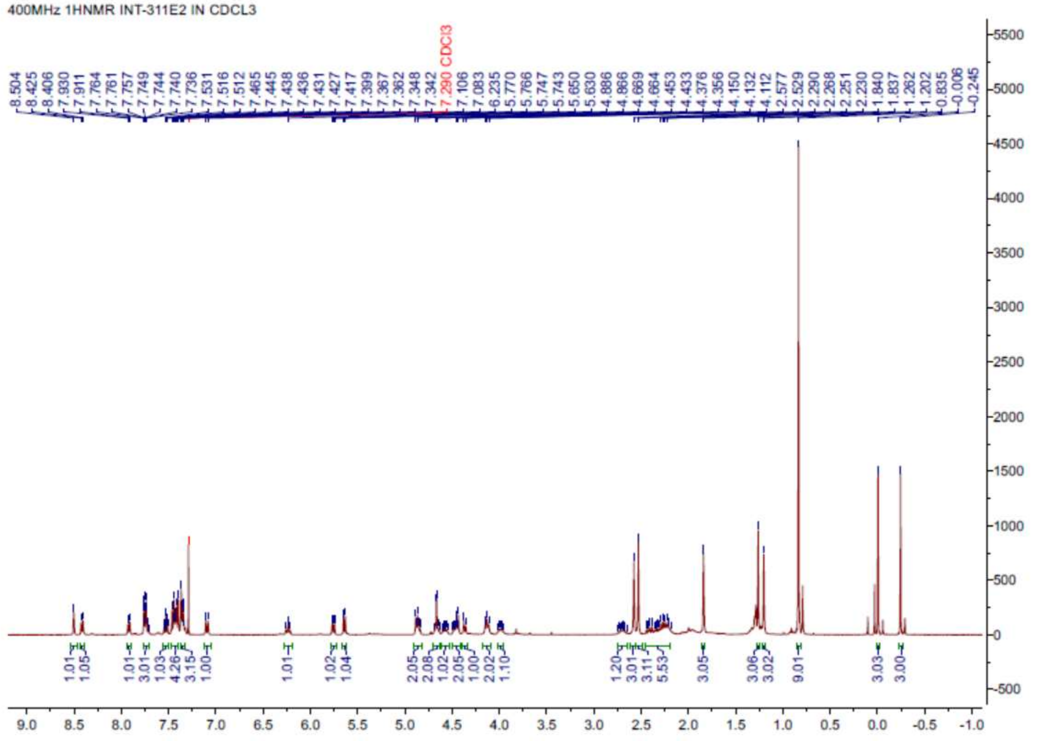
400MHz 1HNMR INT-311E2 IN CDCL3
8.504 8.425 8.406 7.930 7.911 7.764 7.761 7.757 7.749 7.744 7.740 7.736 7.531 7.516 7.512 7.465 7.445 7.438 7.436 7.431 7.427 7.417 7.399 7.367 7.362 7.348 7.342
7.290 CDCl3
7.106 7.083 6.235 5.770 5.766 5.747 5.743 5.650 5.630 4.886 4.669 4.664 4.453 4.433 4.376 4.356 4.150 4.132 4.112 2.577 2.529 2.290 2.288 2.251 2.230 1.840 1.837 1.262 1.202 0.835 0.006 -0.245

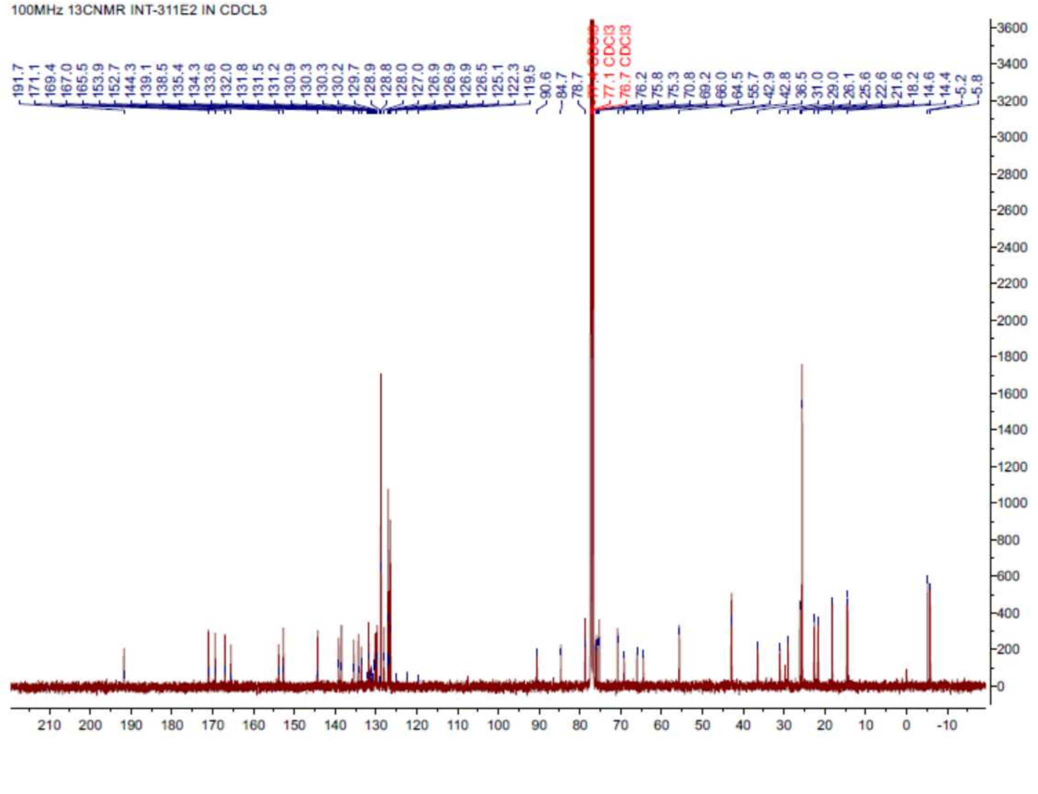
100MHz 13CNMR INT-311E2 IN CDCL3
191.7 171.1 169.4 167.0 165.5 153.9 152.7 144.3 139.1 138.5 135.4 134.3 133.6 132.0 131.8 131.5 131.2 130.9 130.3 130.3 130.2 129.7 128.9 128.8 128.0 127.0 126.9 126.9 126.9 126.5 125.1 122.3 119.5 90.6 84.7 78.7
77.1 CDCl3
76.7 CDCl3
76.2 75.8 75.3 70.8 69.2 66.0 64.5 55.7 42.9 42.8 36.5 31.0 29.0 26.1 25.6 22.6 21.6 18.2 14.6 14.4 -5.2 -5.8

ESI-MS, HPLC, ^{1}H-NMR and ^{13}C-NMR spectra of compound **21a**

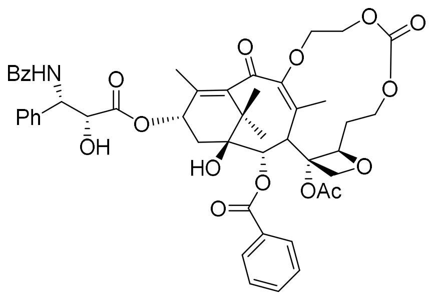

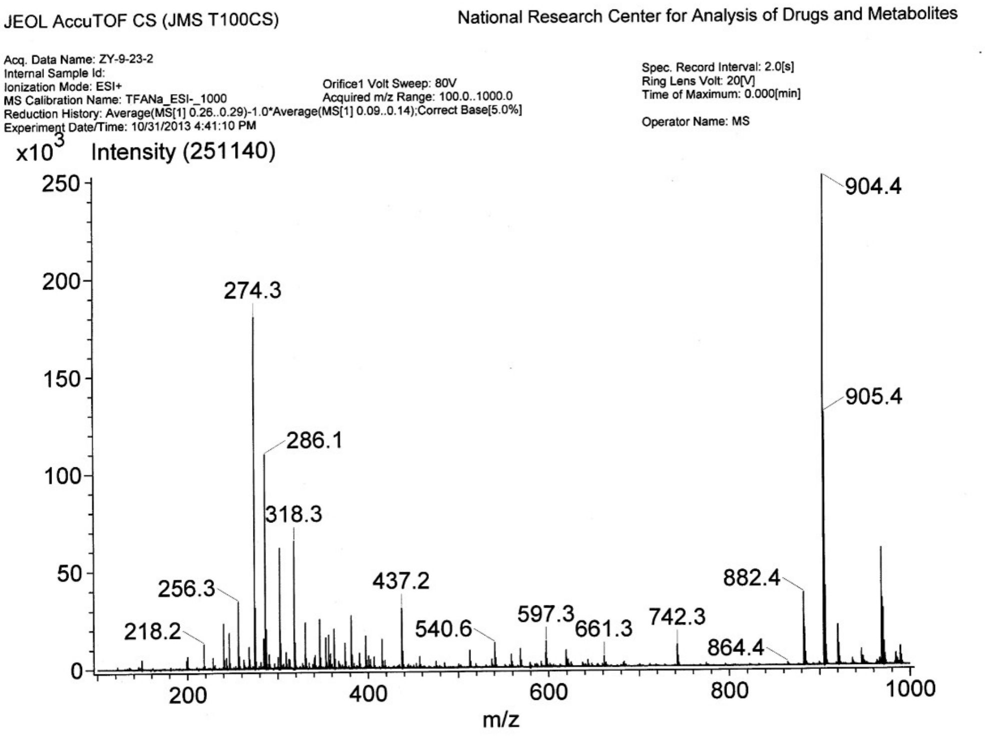

Sample Name : P1
Injection Date : Thu, 20. Dec. 2018 Seq. Line : 12
Inj. Volumn : 10.0 ul
Acq Operator :
Acq Method : F:\DATA\2018\201812\20181220\20181220-3 2018-12-20 19-01-52\MTH-GEN-MS.M
HPLC Analysis Conditions
1. Column : XDB C18 4.6*50mm 1.8um
2. Mobile Phase : A:Water+0.05%TFA B :ACN+0.05%TFA

	Time	A%	B%
3. Flow Mode :	0.00	90.0	10.0
	4.50	0.0	100.0
	6.50	0.0	100.0
	6.60	90.0	10.0

4. Flow : Start Flow : 1.0 ml/min

5. UV Wavelength: C: 220nm ;

6. Column Temp. : Left : 40.0C Right : 40.0C

7. Sample Preparation:

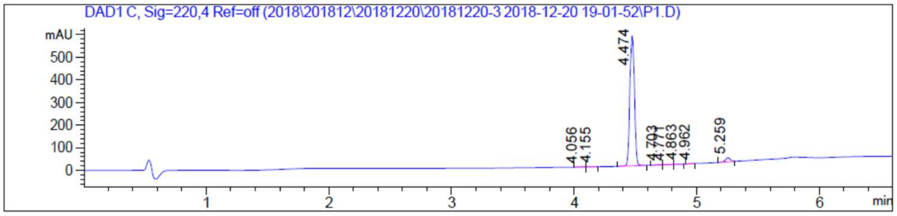

#	Meas. Ret. Time	Height	Height %	Area	Area %
1	4.056	1.042	0.173	2.709	0.172
2	4.155	0.854	0.142	2.477	0.157
3	4.474	576.164	95.863	1506.776	95.760
4	4.703	0.702	0.117	2.312	0.147
5	4.771	1.199	0.200	3.148	0.200
6	4.863	1.095	0.182	2.873	0.183
7	4.962	1.040	0.173	2.837	0.180
8	5.259	18.929	3.149	50.368	3.201

Bruker AVIIIHD 600 20131129
ZY-9-41-2
PROTON CDCl3 D:\\ DATA2013 18

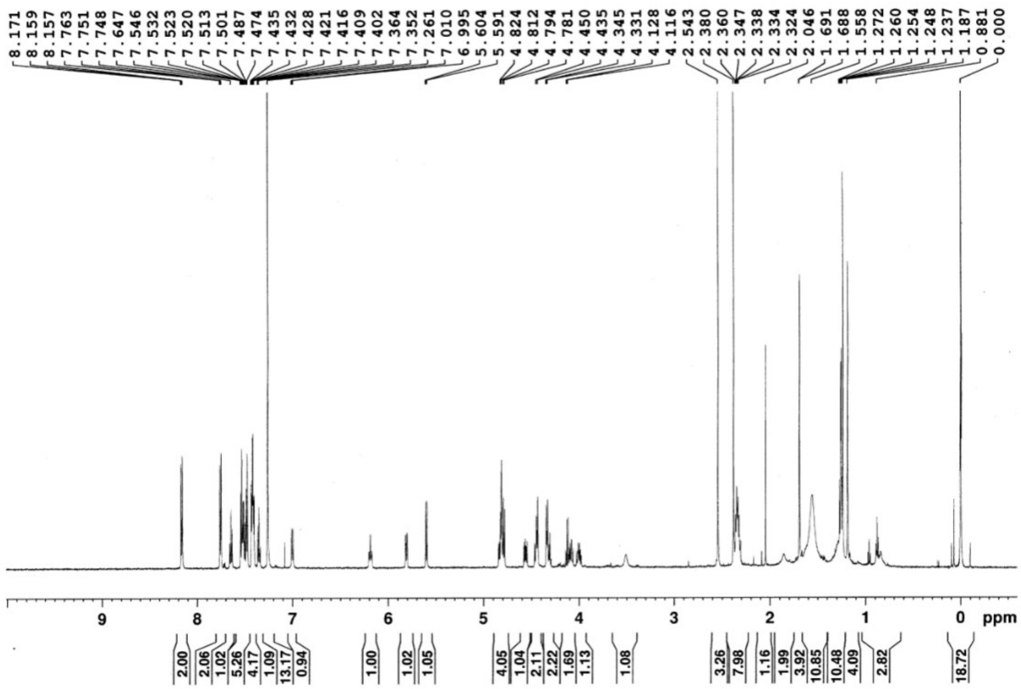

Bruker AVIIIHD 600 20131129
ZY-9-41-2 C13 CDCl3 NS=8000 D:\\ DATA2013 18

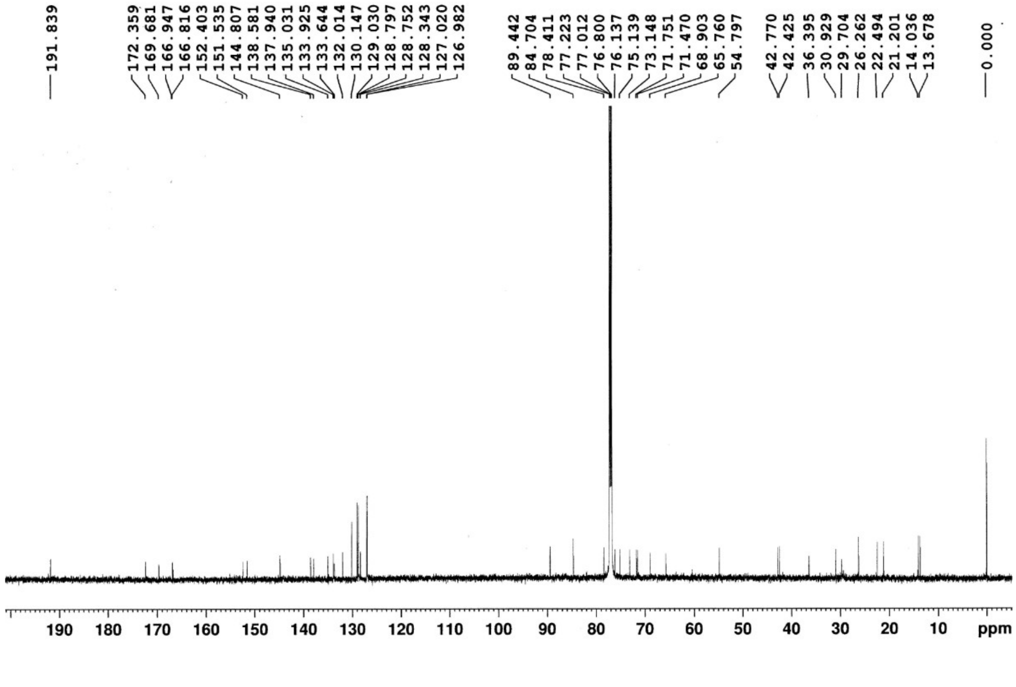

ESI-MS, HPLC, ^{1}H-NMR and ^{13}C-NMR spectra of compound **21b**

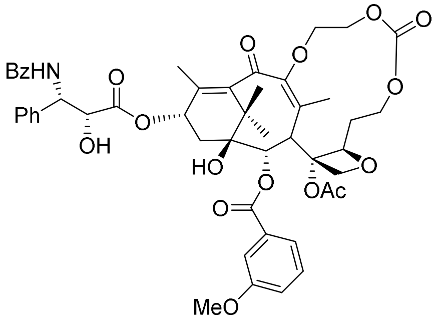

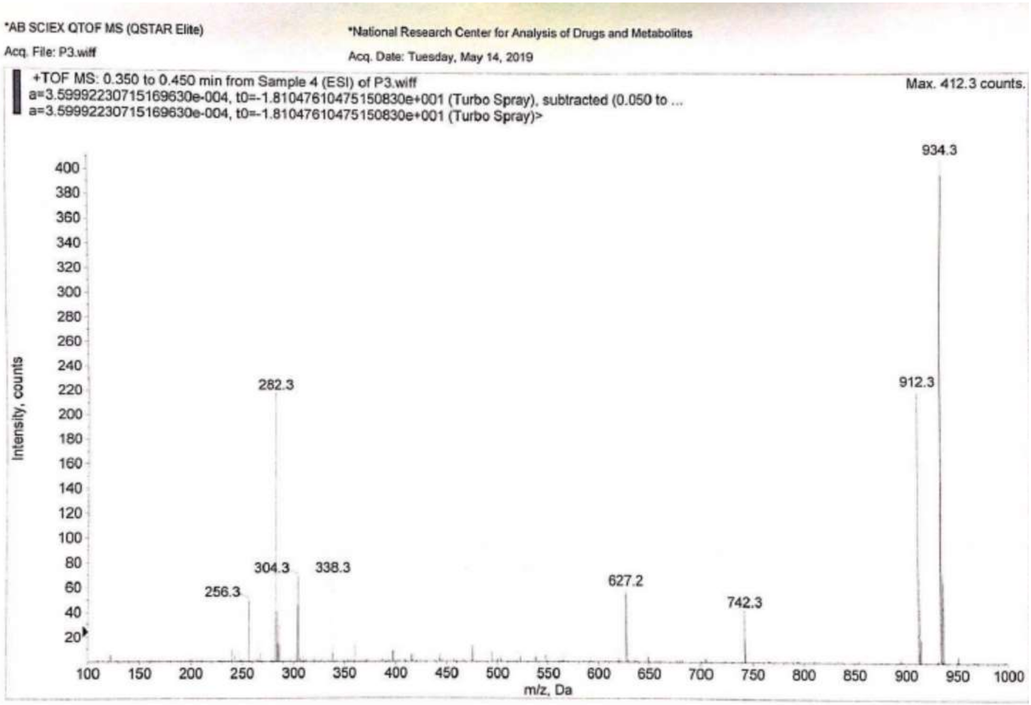

```
Sample Name       : P3
Injection Date    : Thu, 6. Dec. 2018                    Seq. Line :   1
Inj. Volumn       : 10.0 ul
Acq Operator      :
Acq   Method      : F:\DATA\2018\201812\20181205\20181206-1 2018-12-06 15-00-05\MTH-GEN-
                    MS.M
                    HPLC Analysis Conditions

1. Column        : XDB C18  4.6*50mm  1.8um
2. Mobile Phase :  A:Water+0.05%TFA     B :ACN+0.05%TFA
                  Time                A%                  B%
3. Flow Mode     : 0.00               90.0                10.0
                  4.50                0.0                 100.0
                  6.50                0.0                 100.0
                  6.60                90.0                10.0
 4. Flow          :   Start Flow :  1.0  ml/min

 5. UV Wavelength:   C: 220nm ;

 6. Column Temp. :   Left :   40.0C    Right :     40.0C

 7. Sample Preparation:
```

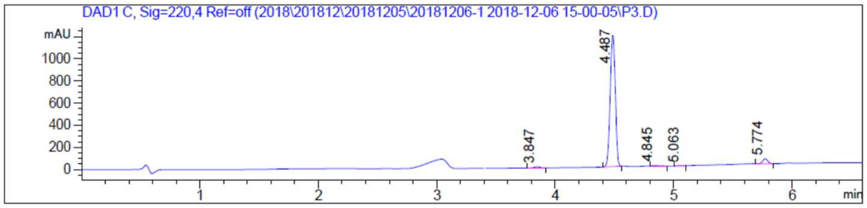

#	Meas. Ret. Time	Height	Height %	Area	Area %
1	3.847	11.055	0.880	36.969	0.991
2	4.487	1193.638	95.000	3544.731	95.037
3	4.845	4.577	0.364	15.473	0.415
4	5.063	2.493	0.198	6.825	0.183
5	5.774	44.704	3.558	125.850	3.374

400MHz 1H-NMR P3 IN CDCL3

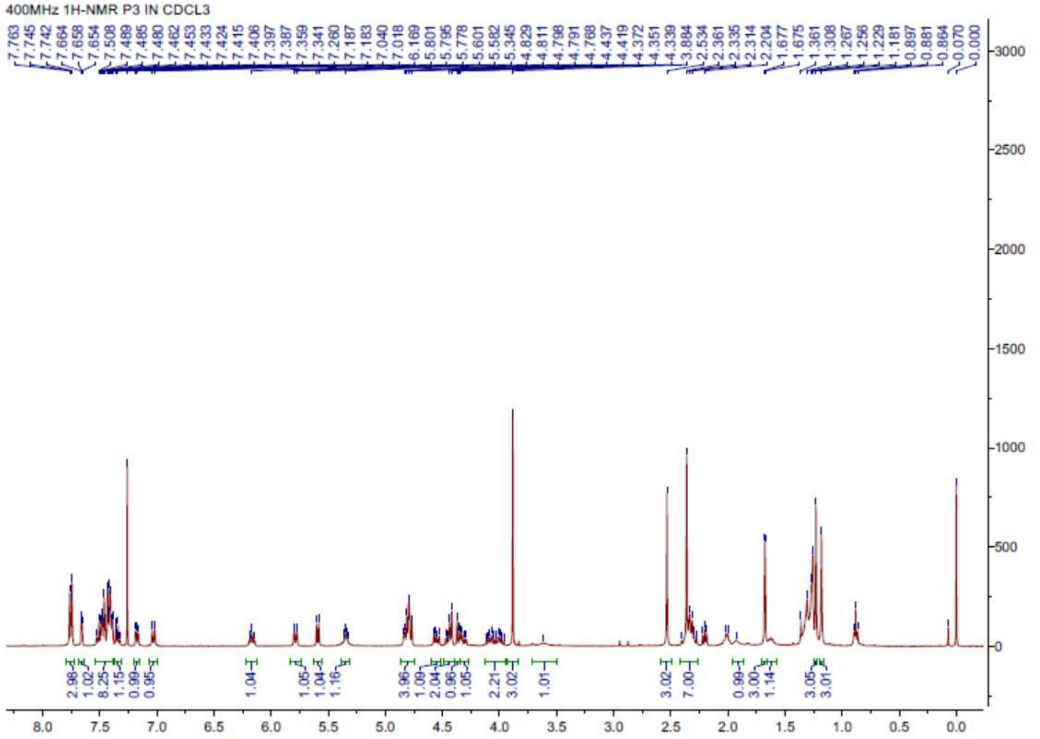

100MHz 13C-NMR P3 IN CDCL3

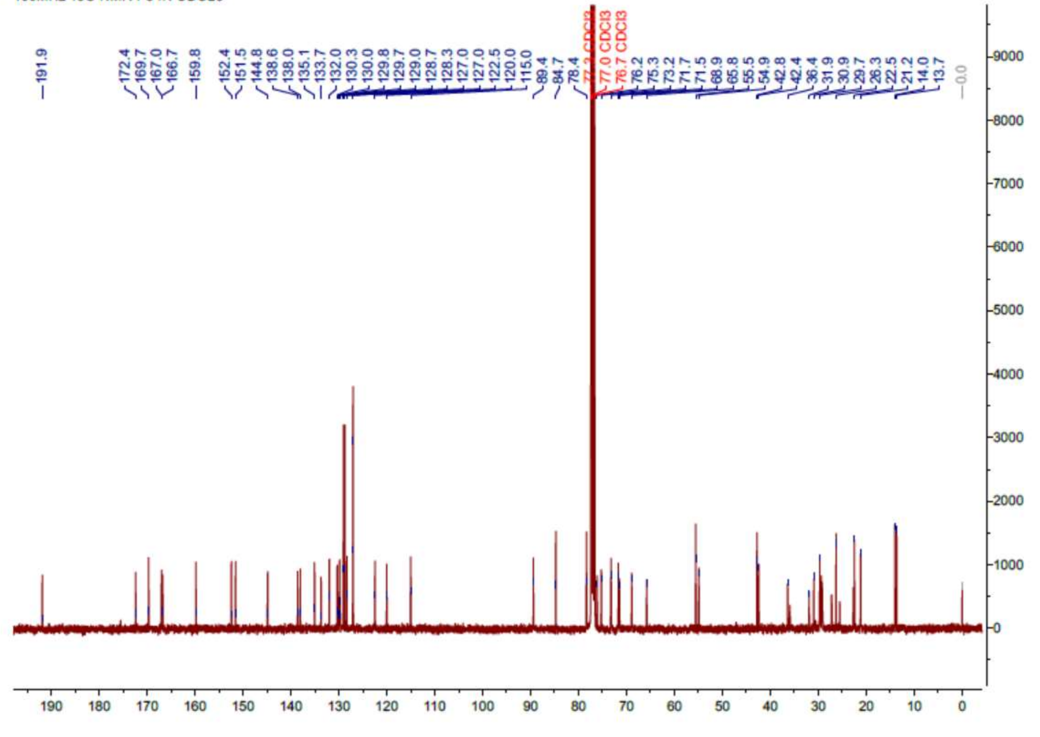

ESI-MS, HPLC, ^{1}H-NMR and ^{13}C-NMR spectra of compound **21c**

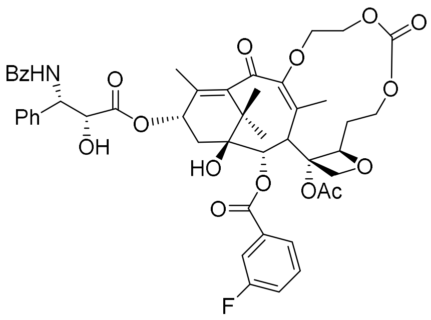

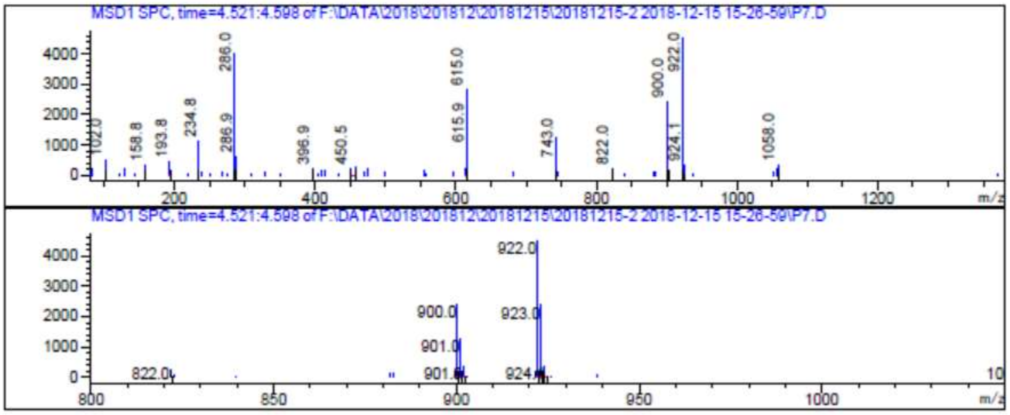

```
Sample Name       : P7
Injection Date    : Sat, 15. Dec. 2018                      Seq. Line :    12
Inj. Volumn       : 10.0 ul
Acq Operator      :
Acq   Method      : F:\DATA\2018\201812\20181215\20181215-2 2018-12-15 15-26-59\MTH-GEN-
                    MS.M
                    HPLC Analysis Conditions

1. Column        : XDB C18  4.6*50mm  1.8um
2. Mobile Phase :  A:Water+0.05%TFA     B :ACN+0.05%TFA
                   Time                A%                  B%
3. Flow Mode     : 0.00               90.0                10.0
                   4.50                0.0               100.0
                   6.50                0.0               100.0
                   6.60               90.0                10.0
 4. Flow          :   Start Flow :  1.0  ml/min

 5. UV Wavelength:   C: 220nm ;

 6. Column Temp. :   Left :   39.5C    Right :     39.5C

 7. Sample Preparation:
```

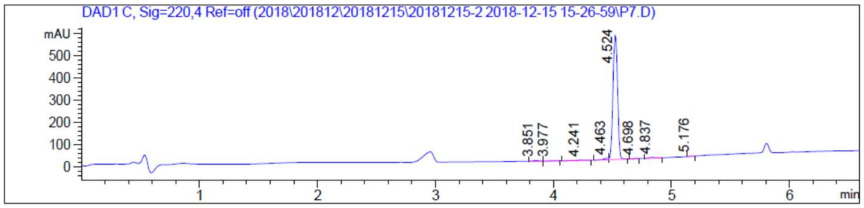

#	Meas. Ret. Time	Height	Height %	Area	Area %
1	3.851	3.420	0.585	10.485	0.665
2	3.977	1.560	0.267	5.856	0.372
3	4.241	2.681	0.458	12.983	0.824
4	4.463	6.693	1.144	21.922	1.391
5	4.524	563.717	96.389	1501.922	95.280
6	4.698	1.048	0.179	2.754	0.175
7	4.837	3.796	0.649	15.872	1.007
8	5.176	1.921	0.328	4.526	0.287

400MHz 1H-NMR P7 IN CDCL3

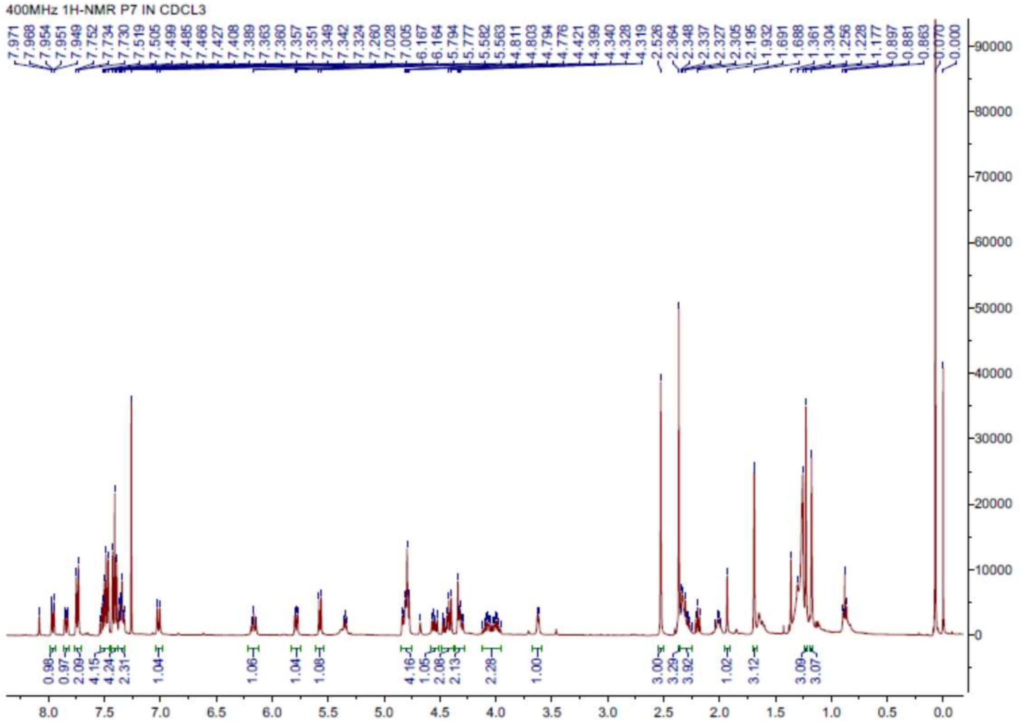

100MHz 13C-NMR P7 IN CDCL3

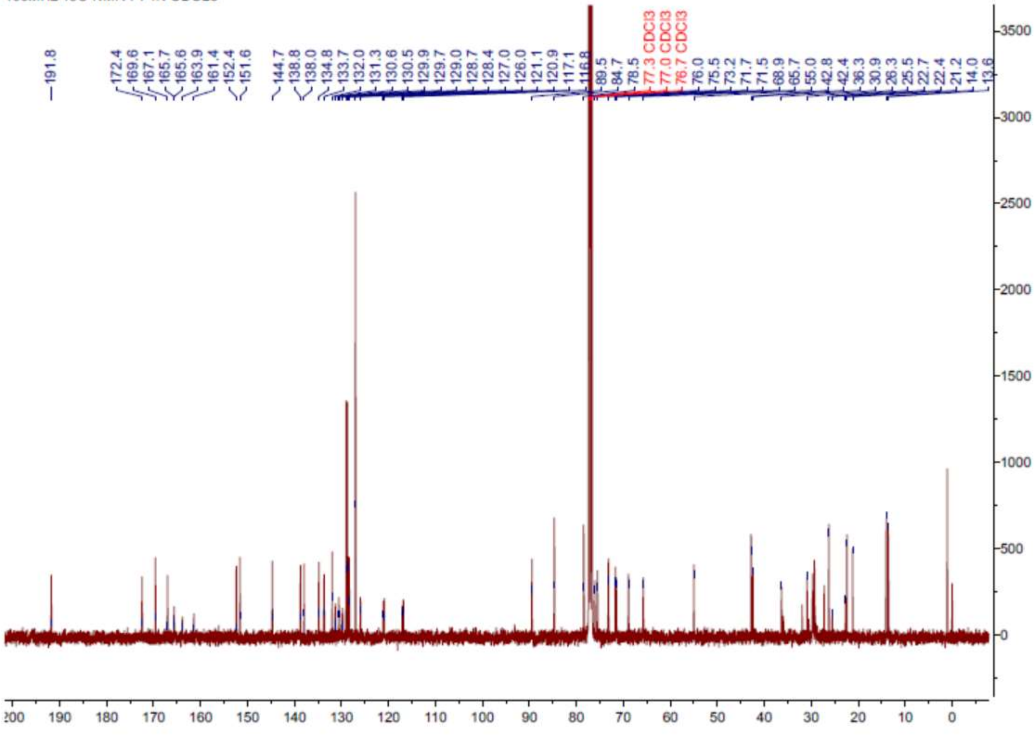

ESI-MS, HPLC, ^{1}H-NMR and ^{13}C-NMR spectra of compound **21d**

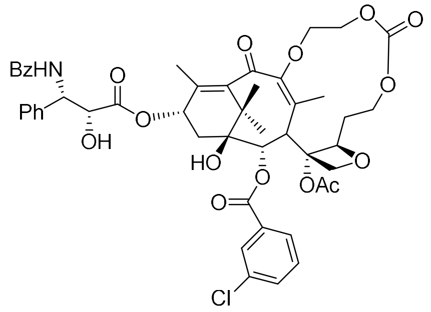

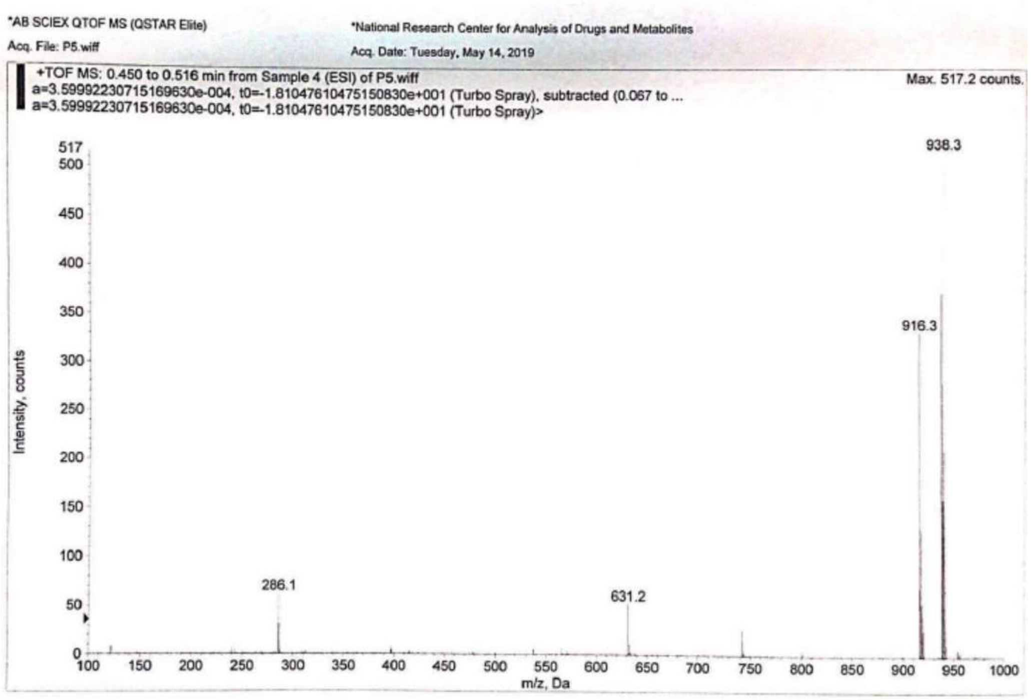

```
Sample Name       : P5
Injection Date    : Fri, 14. Dec. 2018                      Seq. Line :    29
Inj. Volumn       : 20.0 ul
Acq Operator      :
Acq   Method      : F:\DATA\2018\201812\20181214\20181213-2 2018-12-14 08-22-34\MTH-GEN-
                    MS.M
                    HPLC Analysis Conditions

1. Column        : XDB C18  4.6*50mm  1.8um
2. Mobile Phase :  A:Water+0.05%TFA    B :ACN+0.05%TFA
                  Time              A%                   B%
3. Flow Mode     : 0.00             90.0                 10.0
                  4.50              0.0                  100.0
                  6.50              0.0                  100.0
                  6.60              90.0                 10.0
 4. Flow          :   Start Flow :  1.0  ml/min

 5. UV Wavelength:   C: 220nm ;

 6. Column Temp. :   Left :   40.0C    Right :     40.0C

 7. Sample Preparation:
```

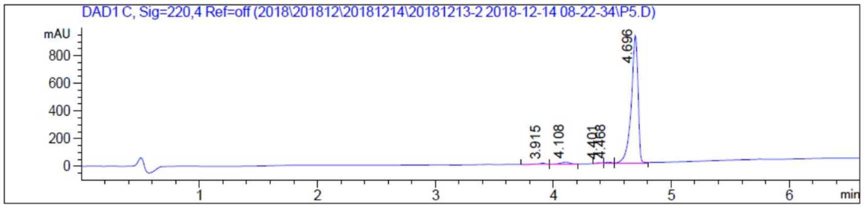

#	Meas. Ret. Time	Height	Height %	Area	Area %
1	3.915	7.311	0.765	34.270	0.922
2	4.108	14.869	1.555	68.751	1.850
3	4.401	2.866	0.300	8.024	0.216
4	4.468	5.583	0.584	14.402	0.388
5	4.696	925.632	96.797	3590.721	96.624

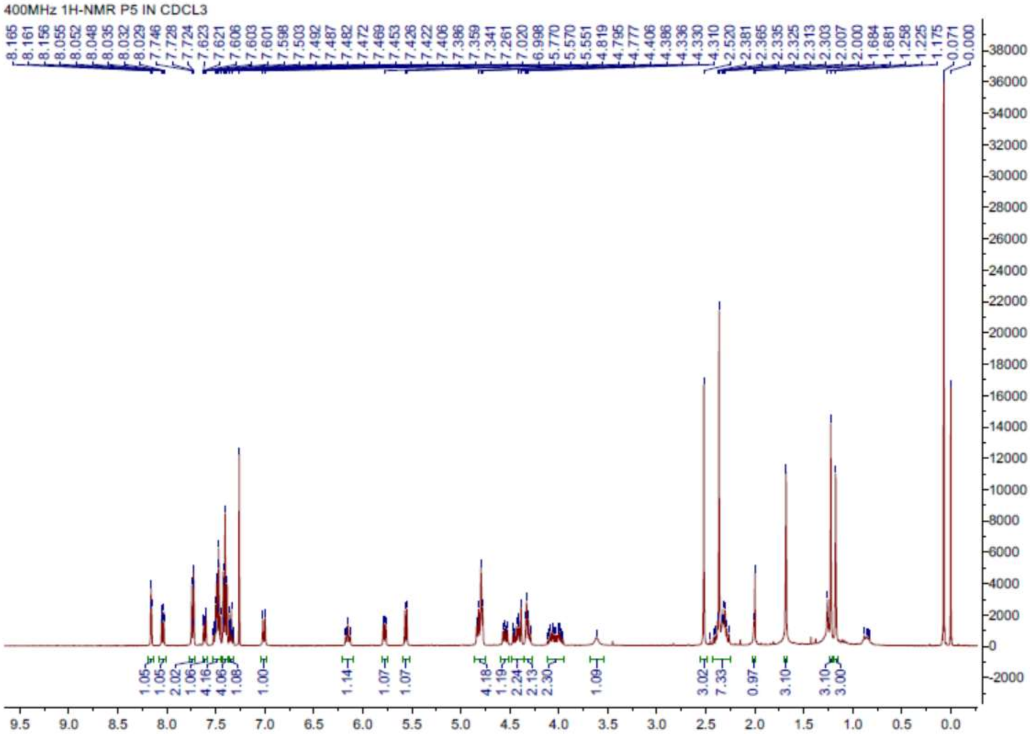
400MHz 1H-NMR P5 IN CDCL3
8.165 8.161 8.156 8.055 8.052 8.048 8.035 8.032 8.029 7.746 7.728 7.724 7.623 7.621 7.606 7.603 7.601 7.598 7.503 7.492 7.487 7.482 7.472 7.469 7.453 7.426 7.422 7.406 7.386 7.359 7.341 7.261 7.020 6.998 5.770 5.570 5.551 4.819 4.795 4.777 4.406 4.386 4.336 4.330 4.310 2.520 2.381 2.365 2.335 2.325 2.313 2.303 2.007 2.000 1.684 1.681 1.258 1.225 1.175 0.071 0.000
1.05 1.05 2.02 1.06 4.16 4.06 1.08 1.00 1.14 1.07 1.07 4.18 1.19 2.24 2.13 2.30 1.09 3.02 7.33 0.97 3.10 3.10 3.00

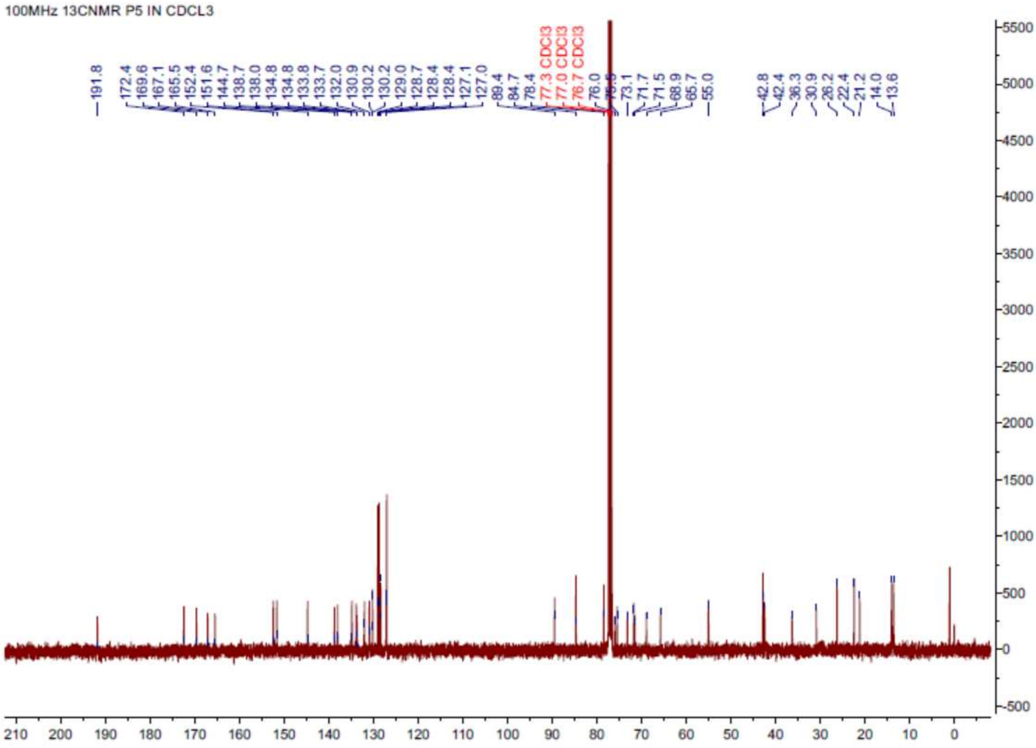
100MHz 13CNMR P5 IN CDCL3
191.8 172.4 169.6 167.1 165.5 152.4 151.6 144.7 138.7 138.0 134.8 134.8 133.8 133.7 132.0 130.9 130.2 130.2 129.0 128.7 128.4 128.4 127.1 127.0 89.4 84.7 78.4 77.3 CDCl3 77.0 CDCl3 76.7 CDCl3 76.0 73.1 71.7 71.5 68.9 65.7 55.0 42.8 42.4 36.3 30.9 26.2 22.4 21.2 14.0 13.6

ESI-MS, HPLC, ^{1}H NMR and ^{13}C NMR spectra of compound **21e**

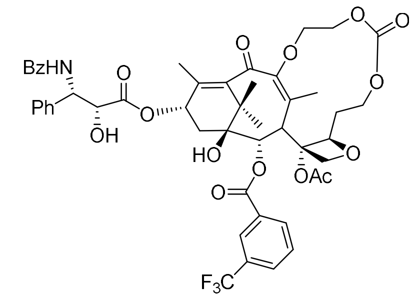

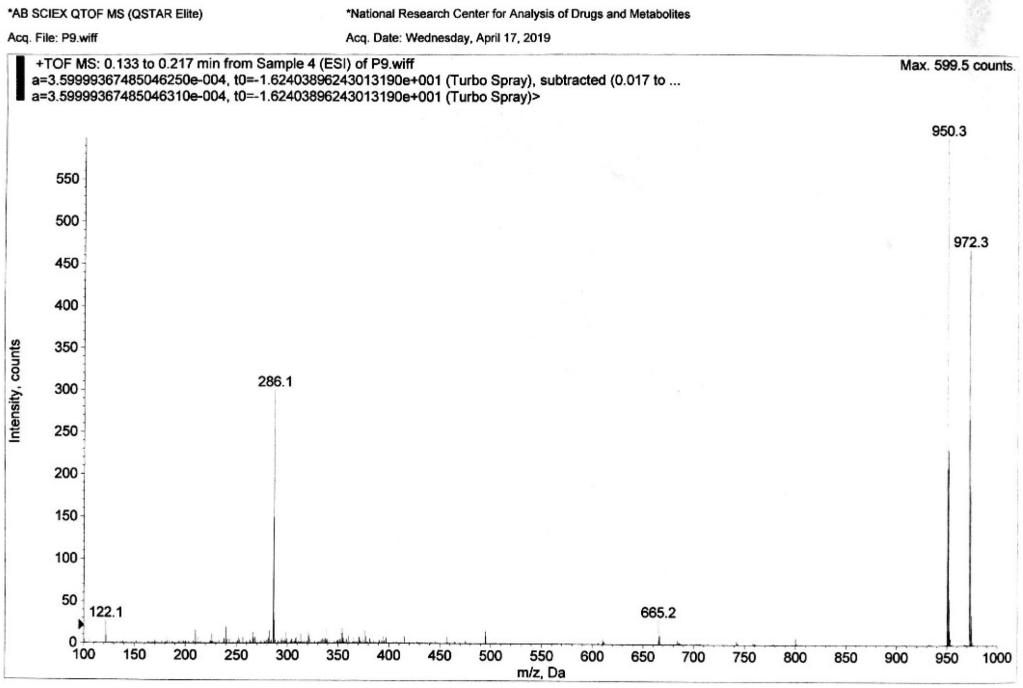

```
Sample Name       : P9
Injection Date    : Fri, 14. Dec. 2018                     Seq. Line :   39
Inj. Volumn       : 20.0 ul
Acq Operator      :
Acq   Method      : F:\DATA\2018\201812\20181214\20181213-2 2018-12-14 08-22-34\MTH-GEN-
                    MS.M
                    HPLC Analysis Conditions

1. Column        : XDB C18  4.6*50mm  1.8um
2. Mobile Phase :  A:Water+0.05%TFA    B :ACN+0.05%TFA
                  Time              A%                  B%
3. Flow Mode     : 0.00             90.0                10.0
                  4.50              0.0                 100.0
                  6.50              0.0                 100.0
                  6.60              90.0                10.0
 4. Flow          :   Start Flow :  1.0  ml/min

 5. UV Wavelength:   C: 220nm ;

 6. Column Temp. :   Left :   40.0C    Right :     40.0C

 7. Sample Preparation:
```

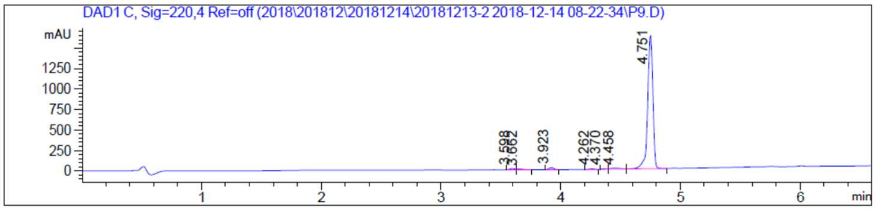

#	Meas. Ret. Time	Height	Height %	Area	Area %
1	3.598	17.020	0.987	46.769	0.910
2	3.662	12.113	0.702	37.871	0.737
3	3.923	31.305	1.815	83.825	1.631
4	4.262	8.459	0.490	23.686	0.461
5	4.370	6.397	0.371	14.278	0.278
6	4.458	11.221	0.650	46.630	0.907
7	4.751	1638.653	94.985	4886.958	95.077

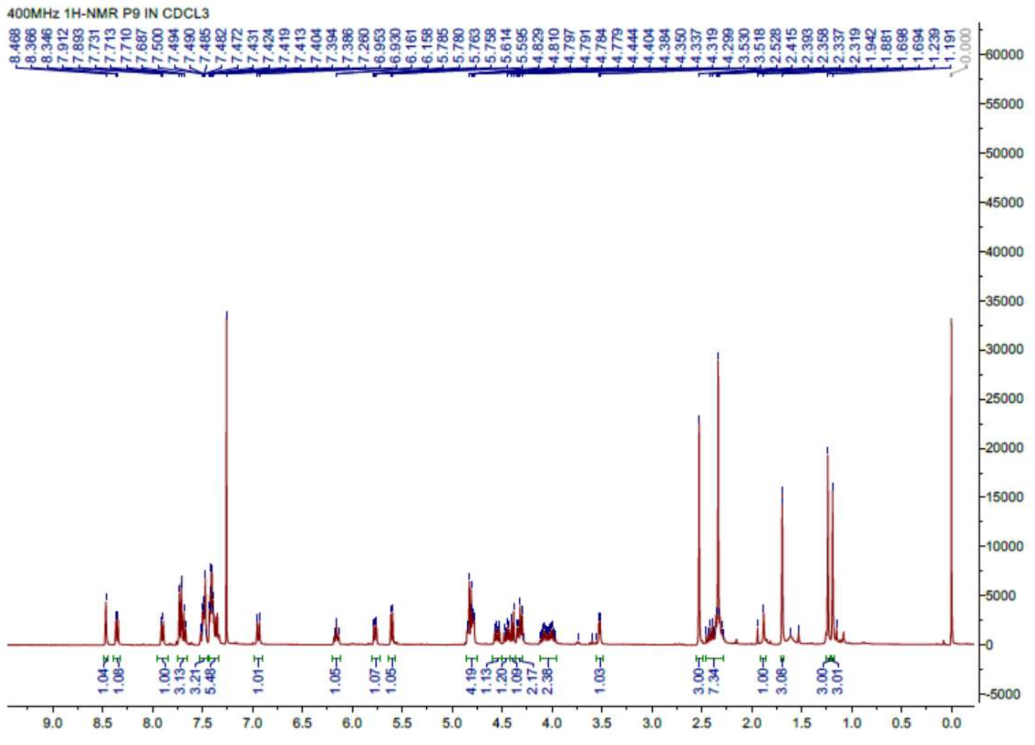
400MHz 1H-NMR P9 IN CDCL3

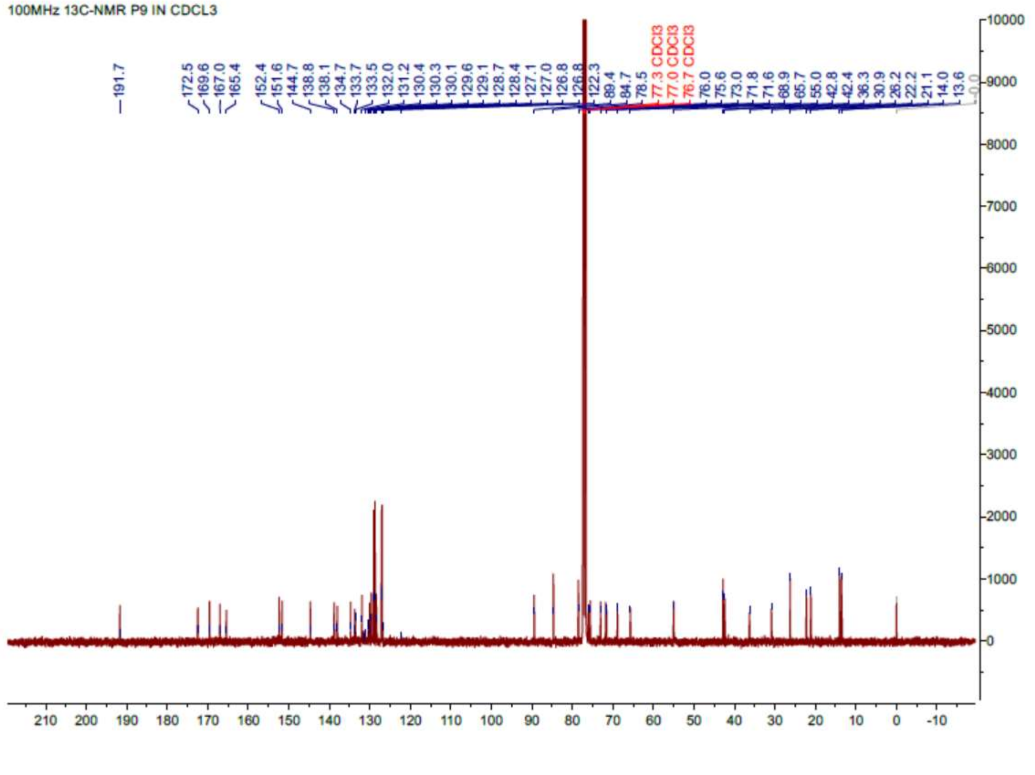
100MHz 13C-NMR P9 IN CDCL3

ESI-MS, HPLC, ^{1}H NMR and ^{13}C NMR spectra of compound **22a**

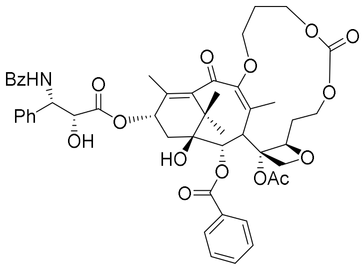

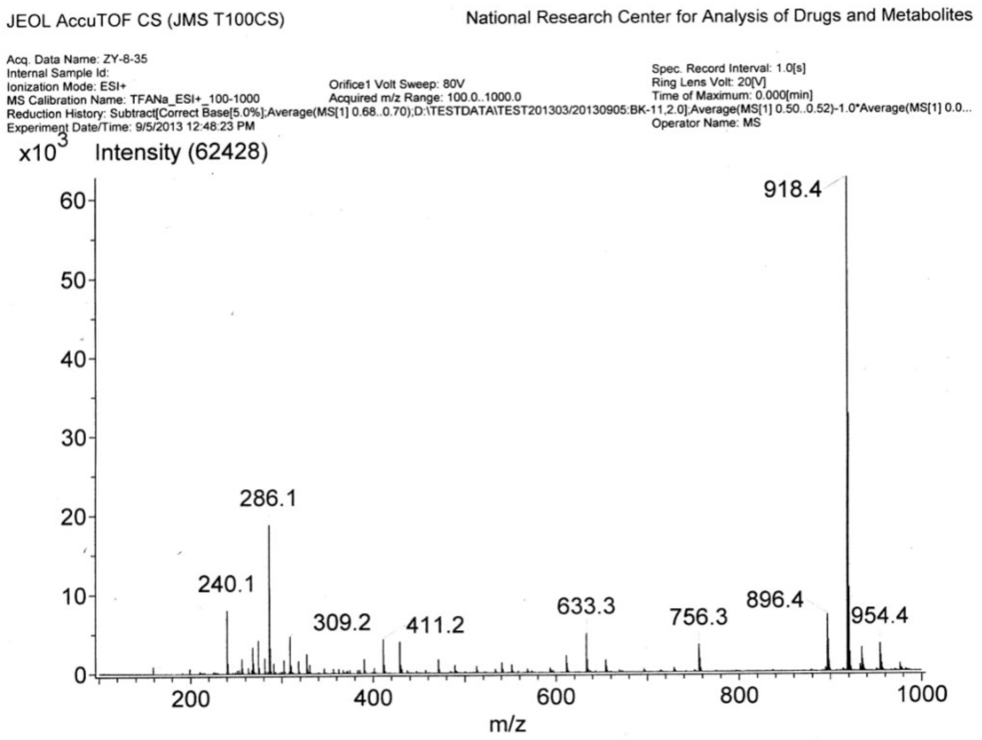

```
Sample Name       : P2
Injection Date    : Thu, 20. Dec. 2018                    Seq. Line :   13
Inj. Volumn       : 10.0 ul
Acq Operator      :
Acq   Method      : F:\DATA\2018\201812\20181220\20181220-3 2018-12-20 19-01-52\MTH-GEN-
                    MS.M
                    HPLC Analysis Conditions

1. Column        : XDB C18  4.6*50mm  1.8um
2. Mobile Phase :  A:Water+0.05%TFA    B :ACN+0.05%TFA
                   Time              A%                B%
3. Flow Mode     : 0.00              90.0              10.0
                   4.50              0.0               100.0
                   6.50              0.0               100.0
                   6.60              90.0              10.0
 4. Flow         :   Start Flow :  1.0  ml/min

 5. UV Wavelength:   C: 220nm ;

 6. Column Temp. :   Left :   40.0C    Right :     40.0C

 7. Sample Preparation:
```

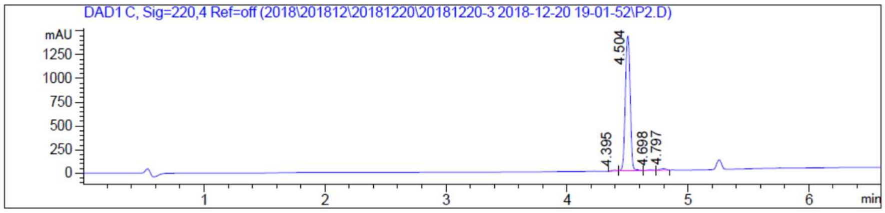

#	Meas. Ret. Time	Height	Height %	Area	Area %
1	4.395	8.093	0.554	19.549	0.490
2	4.504	1430.789	97.886	3900.252	97.709
3	4.698	7.281	0.498	19.407	0.486
4	4.797	15.528	1.062	52.494	1.315

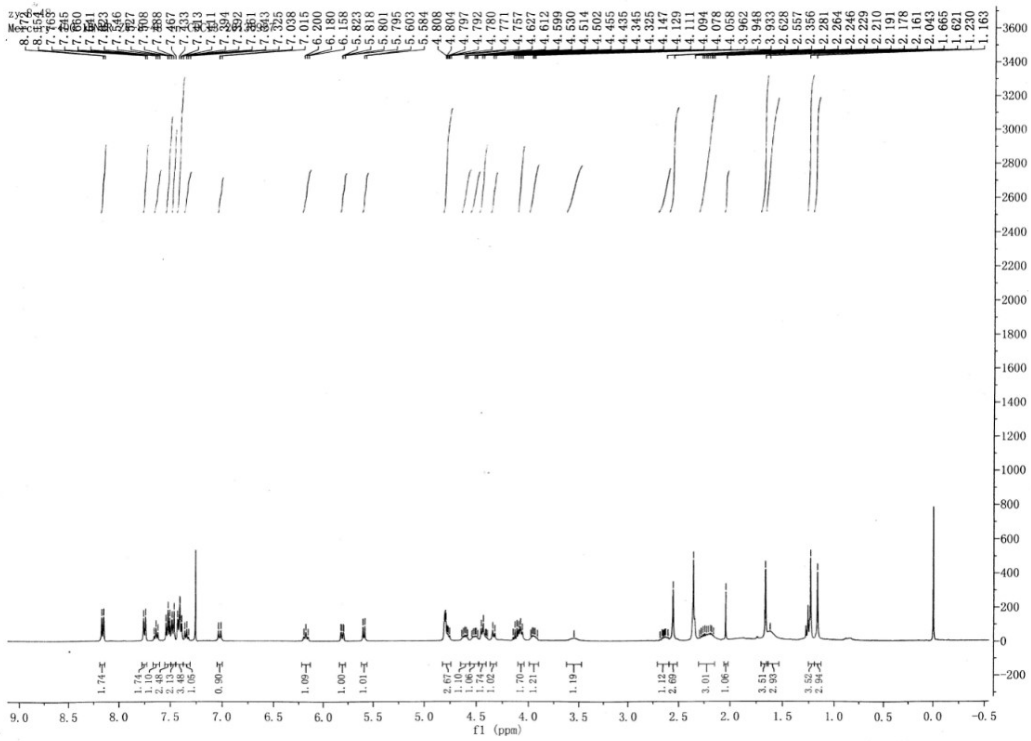

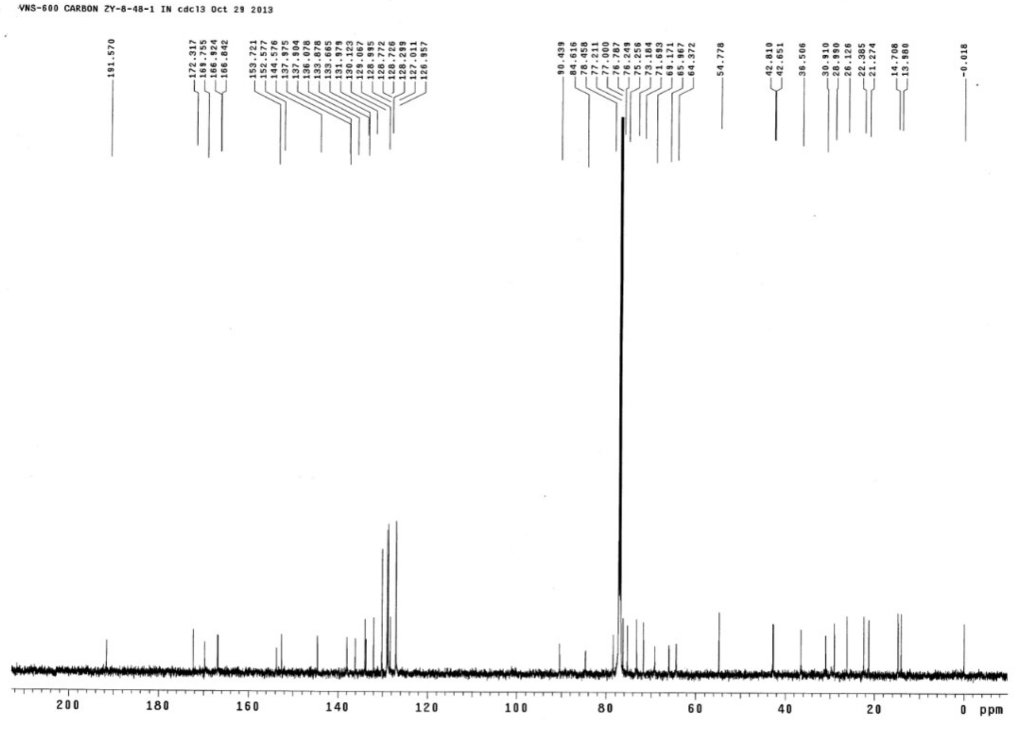
VNS-600 CARBON ZY-8-48-1 IN cdcl3 Oct 29 2013

ESI-MS, HPLC, ^{1}H NMR and ^{13}C NMR spectra of compound **22b**

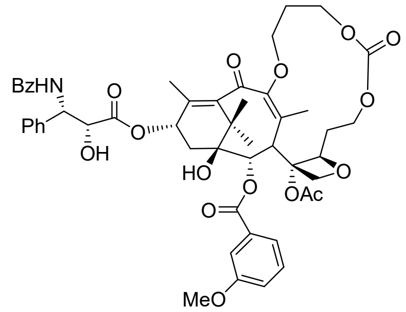

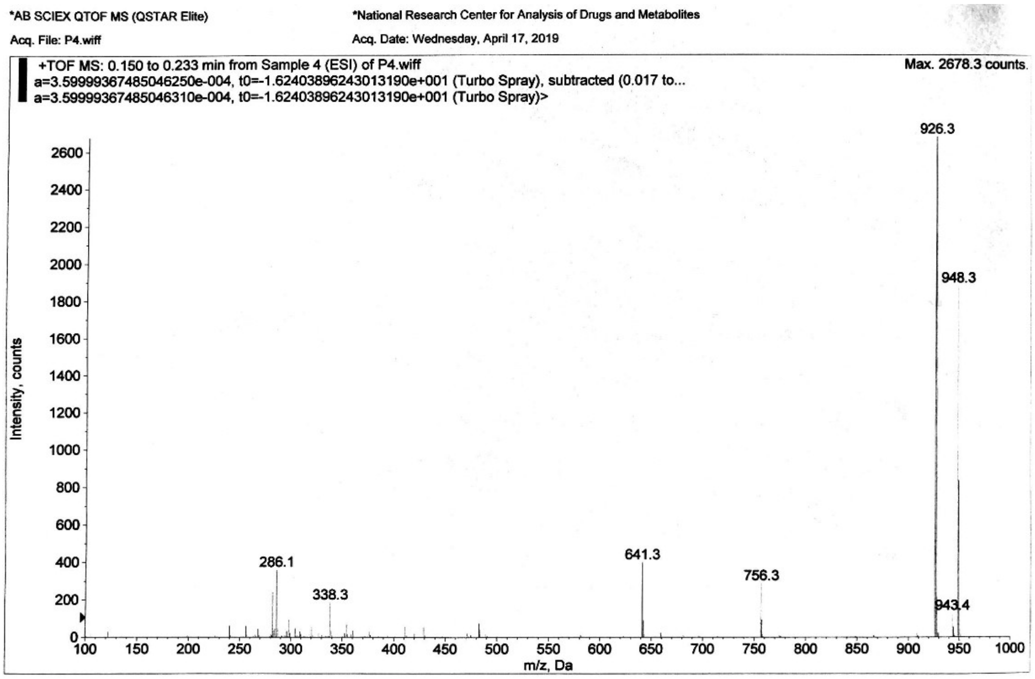

```
Sample Name       : P4
Injection Date    : Wed, 26. Dec. 2018                    Seq. Line :   1
Inj. Volumn       : 20.0 ul
Acq Operator      :
Acq   Method      : F:\DATA\2018\201812\20181225\20181225-1 2018-12-26 11-41-49\MTH-GEN-
                    MS.M
                    HPLC Analysis Conditions

1. Column        : XDB C18  4.6*50mm  1.8um
2. Mobile Phase :  A:Water+0.05%TFA     B :ACN+0.05%TFA
                   Time              A%                  B%
3. Flow Mode     : 0.00              90.0                10.0
                   4.50              0.0                 100.0
                   6.50              0.0                 100.0
                   6.60              90.0                10.0
 4. Flow          :   Start Flow :  1.0  ml/min

 5. UV Wavelength:   C: 220nm ;

 6. Column Temp. :   Left :    40.0C     Right :     40.0C

 7. Sample Preparation:
```

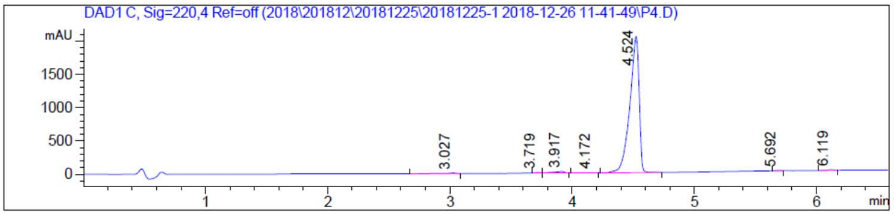

#	Meas. Ret. Time	Height	Height %	Area	Area %
1	3.027	10.339	0.491	46.267	0.420
2	3.719	6.438	0.306	15.240	0.138
3	3.917	25.214	1.197	145.007	1.315
4	4.172	2.467	0.117	12.605	0.114
5	4.524	2047.503	97.211	10768.203	97.647
6	5.692	5.343	0.254	14.518	0.132
7	6.119	8.947	0.425	25.864	0.235

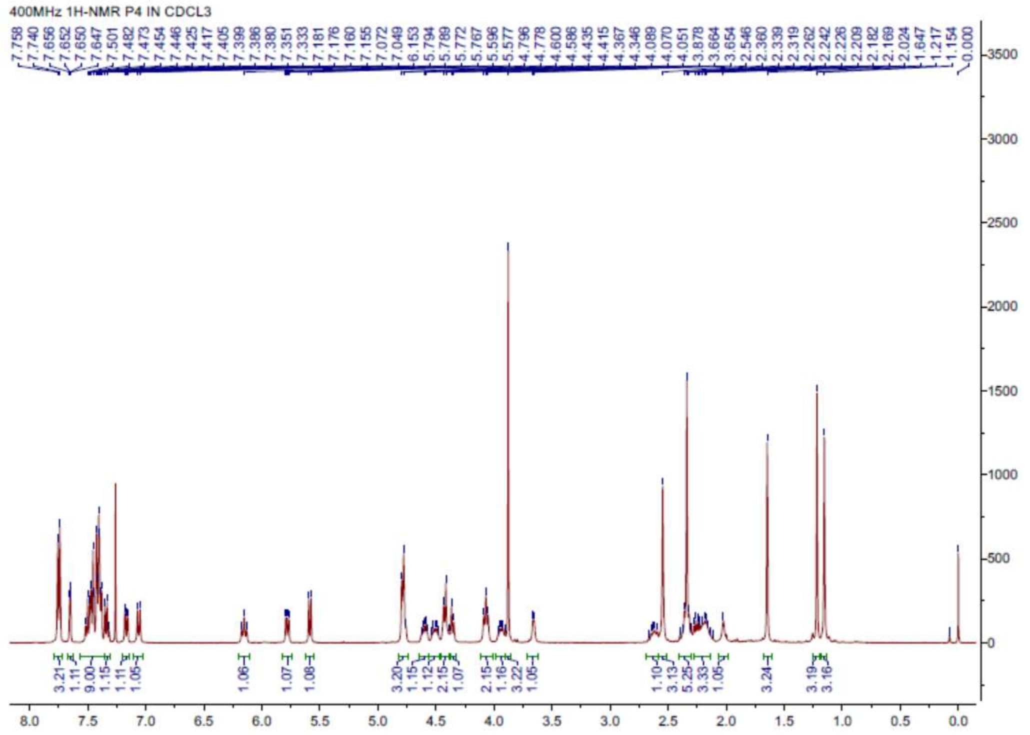
400MHz 1H-NMR P4 IN CDCL3

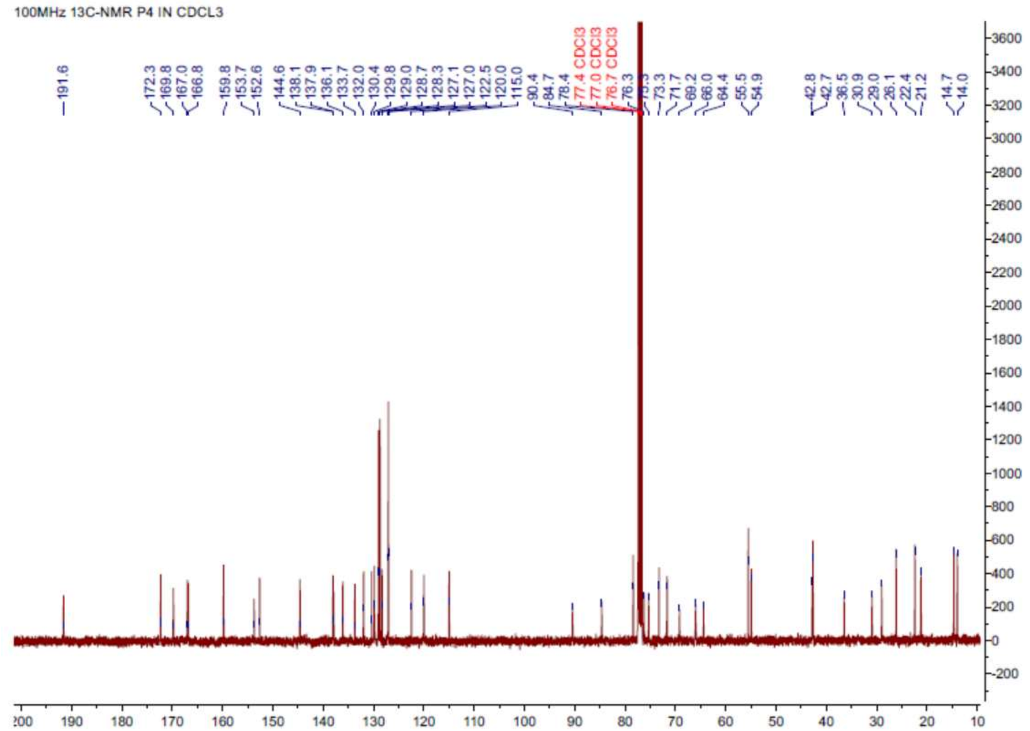
100MHz 13C-NMR P4 IN CDCL3

ESI-MS, HPLC, ^{1}H-NMR and ^{13}C-NMR spectra of compound **22c**

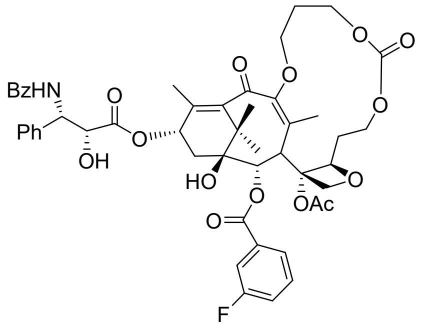

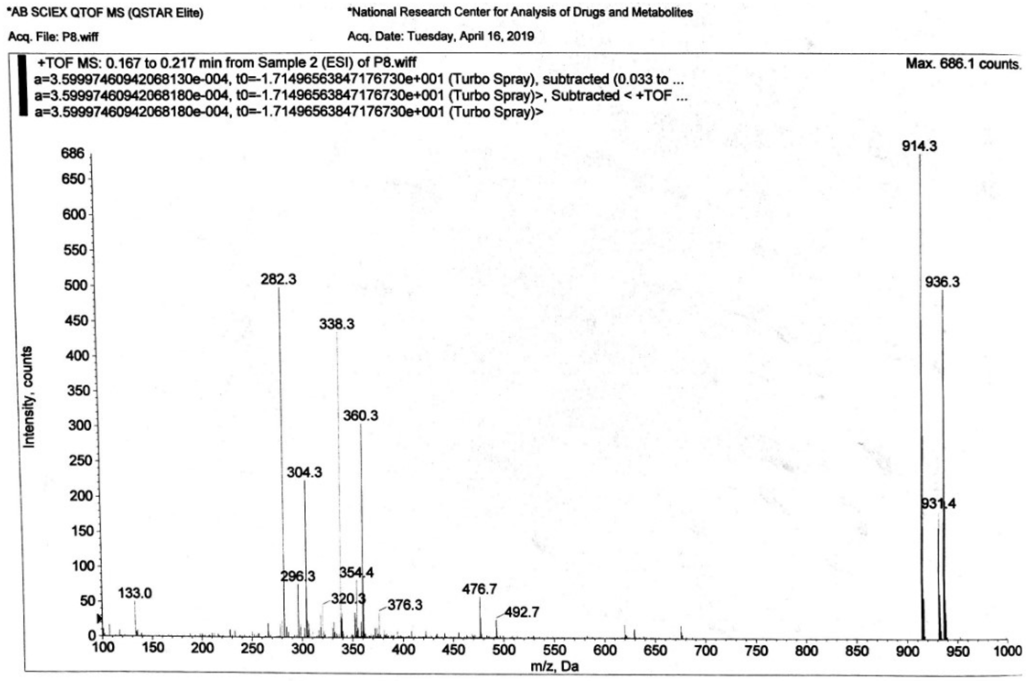

Sample Name : P8
Injection Date : Tue, 11. Dec. 2018 Seq. Line : 2
Inj. Volumn : 20.0 ul
Acq Operator :
Acq Method : F:\DATA\2018\201812\20181210\20181206-3 2018-12-11 13-57-34\MTH-GEN-MS.M
HPLC Analysis Conditions

1. Column : XDB C18 4.6*50mm 1.8um
2. Mobile Phase : A:Water+0.05%TFA B :ACN+0.05%TFA

	Time	A%	B%
3. Flow Mode :	0.00	90.0	10.0
	4.50	0.0	100.0
	6.50	0.0	100.0
	6.60	90.0	10.0

4. Flow : Start Flow : 1.0 ml/min

5. UV Wavelength: C: 220nm ;

6. Column Temp. : Left : 40.0C Right : 40.0C

7. Sample Preparation:

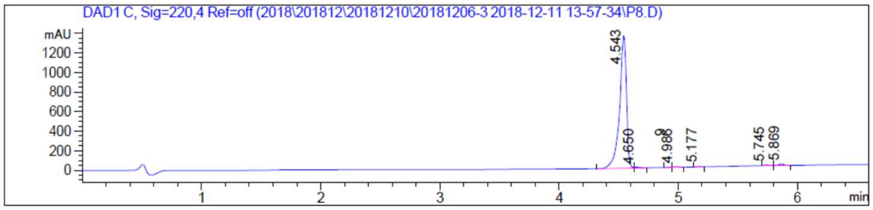

#	Meas. Ret. Time	Height	Height %	Area	Area %
1	4.543	1363.175	96.297	5409.773	97.275
2	4.650	8.546	0.604	27.399	0.493
3	4.929	4.347	0.307	13.001	0.234
4	4.986	9.127	0.645	29.162	0.524
5	5.177	6.224	0.440	16.542	0.297
6	5.745	8.868	0.626	23.211	0.417
7	5.869	15.308	1.081	42.210	0.759

400MHz 1H-NMR P8 IN CDCL3

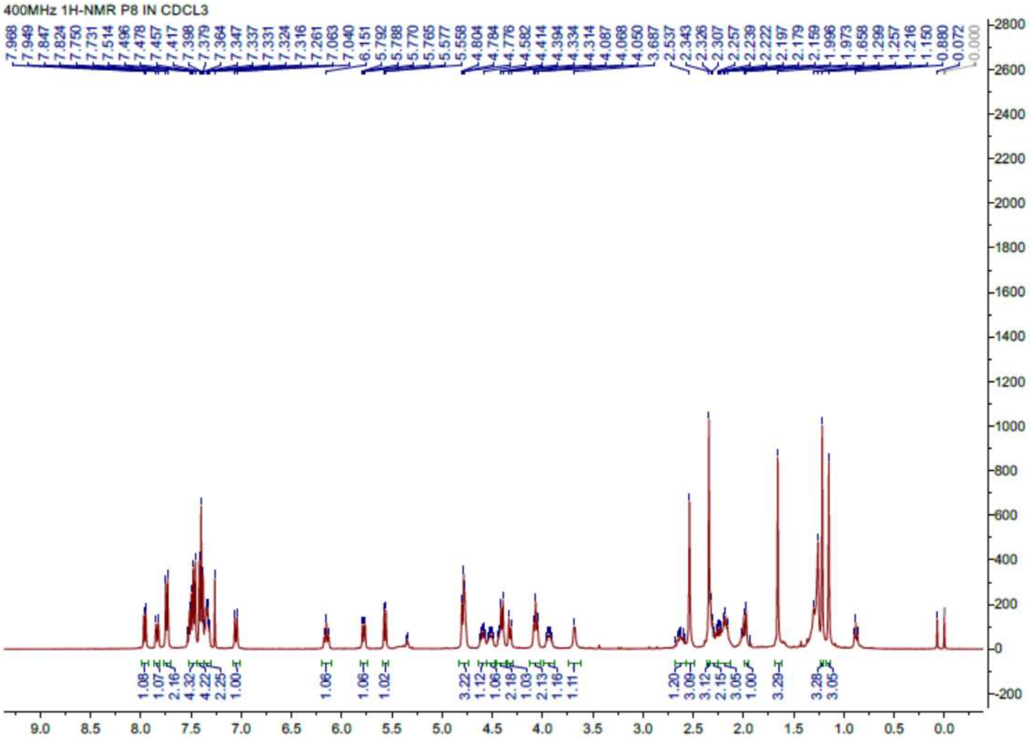

100MHz 13C-NMR P8 IN CDCL3

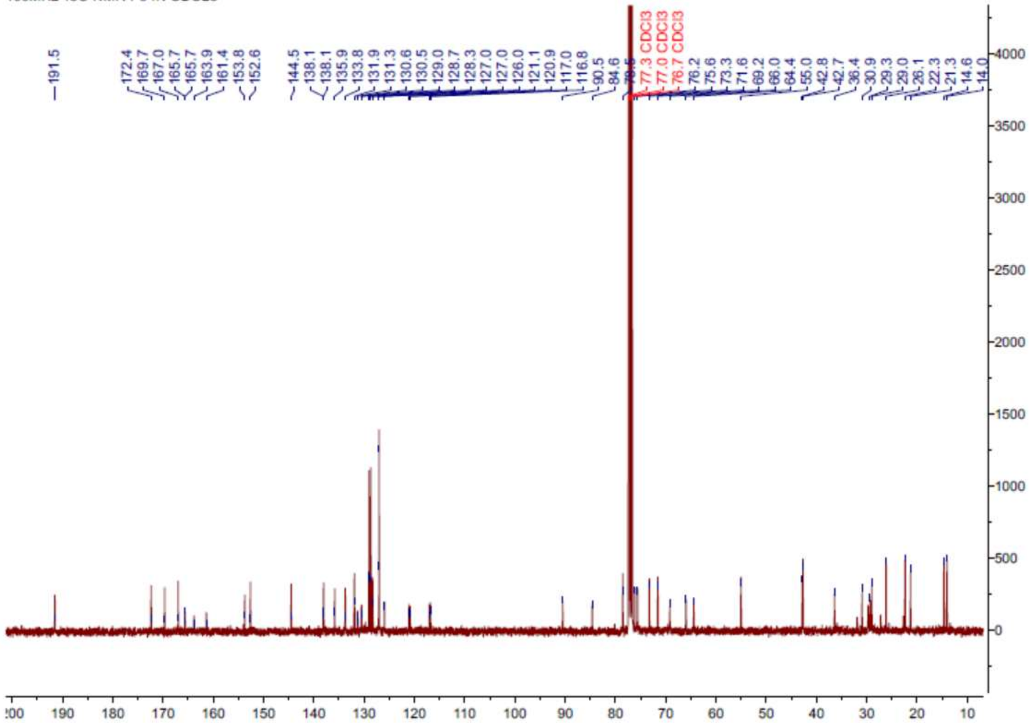

ESI-MS, HPLC, ^{1}H-NMR and ^{13}C-NMR spectra of compound **22d**

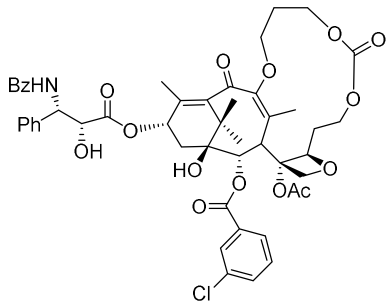

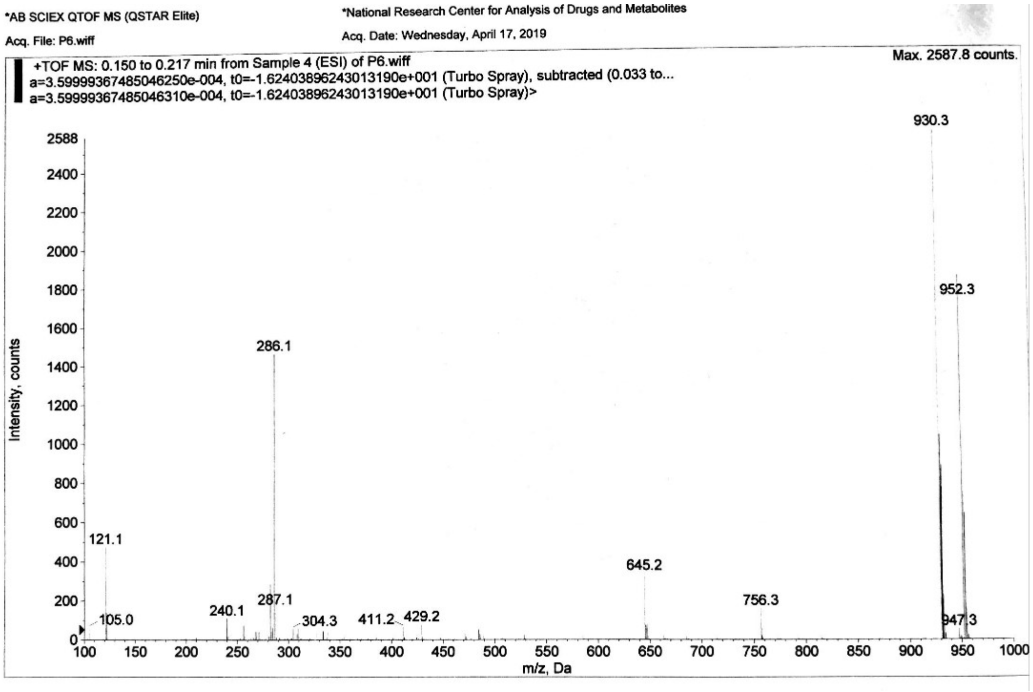

```
Sample Name       : P6
Injection Date    : Fri, 14. Dec. 2018                    Seq. Line :   31
Inj. Volumn       : 20.0 ul
Acq Operator      :
Acq   Method      : F:\DATA\2018\201812\20181214\20181213-2 2018-12-14 08-22-34\MTH-GEN-
                    MS.M
                    HPLC Analysis Conditions

1. Column        : XDB C18  4.6*50mm  1.8um
2. Mobile Phase :  A:Water+0.05%TFA    B :ACN+0.05%TFA
                  Time              A%                 B%
3. Flow Mode     : 0.00             90.0               10.0
                  4.50              0.0                100.0
                  6.50              0.0                100.0
                  6.60              90.0               10.0
 4. Flow         :   Start Flow :  1.0  ml/min

 5. UV Wavelength:   C: 220nm ;

 6. Column Temp. :   Left :   40.0C    Right :    40.0C

 7. Sample Preparation:
```

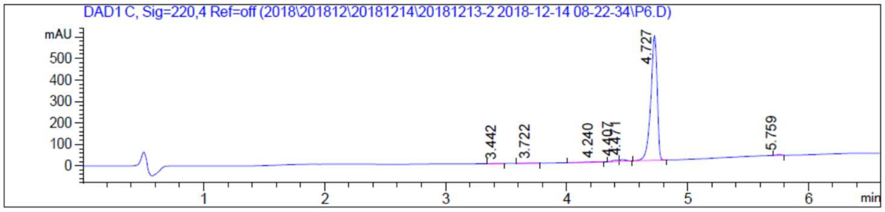

#	Meas. Ret. Time	Height	Height %	Area	Area %
1	3.442	1.103	0.181	3.654	0.165
2	3.722	2.003	0.330	9.459	0.426
3	4.240	2.084	0.343	14.781	0.666
4	4.407	6.704	1.103	22.202	1.000
5	4.471	9.064	1.491	31.069	1.399
6	4.727	582.526	95.836	2128.743	95.851
7	5.759	4.352	0.716	10.987	0.495

4O0MHz 1H-NMR P6 IN CDCL3

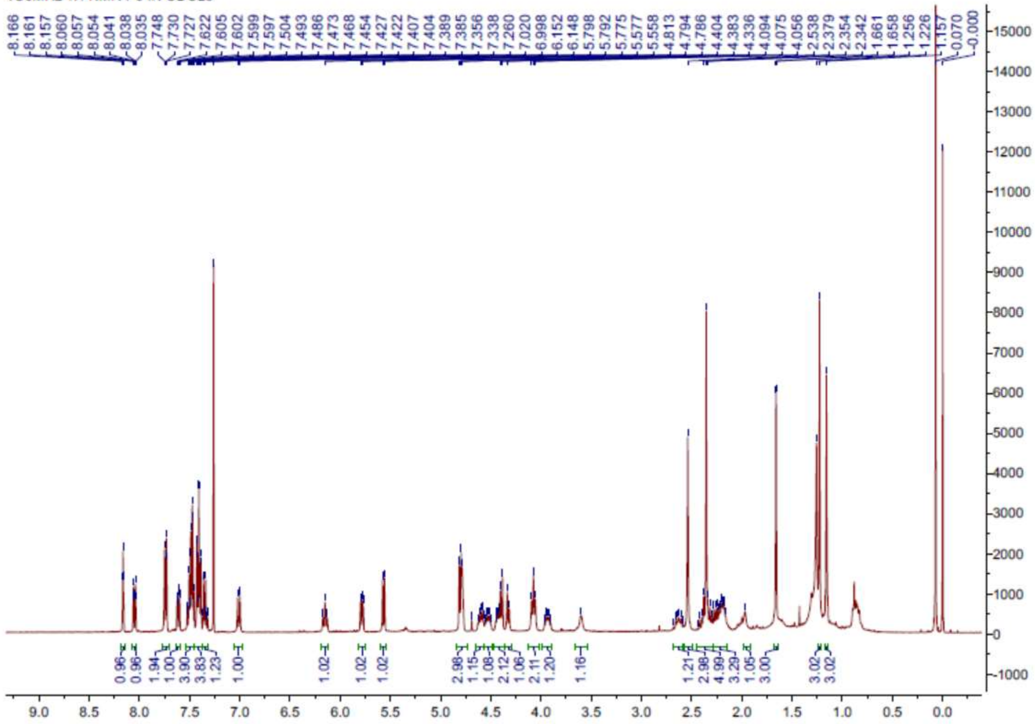

100MHz 13C-NMR P6 IN CDCL3

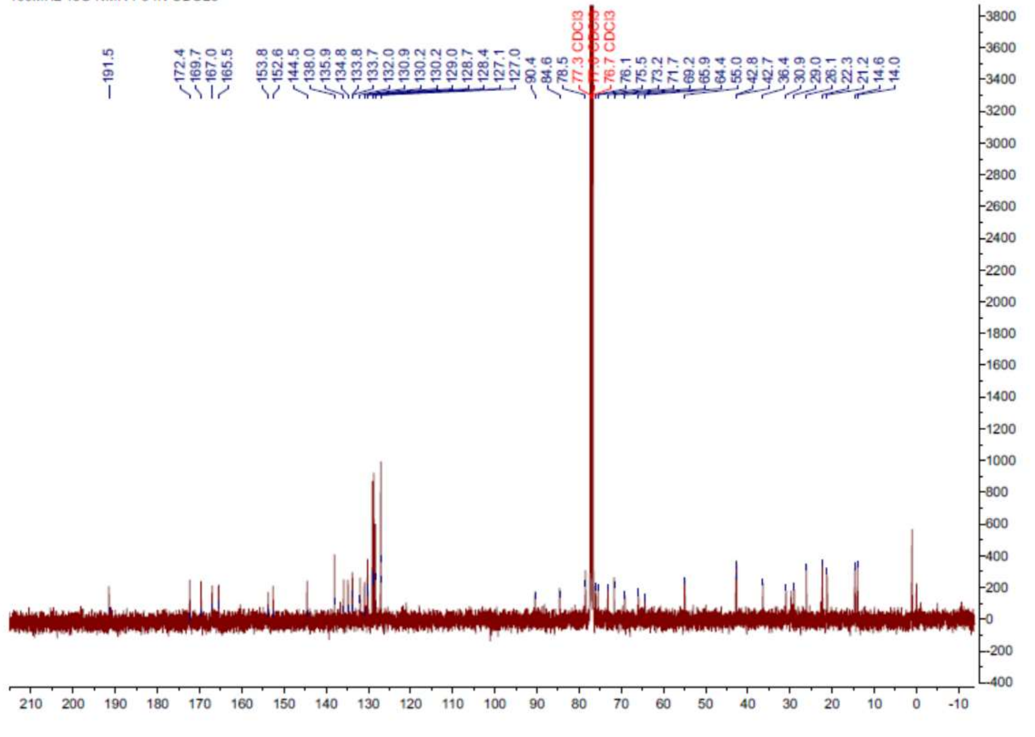

ESI-MS, HPLC, ^{1}H-NMR and ^{13}C-NMR spectra of compound **22e**

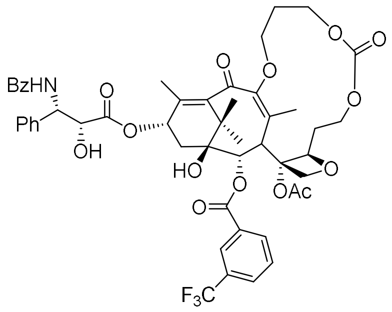

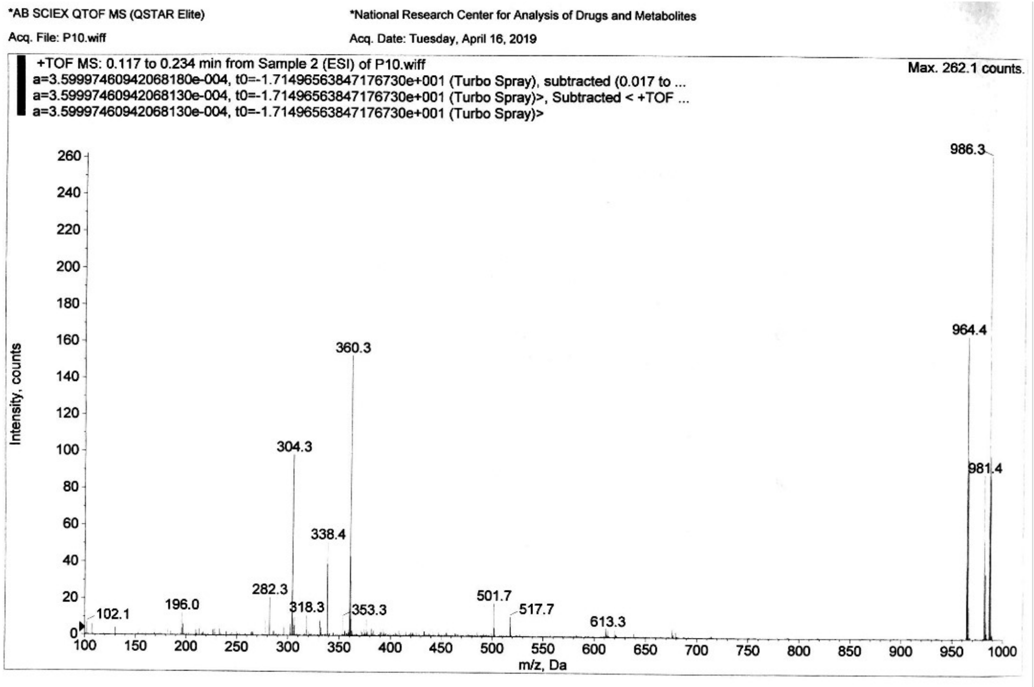

```
Sample Name       : P10
Injection Date    : Fri, 7. Dec. 2018                      Seq. Line :   20
Inj. Volumn       : 15.0 ul
Acq Operator      :
Acq   Method      : F:\DATA\2018\201812\20181205\20181206-3 2018-12-07 08-53-22\MTH-GEN-
                    MS.M
                    HPLC Analysis Conditions

1. Column        : XDB C18  4.6*50mm  1.8um
2. Mobile Phase :  A:Water+0.05%TFA     B :ACN+0.05%TFA
                  Time               A%                 B%
3. Flow Mode     : 0.00             90.0               10.0
                  4.50              0.0               100.0
                  6.50              0.0               100.0
                  6.60             90.0                10.0
 4. Flow          :   Start Flow :  1.0  ml/min

 5. UV Wavelength:   C: 220nm ;

 6. Column Temp. :   Left :   40.0C    Right :     40.0C

 7. Sample Preparation:
```

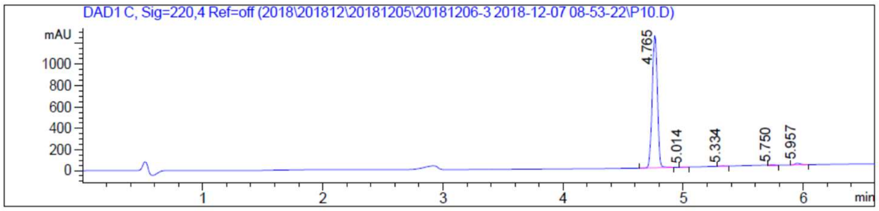

#	Meas. Ret. Time	Height	Height %	Area	Area %
1	4.765	1246.186	97.861	3744.757	97.945
2	5.014	1.940	0.152	5.068	0.133
3	5.334	3.636	0.286	9.422	0.246
4	5.750	2.668	0.210	6.397	0.167
5	5.957	18.993	1.491	57.681	1.509

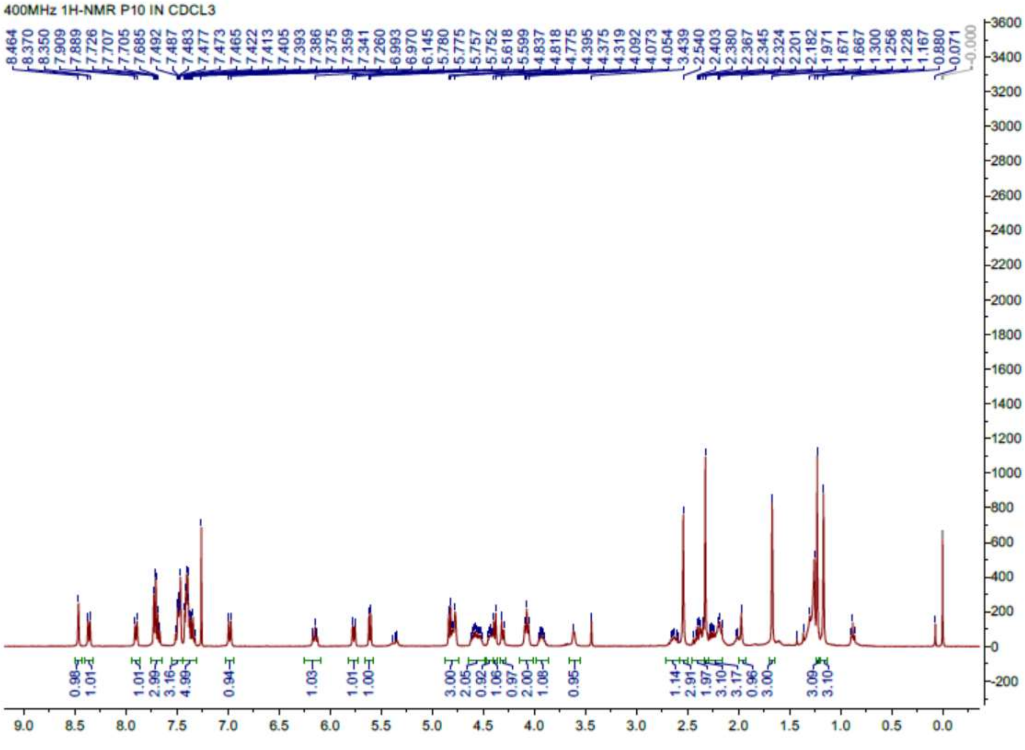

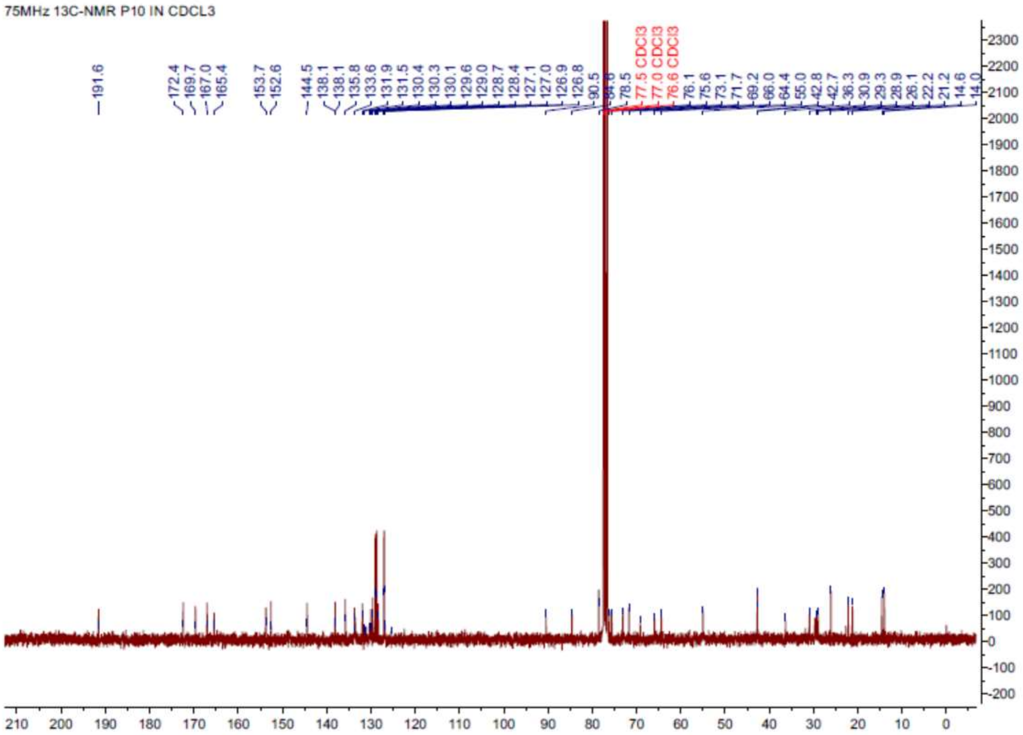
75MHz 13C-NMR P10 IN CDCL3
191.6
172.4
169.7
167.0
165.4
153.7
152.6
144.5
138.1
138.1
135.8
133.6
131.9
131.5
130.4
130.3
130.1
129.6
129.0
128.7
128.4
127.1
127.0
126.9
126.8
90.5
84.6
78.5
77.5 CDCl3
77.0 CDCl3
76.6 CDCl3
76.1
75.6
73.1
71.7
69.2
66.0
64.4
55.0
42.8
42.7
36.3
30.9
29.3
28.9
26.1
22.2
21.2
14.6
14.0
